# The Influence of Long Carbon Chains on the Antioxidant and Anticancer Properties of *N*-Substituted Benzisoselenazolones and Corresponding Diselenides

**DOI:** 10.3390/ph16111560

**Published:** 2023-11-04

**Authors:** Agata J. Pacuła-Miszewska, Magdalena Obieziurska-Fabisiak, Aneta Jastrzębska, Angelika Długosz-Pokorska, Katarzyna Gach-Janczak, Jacek Ścianowski

**Affiliations:** 1 Department of Organic Chemistry, Faculty of Chemistry, Nicolaus Copernicus University, 7 Gagarin Street, 87-100 Torun, Poland; magdao@umk.pl (M.O.-F.); jsch@umk.pl (J.Ś.); 2 Department of Analytical Chemistry and Applied Spectroscopy, Faculty of Chemistry, Nicolaus Copernicus University in Torun, 7 Gagarin Street, 87-100 Torun, Poland; aj@umk.pl; 3 Department of Biomolecular Chemistry, Faculty of Medicine, Medical University of Lodz, Mazowiecka 6/8, 92-215 Lodz, Poland; angelika.dlugosz@umed.lodz.pl (A.D.-P.); katarzyna.gach@umed.lodz.pl (K.G.-J.)

**Keywords:** benzisoselenazol-3(2*H*)-ones, diselenides, antioxidant activity, antiproliferative activity

## Abstract

Organoselenium compounds are well-known for their numerous biocapacities, which result from the uniqueness of the selenium atom and the possibility of constructing heterorganic molecules that can mimic the activity of selenoenzymes, crucial for a multitude of important physiological processes. In this paper, we have synthesized a series of *N*-substituted benzisoselenazolones and corresponding diphenyl diselenides possessing lipophilic long carbon chains, solely or with additional polar insets: phenyl linkers and ester groups. Evaluation of their antioxidant and cytotoxic activity revealed an increased H_2_O_2_-reduction potential of diphenyl diselenides bearing *N*-octyl, ethyl *N*-(12-dodecanoate)- and *N*-(8-octanoate) groups, elevated radical scavenging activity of 2,2′-diselenobis(*N*-dodecylbenzamide) and a promising cytotoxic potential of *N*-(4-dodecyl)phenylbenzisoselenazol-3(2*H*)-one.

## 1. Introduction

Reactive oxygen and nitrogen species (ROS, RNS) play a dual role in living organisms as they can cause both beneficial and harmful effects. They are necessary for several physiological functions like cell signaling regulation and immune responses, supporting cell survival. On the other hand, the excess of ROS/RNS can modify the structure and function of proteins, lipids and DNA, causing cellular damage that may lead to various diseases, including cancer, neurodegeneration, diabetes, cardiovascular disease and arthritis [1,2]. As the formation of ROS is an unavoidable result of the respiration of aerobic organisms, a natural antioxidant defense system has to exist to control the overproduction of reactive oxygen species, maintain redox homeostasis and inhibit oxidative stress. This essential role is played by a diversified system of endogenous and exogenous antioxidants that cooperate to restore the balance between the cellular oxidizing and reducing states. Endogenous antioxidants are classified as enzymatic (e.g., glutathione peroxidase, glutathione reductase, superoxide dismutase and catalase) and non-enzymatic metabolites (e.g., glutathione, lipoic acid, bilirubin, coenzyme Q10, L-arginine and melatonin) [3,4]. Exogenous antioxidants are those that have to be externally supplied, like vitamin C, flavonoids, carotenoids, polyphenols and unsaturated fatty acids [5]. 

**Figure 1:**
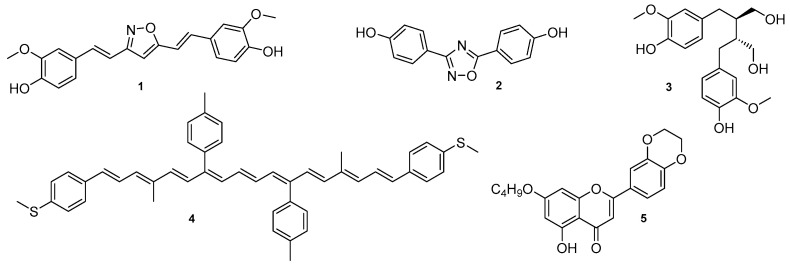
Examples of synthetic nature-inspired antioxidants.

The synthesis of artificial small-molecule antioxidants is currently a developing branch in synthetic organic chemistry. Structural modifications of natural radical scavengers or the design of new bioactive molecules with antioxidant potential enable the design of antioxidants with enhanced activity and bioavailability, along with minimized toxicity. Some examples of nature-inspired radical scavengers, including synthetic analogues of curcumin **1**, resveratrol **2**, lignan-based **3**, β-carotene **4** and flavonoid derivative **5**, are presented in Figure 1 [6].

Low molecular weight organoselenium compounds also occupy a well-established place in the group of artificial antioxidants [7]. The role of selenium itself as an essential dietary trace element has been known for a long time. The main organic forms of Se found in the human body are selenomethionine (SeMet) and selenocysteine (Sec). SeMet as the main dietary source of selenium, after incorporation into proteins, serves only as Se storage. Sec, on the other hand, builds up the active side of selenoenzymes possessing various biochemical functions. The reactivity of Sec is connected with its free selenol moiety, stable due to a specific chemical environment of the surrounding amino acids that form the final protein structure [8,9]. Until now, twenty-five selenoproteins have been identified in the human proteome, but only some have a recognized role in antioxidant protection, redox-state regulation and specific metabolic pathways. The most important member of these selenoproteins is glutathione peroxidase (GPx), which plays a major role in the organismal antioxidant defense mechanism protecting cells from oxidative stress [10]. The discovery of GPx inspired chemists to create derivatives that mimic this selenocysteine-based enzyme. The main representative for this group of compounds is the well-known radical scavenger ebselen (*N*-phenylbenzisoselenazol-3(2*H*)-one). The ability of its Se-N bond to easily cleave and convert to corresponding reactive forms like diselenide RSe-SeR or selenol RSeH enables the catalytic reduction of ROS similar to that expressed by GPx [11,12,13]. Although ebselen has made it to several clinical trials, including Meniere’s disease (phase 3), COVID-19 (phase 2) and hearing loss (phase 2), its low bioavailability and considerable toxicity initiate the search for more safe Se-therapeutics [14,15,16,17].

**Figure 2:**
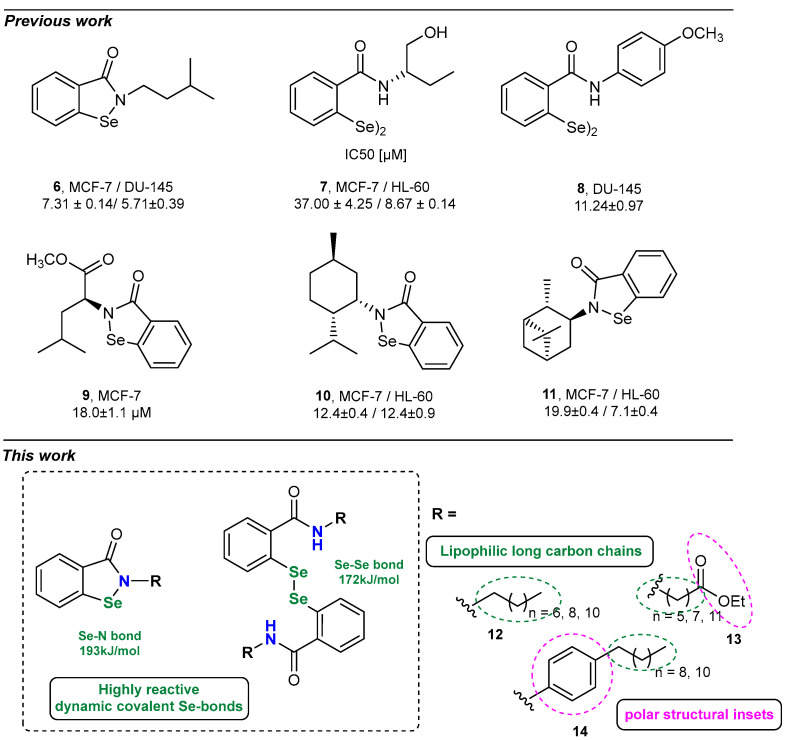
Examples of synthetic nature-inspired antioxidants.

In our previous work, we have modified the primary benzisoselenazolone core by attaching different moieties to the nitrogen atom, e.g., alkyl **6**, **7**, aryl **8**, chiral terpenyl **9**, **10** and aminoacid **11** substituents [18,19,20,21]. The antiproliferative activity of all compounds was evaluated, revealing promising anticancer potential. In this paper, we aimed to evaluate the influence of long carbon chains on the antioxidant and cytotoxic activity of *N*-functionalized benzisoselenazolones, by their direct attachment to the nitrogen atom **12**, through a phenyl ring as a linker **13** and with additional ester termination at the end of the chain **14**. The obtained compounds were also synthesized in the form of corresponding diselenides to check the reactivity for Se-functionalities with different bond energies (Se-Se 172 kJ/mol, Se-N bond 193 kJ/mol) [22,23] (Figure 2).

These modifications can provide structure–activity information concerning the influence of (a) lipophilic carbon chains that can increase solubility in organic solvents and interaction with the cell lipid bilayer membranes; (b) additional *N*-aromatic rings, which are often necessary for the efficient receptor binding, e.g., as *H. pylori* urease [24] or the main protease M^pro^ of SARS-CoV2 virus inhibitors [25,26]; and (c) ester groups that can improve solubility in water and increase the bioavailability of potential drugs [27,28].

## 2. Results and Discussion

**Scheme 1:**
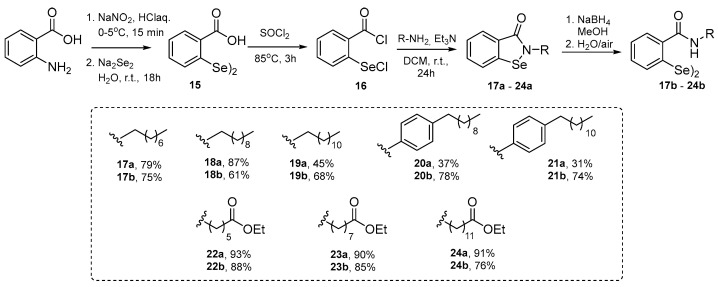
Synthetized benzisoselenazolones **17a**–**24a** and diselenides **17b**–**24b**.

The first stage of the research involved the synthesis of *N*-functionalized benzisoselenazol-3(2*H*)-ones **17a**–**24a** and corresponding diselenides **17b**–**24b**. First, compounds **17a**–**24a** were obtained in the reaction of 2-(chloroseleno)benzoyl chloride **16**, synthesized from anthranilic acid **15** according to our previously reported method [19], with appropriate primary amines, mostly commercially available. Only in the case of products **22a**–**24a** and **22b**–**24b** the substrate ester amines had to be synthesized from corresponding carboxylic acids by the treatment with thionyl chloride and ethyl iodide. Next, the obtained benzisoselenazolones **17a**–**24a** were transformed to diselenides **17b**–**24b** by a sodium borohydride reduction and air oxidation method. All obtained compounds were synthesized in good yields (45–93%) [21] (Scheme 1).

**Scheme 2:**
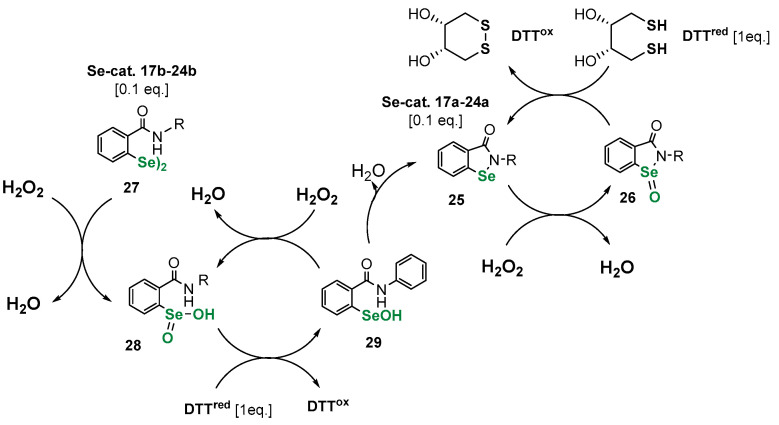
Mechanism of H_2_O_2_ reduction by benzisoselenazolones **17a**–**24a** and diselenides **17b**–**24b**.

The antioxidant activity of all Se-derivatives was evaluated by two assays. First, by the procedure based on the oxidation of dithiol to disulfide presented by Iwaoka and co-workers [29]. The evaluation starts from the reduction of hydrogen peroxide to water with simultaneous activation of the pre-catalyst: benzisoselenazolone **25** to selenooxide **26** or diselenide **27** to seleninic acid **28**. The activated compounds **26** or **28** further transform the dithiol DTT^red^ to the disulfide DTT^ox^. Catalyst **28** can be additionally regenerated from compound **29** by reducing an additional H_2_O_2_ molecule (Scheme 2). The progress of the process is monitored by ^1^H NMR spectra, recorded in specific time intervals. The results collected for each Se-catalyst **17a**–**24a** or **17b**–**24b** are presented in Table 1. 

In general, diselenides **17b**–**24b** exhibited better-H_2_O_2_-scavenging properties, revealing that the cleavage of the Se-Se bond and oxidation to seleninic acid **28** is a rapid process that speeds up the hydrogen peroxide reduction. Compounds possessing an ester moiety presented increased activity. In the case of diselenides **22b**–**24b**, the more elongated the carbon-chain linker, the more efficient the catalyst. For corresponding benzisoselenazolones, the optimal chain length was seven carbon atoms, with the best result obtained for compound **23a**. Surprisingly, for *N*-alkyl derivatives, elongation of the hydrocarbon moiety did not improve the activity. Compounds **19a** and **19b** were, respectively, 1,3-times and 4 times less active than their scaled-down equivalents **18a** and **17b**.

Next, the antioxidant activity was further evaluated by the ABTS (2,2′-azino-bis(3-ethylbenzothiazoline-6-sulfonic acid) assay. In this test, anti-radical activity has been assessed as the amount of antioxidant required to reduce the initial concentration of organic radical cation (ABTS^•+^) by 50% (EC_50_). ABTS assay occurs via a mixed mode reaction mechanism (SET and HAT) and allows the analysis of hydrophilic and lipophilic antioxidants with pH range 3–9 [30]. ABTS% assay determines the scavenging ability of antioxidant activity by reacting with a strong antioxidant agent (potassium persulfate) in the presence of ABTS salt. The percentage of inhibition in ABTS% assays are expressed using effective concentration (EC50) values, which are reported as the amount of antioxidants required to decrease the initial concentration of scavenging radicals by 50% [31].

**Figure 3:**
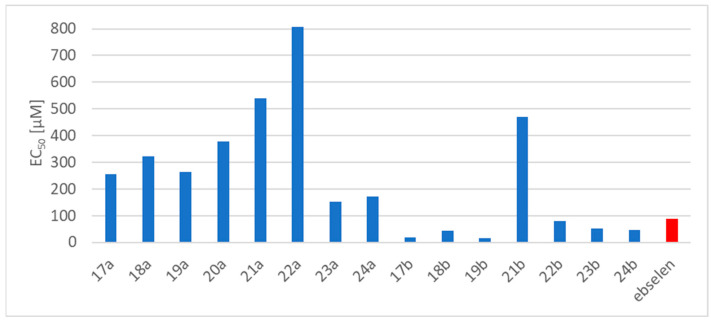
The EC_50_ values of the ABTS^•+^ radical scavenging activity of tested compounds.

In this study, ABTS assay was developed for the spectrophotometric determination of obtained compounds. The proposed method is based upon the ability of Se-derivatives to decolorize ABTS^•+^, which is blue-green in the solution and has an absorbance maximum of 734 nm. The obtained results for tested compounds are summarized in Table 2 and Figure 3.

The antioxidant activity of many molecules is mainly attributed to their redox properties, which allow them to act as reducing agents, hydrogen donors and quenchers of singlet oxygen. Moreover, the intensity of the decrease in the ABTS^.+^ absorbance, in the presence of antioxidants, depends on the reaction duration, intrinsic antioxidant activity, and sample concentration. Based on the obtained results, we can conclude that under our reaction conditions, all tested compounds were characterized by antioxidant properties; however, their ability to scavenge free radicals depends on their structural composition.

By comparing the obtained EC_50_ values, it is evident that the *N*-functionalized benzisoselenazol-3(2*H*)-ones **17a**–**24a** are less effective in quenching free radicals than ebselen, with high scavenging properties (87.5 μM). Surprisingly, the presence of an additional phenyl ring (**20a** and **21a**) did not improve their antioxidant properties. On the other hand, introducing an ester group into the structure of derivatives **22a**–**24a** increased their ability to scavenge free radicals. In this group of tested compounds, radical scavenging activity (RSA) values decrease in the following order: **22a** < **24a** < **23a**, which suggests that no dependence of RSA on the chain length is observed. One-way ANOVA, followed by Duncan’s multiple comparison tests, were performed to determine the significant differences between data and statistically significant differences for the determined RSA values for the tested compounds that were observed.

In the case of diselenides **17b**–**24b,** the values of RSA were better or on a comparable level compared to ebselen, except **21b** (Figure 3). The latter suggests that the presence of an additional phenyl ring does not improve the discussed properties of the obtained compounds and aligns with the results obtained for benzisoselenazolones **17a**–**24a**. 

Special attention should be paid to the group of *N*-alkyl diselenides **17b**–**19b** with the lowest EC 50 value. Such significant differences may be due to the presence of more than one selenium in the discussed compound structures. Moreover, the low bond energies of Se-Se (172 kJ mol^−1^) make the diselenide easy to be oxidized or reduced.

The **19b** (EC_50_ = 14.7 µM) had the highest activity, which indicated that this compound had significantly high efficacy in comparison to ebselen, which is commonly used in biological and biochemical applications to reduce oxidative stress or damage. What surprised us was that no dependence of RSA on chain length between **17b**–**19b** group was observed. In contrary, for ester diselenides **22b**–**24b** free radical scavenging capacity increased with chain elongation: from 79.5 µM to 46.9 µM, respectively. Compared to the previous group of compounds (**17b**–**19b**), the free radical scavenging properties are lower but higher than those obtained for Ebselen. As before, statistically significant differences for the determined RSA values for tested diphenyl diselenides were observed.

It should be noted that an unambiguous interpretation of the obtained results and comparison to the literature is difficult. For example, the rate of radical trapping and mechanism, the stoichiometry of the process, and the ratio of reagents should be considered. Consequently, one of disadvantages of ABTs procedure is time because the reaction may be different for slow and quick reactions. The ABTS^•+^ test is usually used to evaluate the antioxidant capacity of biological fluids and many natural compounds. In addition, the mechanism of the reaction between the synthetic cation radical and the antioxidant is discussed in the literature. Selected antioxidants can form adducts with ABTS^•+^, whereas others can undergo oxidation without coupling with ABTS^•+^. Several authors discuss possible mechanisms involved in ABTS^•+^ quenching, suggesting the mixed hydrogen atom transfer/single electron transfer (HAT/SET) reaction mechanism, stepwise electron transfer–proton transfer (ET-PT), and concerted electron–proton transfer (CEP) mechanism with water as the proton acceptor, among others, and apparently any of them can occur in parallel or prevail. The facts presented above show how many elements can affect the discussed RSA values. Moreover, establishing the structural features of tested compounds that determine the direction of interaction with ABTS^•+^ is important for understanding and interpretation of RSA measurements.

Finally, the antiproliferative activity of all derivatives **17a,b**–**24a,b** was evaluated towards breast cancer MCF-7 and leukemia HL-60 cell lines [32]. The highest cytotoxic potential was observed for benzisoselenazolone **20a** possessing an additional phenyl linker with IC_50_ values 10.0 ± 1.9 (MCF-7) and 5.4 ± 0.3 (HL-60) µM. Compound **20a** was also tested towards human umbilical vein endothelial cells (HUVEC), revealing a slightly higher inhibitory concentration needed to inhibit proliferation—being less toxic to normal than MCF-7 (1,1-fold) and HL-60 (2,1-fold) cell lines (Table 3). 

The presence of a heterocyclic isoselenazolone core and fixed Se-N bond in general enhances the cytotoxic potential in comparison to corresponding diphenyl diselenides. In the case of the *N*-alkyl derivatives **17a**–**19a**, we can observe that elongation of the carbon chain from C8 to C12 decreased the IC50 values toward both cancer cell lines. On the other hand, the incorporation of the aromatic ring, which additionally upgrades the bioactivity, is optimal, along with a shorter C10 group. Thus, the anticancer potential of compound **20a** is significantly higher than for derivative **21a**. For the corresponding *N*-alkyl diselenides **17b**–**19b**, changing the length of the carbon chain does not meaningfully modify the activity, and no relationship between the structure and IC50 value was observed towards both cancer cell lines. The presence of an additional phenyl ring in general enhanced the reactivity for compounds **20b** and **21b**. However, the results obtained for the MCF-7 cell line presented a decrease in the inhibitory concentration by five-times when the chain was elongated to 12 carbon atoms. In the case of ester-bearing benzisoselenazolones **22a**–**24a**, elongation of the carbon linker lowers the activity, in contrast to the results obtained in both antioxidant activity assays where the reactivity increases in the order **22a** < **24a** < **23a**. It can be observed that shorter distance between the reactive isoselenazolone core and the ester terminal group enhances the cytotoxic potential. In general, when the Se-N bond of the ester derivatives is reduced to a diselenide functionality, the anticancer potential of compounds **22b**–**24b** is significantly lower. It can be observed that incorporation of the polar ester functionality does not have a positive effect on the antiproliferative activity of the evaluated diselenides. However, in case of compound **24b** with the longest carbon linker, the transformation of the Se-N bond to a lower energy Se-Se bond decreases the IC50 value almost 10-times towards HL-60 cell line—from 118.7 ± 5.1 µM (benzisoselenazolone **24a**) to 12.8 ± 1.1 µM (diselenide **24b**). Comparing the result obtained for the most active *N*-4-(decyl)phenyl-1,2-benzisoselenazol-3(2*H*)-one **20a** to the reactivity of the well-known drug cisplatin, we can observe a similar activity towards HL-60 cell line but a 2,4-fold lower IC50 value of the newly designed derivative **20a**.

Taking into account the results collected in all activity assays, we can assume that the Se-Se bond with the lower energy reacts more rapidly with reactive oxygen species and is necessary for high antioxidant activity; the isoselenazolone core with a fixed Se-N bond is an essential pharmacophore for the Se-compound to express antiproliferative potential; installation of additional aromatic rings reduces the ROS-scavenging properties but improves the cytotoxic activity, probably due to more efficient receptor binding; substitution of the nitrogen atom with simple long carbon chains improves the antioxidant activity of *N*-functionalized *o*-amido diphenyl diselenides, which is likely to be associated with better solubility in organic solvents; the attachment of the ester group at the end of the *N*-carbon chains has an unclear effect—significantly enhancing the H_2_O_2_-reduction potential of diphenyl diselenides in the DTT assay, a moderate free radical scavenging capacity improvement in the ABTS assay (lower than for *N*-alkyl derivatives **17b**–**19b**, but higher than for ebselen) and in the cytotoxic activity evaluation a positive effect on the antiproliferative capacity only in case of shorter *N*-carbon linkers for both benzisoselenazolones and corresponding diselenides. 

## 3. Materials and Methods

### 3.1. General 

NMR spectra were recorded on Bruker Avance III/400 or Bruker Avance III/700 (Karlsruhe, Germany) for ^1^H and 176.1 MHz or 100.6 MHz for ^13^C (see Supplementary Material). Chemical shifts were recorded relative to SiMe_4_ (δ0.00) or solvent resonance (CDCl_3_ δ7.26, CD3OD δ3.31). Multiplicities were given as s (singlet), d (doublet), dd (double doublet), ddd (double double doublet), t (triplet), dt (double triplet), and m (multiplet). The ^77^Se NMR spectra were recorded on Bruker Avance III/400 or Bruker Avance III/700 with diphenyl diselenide as an external standard. NMR spectra were carried out using ACD/NMR Processor Academic Edition. Melting points were measured with a Büchi Tottoli SPM-20 heating unit (Büchi Labortechnik AG, Flawil, Switzerland) and were uncorrected. Elemental analyses were performed on a Vario MACRO CHN analyzer. Column chromatography was performed using Sigma Aldrich 60 Å (52–73 A) 63–200 µm silica gel (Merck, Darmstadt, Germany).

### 3.2. General Procedure

Benzisoselenazolones **17a**–**21a** and diselenides **17b**–**24b** were synthesized according to our previously presented protocol [19]. General procedure for the synthesis of derivatives **22**–**24a**: To a solution of the amino acid (2.5 mmol) in ethanol (10 mL), cooled to 0 °C, thionyl chloride (10 mmol) was slowly added. After stirring at reflux for 3 h, the reaction was cooled to room temperature, the solvent was evaporated and the crude product was used for the next step without further purification. Next, the crude ester was dissolved in DCM (10 mL), then triethylamine (10 mmol) and a solution of 2-(chloroseleno)benzoyl chloride (2.5 mmol) in DCM (5 mL), respectively, were slowly added at 0 °C. The reaction was stirred at room temperature for 12 h, poured on water, and extracted with DCM. The combined organic layers were washed with 10% solution of NaHCO_3_ (10 mL) and brine (10 mL), dried over anhydrous magnesium sulfate and evaporated. The crude product was purified by column chromatography (silica gel, DCM). Characterization of all obtained products is presented in the Supporting Information File [33,34].

### 3.3. Antioxidant Activity Assays

#### 3.3.1. DTT-Assay

To a solution of compounds **17a**–**24a** and **17b**–**24b** (0.015 mmol) and dithiothreitol DTT^red^ (0.15 mmol) in 1.1 mL of CD_3_OD, 30% H_2_O_2_ (0.15 mmol) was added. ^1^H NMR spectra were measured right after the addition of hydrogen peroxide and then in specific time intervals. The concentration of the substrate was determined according to the changes in the integration on the ^1^H NMR spectra [29].

#### 3.3.2. ABTS Method

The standard solutions of tested compounds were prepared in methanol, whereas ABTS and potassium persulfate were prepared in water. A stock of ABTS radical cation (ABTS^•+^) was prepared by the reaction of 7 mM ABTS solution with 2.45 mM potassium persulfate and incubated in the dark at 25 °C for 24 h. Next, 0.1 mL of ABTS radical cation solution were mixed with different value of sample solution and made up to the 10 mL by methanol. The absorption was measured after 10 min at 734 nm against methanol. All samples were evaluated in triplicate. The % ABTS radical scavenging activity was calculated using the following equation: ABTS radical scavenging (%) = [(Abs control − Abs samples)/Abs control] × 100, where Abs control is the absorbance of the control reaction and Abs sample is the absorbance of the tested compounds. The ABTS^•+^ scavenging capacity was expressed as the concentrations of tested compounds necessary to give a 50% reduction in the original absorbance (EC_50_) based on linear regression analysis. Ebselen was used as positive control solutions [31].

### 3.4. MTT Viability Assays

The MTT (3-(4,5-dimethyldiazol-2-yl)-2,5 diphenyl tetrazolium bromide) assay, which measures the activity of cellular dehydrogenases, was based on the method of Mosmann [32]. Briefly, cells were seeded into 96-well plates (about 1.5 × 10^4^ cells per well, in 100 µL) and then left to adhere and grow for 24 h. Subsequently, 100 µL of the tested compounds in the medium was added to a final concentration of 0–250 μM for 24 h, followed by the addition of 100 µL MTT, 3 mg/mL in PBS, for the next 3 h. After the incubation, the medium was removed. The remaining insoluble formazan crystals were dissolved in 100 µL DMSO. The absorbance of the blue formazan product was measured at 560 nm iMark Bio-Rad microplate reader (Hercules, CA, USA) and compared with the control (untreated cells). All experiments were performed three times in triplicate. The concentration of the tested compounds required to inhibit cell viability by 50% (IC_50_) was calculated using Microsoft Excel software 2002 (10.2614.2625) for semi-log curve fitting with linear regression analysis. 

## 4. Conclusions

Herein, we have presented a series of benzisoselenazolones and corresponding diselenides *N*-functionalized with solely long carbon chains or with additional polar elements: phenyl rings or ester groups. The antioxidant activity of the obtained Se-derivatives was evaluated by two assays: DTT-assay and ABTS method. In general, diphenyl diselenides exhibited higher antioxidant potential than corresponding benzisoselenazolones. Besides the Se-Se bond, highly reactive and crucial for the elevated radical scavenging capacity, the presence of the ester terminal element and an elongated carbon chain also substantially enhanced the efficiency of the catalyst with the best results evaluated for derivatives **23b**, **24b** (DTT method) and **19b** (ABTS method). At this moment, the obtained EC_50_ values allow us to propose that synthesized diphenyl diselenides are promising as active antioxidants. Following this thought, the present experimental section may hopefully contribute an effort towards the development of new organoselenium compounds as potential antioxidants agents. Additionally, all compounds were evaluated as anticancer agents towards breast cancer MCF-7 and leukemia HL-60 cell lines. Contrary to the antioxidant activity assays, benzisoselenazolones were more reactive, proving that the presence of the isoselenazolone core and the Se-N bond is valid. Compound **20a**, possessing an additional phenyl linker and a C10 carbon chain, exhibited the highest cytotoxic potential. The obtained results can be a preselection tool for organoselenium compounds intended to be studied for their potential bioactivity related to their radical-scavenging capacity.

## Figures and Tables

**Table 1:** Results of the antioxidant activity measurement.

	Remaining DTT^red^ [%]			
Se-Catalyst [0.1 eq.]	5 min	15 min	30 min	60 min
Benzisoselenazol-3(2*H*)-ones				
**17a**	86	79	71	61
**18a**	84	77	68	54
**19a**	83	78	74	71
**20a**	83	78	75	72
**21a**	88	88	87	87
**22a**	91	90	90	90
**23a**	38	10	0	0
**24a**	39	23	14	8
Diphenyl diselenides				
**17b**	0	0	0	0
**18b**	9	0	0	0
**19b**	79	57	38	25
**20b**	85	83	82	81
**21b**	93	93	92	90
**22b**	51	50	50	48
**23b**	0	0	0	0
**24b**	0	0	0	0
Ebselen	75	64	58	52

**Table 2:** Results collected from the ABTS radical scavenging assay.

Compound	EC_50_ [µM] ± SEM ^1^
Benzisoselenazol-3(2*H*)-ones	
**17a**	255.5 ^C^ ± 7.1
**18a**	322.1 ^D^ ± 11.8
**19a**	264.0 ^C^ ± 4.4
**20a**	378.0 ^E^ ± 12.5
**21a**	539.2 ^F^ ± 8.2
**22a**	807.7 ^G^ ± 3.7
**23a**	153.2 ^A^ ± 6.1
**24a**	172.1 ^B^ ± 9.5
Diphenyl diselenides	
**17b**	18.9 ^B^ ± 1.2
**18b**	44.7 ^C^ ± 2.1
**19b**	14.7 ^A^ ± 1.6
**21b**	470.1 ^G^ ± 7.5
**22b**	79.5 ^F^ ± 2.3
**23b**	51.5 ^E^ ± 1.9
**24b**	46.9 ^D^ ± 2.1
Ebselen	87.5 ± 0.8

^1^ Different letters (^A–G^) within the same column indicate significant differences (one-way ANOVA and Duncan test, *p* < 0.05); sorted from the lowest to highest values, where “A” was the lowest.

**Table 3:** The antiproliferative activity of compounds **17a**,**b**–**24a**,**b**.

	IC_50_ [µM] ± SEM		
Compound	MCF-7	HL-60	HUVEC
Benzisoselenazol-3(2*H*)-ones			
**17a**	89.2 ± 6.7	52.2 ± 1.9	-
**18a**	66.8 ± 5.3	79.0 ± 0.9	-
**19a**	19.8 ± 2.1	11.5 ± 0.7	-
**20a**	10.0 ± 1.9	5.4 ± 0.3	11.2 ± 1.1
**21a**	250.0 ± 8.0	48.0 ± 0.8	-
**22a**	15.2 ± 1.2	32.7 ± 0.3	-
**23a**	28.5 ± 2.1	52.2 ± 1.9	-
**24a**	28.7 ± 2.2	118.7 ± 5.1	-
Diphenyl diselenides			
**17b**	156.0 ± 11.0	158.0 ± 1.0	-
**18b**	153.0 ± 14.2	121.0 ± 8.2	-
**19b**	238.0 ± 16.5	154.0 ± 4.5	-
**20b**	200.0 ± 16.4	23.4 ± 0.4	-
**21b**	38.9 ± 4.2	70.6 ± 4.0	-
**22b**	308.0 ± 24.0	237.0 ± 21	-
**23b**	>400.0	>400.0	-
**24b**	152.0 ± 2.4	12.8 ± 1.1	-
Cisplatin	23.6 ± 1.3	4.9 ± 0.1	-

## Data Availability

Data are contained within the article and supplementary material.

## Supplementary Materials

The following supporting information can be downloaded at: https://www.mdpi.com/article/10.3390/ph16111560/s1, general procedure for the synthesis of compounds **17a**–**24a, 17b**–**24b**, NMR spectra of compounds **17a**–**24a, 17b**–**24b.**
