# Supplementary material for: The Influence of Long Carbon Chains on the Antioxidant and Anticancer Properties of N-Substituted Benzisoselenazolones and Corresponding Diselenides
Source: Pharmaceuticals (Basel). 2023 Nov 4;16(11):1560. doi: 10.3390/ph16111560 (PMC10675721; doi:10.3390/ph16111560)
Supplement: Supplementary file 1 [file pharmaceuticals-16-01560-s001.zip › Supporting Information.pdf]

Supporting Information

The Influence of Long Carbon Chains on the Antioxidant and Anticancer Properties of *N*-Substituted Benzisoselenazolones and Corresponding Diselenides

Agata J. Pacuła-Miszewska [1,*], Magdalena Obieziurska-Fabisiak [1], Aneta Jastrzębska [2], Angelika Długosz-Pokorska [3], Katarzyna Gach-Janczak [3] and Jacek Ścianowski [1]

[1] Department of Organic Chemistry, Faculty of Chemistry, Nicolaus Copernicus University, 7 Gagarin Street, 87-100 Torun, Poland
[2] Department of Analytical Chemistry and Applied Spectroscopy, Faculty of Chemistry, Nicolaus Copernicus University in Torun, 7 Gagarin Street, 87-100 Torun, Poland
[3] Department of Biomolecular Chemistry, Faculty of Medicine, Medical University of Lodz, Mazowiecka 6/8, 92-215 Lodz, Poland
* Correspondence: pacula@umk.pl

Index of content

I. General 1
II. Synthesis of benzisoselenazol-3(2H)-ones **17a**-**21a** 2
III. Synthesis of ester derivatives **22**-**24a** 4
IV. Synthesis of diselenides **17b**-**24b** 5
V. NMR spectra of compounds **17a**-**24a and 17b**-**24b** 9
VI. References 57

I. General

^{1}H NMR spectra were obtained at 300, 400 or 700 MHz and chemical shifts were recorded relative to $SiMe_4$ (δ0.00) or solvent resonance ($CDCl_3$ δ7.26, CD_3OD δ3.31). Multiplicities were given as: s (singlet), d (doublet), dd (double doublet), t (triplet), dt (double triplet), td (triplet double) and m (multiplet). The number of protons (n) for a given resonance was indicated by nH. Coupling constants were reported as a *J* value in Hz. NMR spectra were carried out using ACD/NMR Processor Academic Edition. Commercially available solvents DMF, DCM and MeOH (Aldrich) and chemicals were used without further purification. Column chromatography was performed using Merck 40-63D 60Å silica gel (Merck, Darmstadt, Germany).

II. Synthesis of benzisoselenazol-3(2*H*)-ones 17a-21a

To a solution of amine (2.0 mmol) and triethylamine (4.0 mmol) in dichloromethane (10 ml) 2-(chloroseleno)benzoyl chloride (2.0 mmol) was added. The mixture was stirred for 24h at room temperature, poured on water and extracted with DCM. The combined organic layers were dried over anhydrous magnesium sulfate and evaporated. The crude product was purified by column chromatography (silica gel, dichlorometane).

N-Octyl-1,2-benzisoselenazol-3(2*H*)-one 17a [1]

Yield: 79%; mp 68-70°C;
^{1}H NMR (700 MHz, $CDCl_3$) δ = 8.07 (dt, *J*=8.4, 0.7 Hz, $1H_{ar}$), 7.65 (dt, *J*=7.7 Hz, 0.7 Hz, $1H_{ar}$), 7.60 (td, *J*= 8.4, 1.4 Hz, $1H_{ar}$), 7.44 (td, *J*= 8.4, 1.4 Hz, $1H_{ar}$), 3.88 (t, *J*= 7.0 Hz, 2H), 1.77-1.73 (m, 2H), 1.44-1.40 (m, 2H), 1.38-1.34 (m, 2H), 1.33-1.26 (m, 6H), 0.89 (t, *J*= 7.0 Hz, 3H) ppm; ^{13}C NMR (100.62 MHz, $CDCl_3$) δ = 14.06 (CH_3), 22.62 (CH_2), 26.64 (CH_2), 29.14 (CH_2), 29.23 (CH_2), 30.59 (CH_2), 31.76 (CH_2), 44.89 (CH_2), 123.86 (CH_{ar}), 126.15 (CH_{ar}), 127.69 (C_{ar}), 128.83 (CH_{ar}), 131.81 (CH_{ar}), 137.66 (C_{ar}), 167.13 (C=O) ppm; ^{77}Se (76.35 MHz, $CDCl_3$), δ = 882.83 ppm; IR: 3092, 3056, 2954, 2919, 2848, 1625, 1588, 1558, 1457, 1442, 1377, 1348, 1309, 1249, 1219, 1187, 1155, 1113, 1076, 1054, 1024 cm^{-1}; Elemental Anal. Calcd for $C_{15}H_{21}NOSe$ (311.08): C, 58.06; H, 6.82, Found: C, 58.18; H, 6.87.

N-Decyl-1,2-benzisoselenazol-3(2*H*)-one 18a [2]

Yield: 87%; mp 65-67°C;
^{1}H NMR (700 MHz, $CDCl_3$) δ = 8.06 (dd, *J*=8.4, 1.4 Hz, $1H_{ar}$), 7.65 (dt, *J*=7.7, 0.7 Hz, $1H_{ar}$), 7.60 (td, *J*= 7.7, 1.4 Hz, $1H_{ar}$), 7.44 (td, *J*= 8.4, 1.4 Hz, $1H_{ar}$), 3.88 (t, *J*= 7.0 Hz, 2H), 1.77-1.72 (m, 2H), 1.44-1.39 (m, 2H), 1.38-1.34 (m, 2H), 1.32-1.28 (m, 10H), 0.90 (t, *J*= 7.0 Hz, 3H) ppm; ^{13}C NMR (100.62 MHz, $CDCl_3$) δ = 14.09 (CH_3), 22.66 (CH_2), 29.27 (CH_2), 29.47 (2xCH_2), 29.51 (2xCH_2), 30.59 (CH_2), 31.87 (CH_2), 44.89 (CH_2), 124.00 (CH_{ar}), 126.14 (CH_{ar}), 127.69 (C_{ar}), 128.82 (CH_{ar}), 131.80 (CH_{ar}), 137.67 (C_{ar}), 167.12 (C=O) ppm; ^{77}Se (76.35 MHz, $CDCl_3$), δ = 882.79 ppm; IR: 3092, 3057, 2953, 2918, 2971, 2848, 1625, 1588, 1558, 1457, 1442, 1377, 1349, 1309, 1249, 1189, 1158, 1115, 1054, 1024 cm^{-1}; Elemental Anal. Calcd for $C_{17}H_{25}NOSe$ (339.11): C, 60.35; H, 7.45, Found: C, 60.47; H, 7.39.

N-Dodecyl-1,2-benzisoselenazol-3(2H)-one 19a [3]

Yield: 45%; mp 71-72°C;
^{1}H NMR (700 MHz, $CDCl_3$) δ = 8.07 (dd, *J*=8.4, 1.4 Hz, 1H_{ar}), 7.64 (dt, *J*=7.7, 1.4 Hz, 1H_{ar}), 7.60 (td, *J*= 7.7, 1.4 Hz, 1H_{ar}), 7.44 (td, *J*= 7.7, 1.4 Hz, 1H_{ar}), 3.88 (t, *J*= 7.0 Hz, 2H), 1.77-1.72 (m, 2H), 1.44-1.39 (m, 2H), 1.37-1.34 (m, 2H), 1.32-1.27 (m, 14H), 0.90 (t, *J*= 7.0 Hz, 3H) ppm; ^{13}C NMR (100.62 MHz, $CDCl_3$) δ = 14.11 (CH_3), 22.68 (CH_2), 26.64 (CH_2), 29.27 (CH_2), 29.33 (CH_2), 29.48 (CH_2), 29.56 (CH_2), 29.61 (2xCH_2), 30.60 (CH_2), 31.91 (CH_2), 44.89 (CH_2), 123.92 (CH_{ar}), 126.16 (CH_{ar}), 127.73 (C_{ar}), 128.82 (CH_{ar}), 131.81 (CH_{ar}), 137.65 (C_{ar}), 167.19 (C=O) ppm; ^{77}Se (76.35 MHz, $CDCl_3$), δ = 882.90 ppm; IR: 2954, 2916, 2846, 1590, 1561, 1457, 1444, 1360, 1310, 1254, 1238, 1211, 1193, 1176, 1158, 1119, 1056, 1039, 1026 cm^{-1}; Elemental Anal. Calcd for $C_{19}H_{29}NOSe$ (367.14): C, 62.28; H, 7.98, Found: C, 62.39; H, 7.89.

N-4-(Decyl)phenyl-1,2-benzisoselenazol-3(2H)-one 20a

Yield: 37%; mp 130-132°C;
^{1}H NMR (400 MHz, $CDCl_3$) δ = 8.11 (dt, *J*=8.0, 1.2 Hz, 1H_{ar}), 7.66-7.64 (m, 2H_{ar}), 7.52-7.49 (m, 2H_{ar}), 7.48-7.45 (m, 1H_{ar}), 7.24 (dt, *J*=8.8, 2.0 Hz, 2H_{ar}), 2.62 (t, *J*= 8.0 Hz, 2H), 1.66-1.59 (m, 2H), 1.32-1.26 (m, 14H), 0.88 (t, *J*= 7.2 Hz, 3H) ppm; ^{13}C NMR (100.62 MHz, $CDCl_3$) δ = 14.11 (CH_3), 22.69 (CH_2), 29.30 (CH_2), 29.34 (CH_2), 29.51 (CH_2), 29.60 (CH_2), 29.67 (CH_2), 31.38 (CH_2), 31.91 (CH_2), 35.54 (CH_2), 123.70 (CH_{ar}), 125.40 (2xCH_{ar}), 126.49 (CH_{ar}), 127.60 (C_{ar}), 129.25 (2xCH_{ar}), 129.39 (CH_{ar}), 132.41 (CH_{ar}), 136.57 (C_{ar}), 137.69 (C_{ar}), 141.89 (C_{ar}), 165.71 (C=O) ppm; ^{77}Se (76.35 MHz, $CDCl_3$), δ = 959.23 ppm; IR: 3308, 2957, 2920, 2850, 1636, 1612, 1594, 1514, 1455, 1429, 1408, 1320, 1269, 1255, 1119, 1025 cm^{-1}; Elemental Anal. Calcd for $C_{23}H_{29}NOSe$ (415.14): C, 66.66; H, 7.05, Found: C, 66.53; H, 7.13.

N-4-(Dodecyl)phenyl-1,2-benzisoselenazol-3(2H)-one 21a

Yield: 31%; mp 136-138°C;
^{1}H NMR (400 MHz, $CDCl_3$) δ = 8.11 (d, *J*=7.6 Hz, 1H_{ar}), 7.66-7.64 (m, 2H_{ar}), 7.52-7.49 (m, 2H_{ar}), 7.48-7.45 (m, 1H_{ar}), 7.22 (d, *J*=8.4 Hz, 2H_{ar}), 2.62 (t, *J*= 7.6 Hz, 2H), 1.64-1.59 (m, 2H), 1.32-1.26 (m, 18H), 0.88 (t, *J*= 6.8 Hz, 3H) ppm; ^{13}C NMR (100.62 MHz, $CDCl_3$) δ = 14.11 (CH_3), 22.69 (CH_2), 29.30 (CH_2), 29.33 (CH_2), 29.50 (CH_2), 29.59 (CH_2), 29.63 (CH_2), 29.67 (CH_2), 31.38 (CH_2), 31.91 (CH_2), 31.92 (CH_2), 35.54 (CH_2), 123.68 (CH_{ar}), 125.40 (2xCH_{ar}), 126.49 (CH_{ar}), 127.60 (C_{ar}), 129.25 (2xCH_{ar}), 129.39 (CH_{ar}), 132.41 (CH_{ar}), 136.57 (C_{ar}), 137.69 (C_{ar}), 141.89 (C_{ar}), 165.71 (C=O) ppm; ^{77}Se (76.35 MHz, $CDCl_3$), δ = 958.95 ppm; IR: 2954,

2917, 2850, 1593, 1562, 1507, 1468, 1444, 1418, 1336, 1316, 1308, 1262, 1136 cm^{-1}; Elemental Anal. Calcd for $C_{25}H_{33}NOSe$ (443.17): C, 67.86; H, 7.52, Found: C, 67.99; H, 7.45.

III. Synthesis of ester derivatives 22-24a

To a solution of the amino acid (2.5 mmol) in ethanol (10 mL), cooled to 0°C, thionyl chloride (10 mmol) was slowly added. After stirring at reflux for 3h, the reaction was cooled to room temperature, the solvent was evaporated and the crude product was used for the next step without further purification. Next, the crude ester was dissolved in DCM (10 mL), then triethylamine (10 mmol) and a solution of 2-(chloroseleno)benzoyl chloride (2.5 mmol) in DCM (5 mL), respectively, were slowly added at 0°C. The reaction was stirred at room temperature for 12h, poured on water, and extracted with DCM. The combined organic layers were washed with 10% solution of $NaHCO_3$ (10 ml) and brine (10 ml), dried over anhydrous magnesium sulfate and evaporated. The crude product was purified by column chromatography (silica gel, DCM).

Ethyl 6-(3-oxobenzo[d][1,2]selenazol-2(3H)-yl)hexanoate 22a

Yield: 93%; mp 58-59°C;
^{1}H NMR (400 MHz, DMSO) δ = 8.02 (d, J=8.0 Hz, $1H_{ar}$), 7.78 (dd, J=8.0, 1.2 Hz, $1H_{ar}$), 7.58 (td, J= 7.2, 1.2 Hz, $1H_{ar}$), 7.40 (td, J= 7.6, 1.2 Hz, $1H_{ar}$), 4.03-3.98 (m, 2H), 3.69 (t, J=7.2 Hz, 2H), 2.26 (t, J=7.6 Hz, 2H), 1.63-1.52 (m, 4H), 1.32-1.27 (m, 2H), 1.13 (t, J= 7.2 Hz, 3H) ppm; ^{13}C NMR (100.62 MHz, DMSO) δ = 14.56 (CH_3), 24.58 (CH_2), 26.00 (CH_2), 30.05 (CH_2), 33.85 (CH_2), 43.47 (CH_2), 60.11 (CH_2), 126.22 (CH_{ar}), 126.30 (CH_{ar}), 127.77 (CH_{ar}), 128.51 (C_{ar}), 131.85 (CH_{ar}), 139.53 (C_{ar}), 166.71 (C=O), 173.27 (C=O) ppm; ^{77}Se (76.35 MHz, $CDCl_3$), δ = 855.12 ppm; IR: 3089, 3055, 2984, 2942, 2912, 2848, 1733, 1588, 1559, 1477, 1456, 1442, 1417, 1371, 1348, 1321, 1308, 1288, 1252, 1237, 1175, 1111, 1101, 1069, 1020 cm^{-1}; Elemental Anal. Calcd for $C_{15}H_{19}NO_3Se$ (341.05): C, 52.95; H, 5.63, Found: C, 53.07; H, 5.69.

Ethyl 8-(3-oxobenzo[d][1,2]selenazol-2(3H)-yl)octanoate 23a

Yield: 90%; mp 73-75°C;
^{1}H NMR (400 MHz, DMSO) δ = 8.01 (d, J=8.0 Hz, $1H_{ar}$), 7.78 (d, J=6.8 Hz, $1H_{ar}$), 7.58 (t, J= 8.4 Hz, $1H_{ar}$), 7.40 (t, J= 8.0 Hz, $1H_{ar}$), 4.04-3.99 (m, 2H), 3.69 (t, J=7.2 Hz, 2H), 2.24 (t, J=7.6 Hz, 2H), 1.59-1.46 (m, 4H), 1.27-1.20 (m, 6H), 1.14 (t, J= 7.2 Hz, 3H) ppm; ^{13}C NMR (100.62 MHz, $CDCl_3$) δ = 14.26 (CH_3), 24.86 (CH_2), 26.42 (CH_2), 28.89 (CH_2), 28.95 (CH_2), 30.48 (CH_2), 34.30 (CH_2), 44.79 (CH_2), 60.18 (CH_2), 123.93 (CH_{ar}), 126.18 (CH_{ar}), 127.63 (C_{ar}), 128.85 (CH_{ar}), 131.85 (CH_{ar}), 137.61 (C_{ar}), 167.14 (C=O), 173.79 (C=O) ppm; ^{77}Se (76.35 MHz, $CDCl_3$), δ = 883.24 ppm; IR: 3331, 2928, 2853, 1730, 1611, 1585, 1537, 1458, 1441, 1371, 1316, 1282, 1257, 1156, 1095, 1026 cm^{-1}; Elemental Anal. Calcd for $C_{17}H_{23}NO_3Se$ (369.08): C, 55.44; H, 6.29, Found: C, 55.32; H, 6.35.

Ethyl 12-(3-oxobenzo[d][1,2]selenazol-2(3H)-yl)dodecanoate 24a

Yield: 91%; mp 65-67°C;

^{1}H NMR (400 MHz, DMSO) δ = 8.02 (d, *J*=8.0 Hz, 1H$_{ar}$), 7.77 (d, *J*=7.2 Hz, 1H$_{ar}$), 7.58 (td, *J*= 8.0, 1.2 Hz, 1H$_{ar}$), 7.40 (t, *J*= 7.2 Hz, 1H$_{ar}$), 4.04-3.99 (m, 2H), 3.69 (t, *J*=7.2 Hz, 2H), 2.23 (t, *J*=7.6 Hz, 2H), 1.60-1.46 (m, 4H), 1.27-1.20 (m, 14H), 1.15 (t, *J*= 7.2 Hz, 3H) ppm; ^{13}C NMR (100.62 MHz, $CDCl_3$) δ = 14.26 (CH_3), 24.98 (CH_2), 26.61 (CH_2), 29.13 (CH_2), 29.22 (2xCH_2), 29.38 (CH_2), 29.41 (CH_2), 29.45 (CH_2), 30.57 (CH_2), 34.40 (CH_2), 44.88 (CH_2), 60.15 (CH_2), 123.93 (CH$_{ar}$), 126.16 (CH$_{ar}$), 127.67 (C$_{ar}$), 128.83 (CH$_{ar}$), 131.63 (CH$_{ar}$), 137.65 (C$_{ar}$), 167.13 (C=O), 173.93 (C=O) ppm; ^{77}Se (76.35 MHz, $CDCl_3$), δ = 882.81 ppm; IR: 3309, 2921, 2851, 1730, 1622, 1584, 1530, 1456, 1374, 1309, 1280, 1260, 1233, 1203, 1173, 1114, 1026 cm^{-1}; Elemental Anal. Calcd for $C_{21}H_{31}NO_3Se$ (425.15): C, 59.43; H, 7.36, Found: C, 59.58; H, 7.45.

IV. Synthesis of diselenides 17b-24b

To a solution of benzisoselenazolone **17a**-**24a** (1.0 mmol) in methanol (10 ml) cooled to 0°C, sodium borohydride (1.0 mmol) was added and the mixture was stirred for 1h. Water (15ml) was added and the mixture was oxidized with air for 1h. Formed precipitate was filtered and dried in air.

2,2'-Diselenobis((*N*-octyl)benzamide) 17b [1]

Yield: 75%, mp 76-77°C;

^{1}H NMR (700 MHz, $CDCl_3$) δ = 7.88 (dd, *J*=8.4, 0.7 Hz, 1H$_{ar}$), 7.46 (dd, *J*=7.7, 1.4 Hz, 1H$_{ar}$), 7.28 (td, *J*=7.7, 0.7 Hz, 1H$_{ar}$), 7.22 (td, *J*=8.4, 1.4 Hz, 1H$_{ar}$), 6.16 (s, NH), 3.50-3.47 (m, 2H), 1.67-1.62 (m, 2H), 1.41-1.38 (m, 2H), 1.34-1.26 (m, 8H), 0.88 (t, *J*= 7.0 Hz, 3H) ppm; ^{13}C NMR (100.61 Hz, $CDCl_3$) δ = 14.03 (CH_3), 22.61 (CH_2), 27.01 (CH_2), 29.19 (CH_2), 29.26 (CH_2), 29.65 (CH_2), 31.78 (CH_2), 40.32 (CH_2), 126.06 (CH$_{ar}$), 126.48 (CH$_{ar}$), 131.49 (CH$_{ar}$), 131.59 (CH$_{ar}$), 132.95 (C$_{ar}$), 133.55 (C$_{ar}$), 168.16 (C=O) ppm; ^{77}Se (76.35 MHz, $CDCl_3$), δ = 451.29 ppm; IR: 3295, 3054, 2922, 2852, 1611, 1585, 1538, 1457, 1432, 1374, 1318, 1284, 1261, 1210, 1164, 1061, 1026 $^{-1}$; Elemental Anal. Calcd for $C_{30}H_{44}N_2O_2Se_2$ (624.17): C, 57.87; H, 7.12, Found: C, 57.95; H, 7.06.

2,2'-Diselenobis((*N*-decyl)benzamide) 18b

Yield: 61%, mp 115-116°C;

NMR (700 MHz, $CDCl_3$) δ = 7.88 (dd, J=8.4, 0.7 Hz, $1H_{ar}$), 7.46 (dd, J=7.7, 1.4 Hz, $1H_{ar}$), 7.27 (td, J=8.4, 1.4 Hz, $1H_{ar}$), 7.21 (td, J=7.7, 1.4 Hz, $1H_{ar}$), 6.16 (s, NH), 3.50-3.47 (m, 2H), 1.67-1.62 (m, 2H), 1.42-1.38 (m, 2H), 1.34-1.31 (m, 2H), 1.30-1.22 (m, 10H), 0.87 (t, J= 7.0 Hz, 3H) ppm; ^{13}C NMR (100.61 Hz, $CDCl_3$) δ = 14.05 (CH_3), 22.65 (CH_2), 27.01 (CH_2), 29.27 (CH_2), 29.29 (2xCH_2), 29.53 (CH_2), 29.64 (CH_2), 31.87 (CH_2), 40.32 (CH_2), 126.05 (CH_{ar}), 126.47 (CH_{ar}), 131.49 (CH_{ar}), 131.58 (CH_{ar}), 132.95 (C_{ar}), 133.55 (C_{ar}), 168.16 (C=O) ppm; ^{77}Se (76.35 MHz, $CDCl_3$), δ = 451.14 ppm; IR: 3301, 2954, 2920, 2851, 1625, 1614, 1585, 1535, 1468, 1457, 1422, 1376, 1310, 1282, 1259, 1158, 1025 cm^{-1}; Elemental Anal. Calcd for $C_{34}H_{52}N_2O_2Se_2$ (680.24): C, 60.17; H, 7.72, Found: C, 60.02; H, 7.80.

2,2'-Diselenobis((*N*-dodecyl)benzamide) 19b [3]

Yield: 68%, mp 120-122°C;

^1H NMR (700 MHz, $CDCl_3$) δ = 7.92 (dd, J=8.4, 0.7 Hz, $1H_{ar}$), 7.49 (dd, J=7.7, 1.4 Hz, $1H_{ar}$), 7.30 (td, J=8.4, 1.4 Hz, $1H_{ar}$), 7.25 (td, J=7.7, 1.4 Hz, $1H_{ar}$), 6.18 (s, NH), 3.53-3.50 (m, 2H), 1.70-1.65 (m, 2H), 1.45-1.41 (m, 2H), 1.39-1.35 (m, 2H), 1.32-1.28 (m, 14H), 0.90 (t, J= 7.0 Hz, 3H) ppm; ^{13}C NMR (75.48 Hz, $CDCl_3$) δ = 14.08 (CH_3), 22.65 (CH_2), 26.60 (CH_2), 26.99 (CH_2), 29.30 (CH_2), 29.53 (CH_2), 29.60 (2xCH_2), 30.55 (CH_2), 31.88 (CH_2), 40.29 (CH_2), 44.86 (CH_2), 126.02 (CH_{ar}), 126.46 (CH_{ar}), 131.40 (CH_{ar}), 131.60 (CH_{ar}), 132.94 (C_{ar}), 133.37 (C_{ar}), 168.14 (C=O) ppm; ^{77}Se (76.35 MHz, $CDCl_3$), δ = 450.52 ppm; IR: 3284, 2915, 2848, 1624, 1587, 1541, 1460, 1444, 1433, 1375, 1360, 1323, 1305, 1282, 1236, 1207, 1156, 1129, 1092, 1026 cm^{-1}; Elemental Anal. Calcd for $C_{38}H_{60}N_2O_2Se_2$ (736.30): C, 62.11; H, 8.23, Found: C, 62.29; H, 8.15.

2,2'-Diselenobis((4-(decyl)phenyl)benzamide) 20b

Yield: 78%; mp 198-200°C;
^1H NMR (700 MHz, DMSO) δ = 10.48 (s, NH), 7.93 (d, J=7.0 Hz, $1H_{ar}$), 7.79 (d, J=7.7, $1H_{ar}$), 7.67 (d, J= 8.4 Hz, $2H_{ar}$), 7.46-7.40 (m, $2H_{ar}$), 7.20 (d, J= 8.4 Hz, $2H_{ar}$), 2.60-2.55 (m, 2H), 1.58-1.56 (m, 2H), 1.28-1.24 (m, 14H), 0.86 (t, J= 7.0 Hz, 3H) ppm; ^{13}C NMR (100.62 MHz, DMSO) δ = 14.42 (CH_3), 22.55 (CH_2), 29.03 (CH_2), 29.15 (CH_2), 29.33 (CH_2), 29.45 (CH_2), 29.47 (CH_2), 31.48 (CH_2), 31.75 (CH_2), 35.07 (CH_2), 121.06 (2xCH_{ar}), 126.86 (CH_{ar}), 128.92 (2xCH_{ar}), 129.00 (CH_{ar}), 130.65 (CH_{ar}), 132.40 (C_{ar}), 132.44 (CH_{ar}), 134.37 (C_{ar}), 136.78 (C_{ar}), 138.68 (C_{ar}), 166.63 (C=O) ppm; ^{77}Se (76.35 MHz, DMSO), δ = 442.10 ppm; IR: 3308, 2956, 2920, 2850, 1636, 1612, 1594, 1515, 1456, 1430, 1409, 1377, 1320, 1269, 1255. 1026 cm^{-1}; Elemental Anal. Calcd for $C_{46}H_{60}N_2O_2Se_2$ (832.30): C, 66.49; H, 7.28, Found: C, 66.59; H, 7.19.

2,2'-Diselenobis((4-(dodecyl)phenyl)benzamide) 21b

Yield: 74%; mp 201-203°C;

^{1}H NMR (400 MHz, DMSO) δ = 10.45 (s, NH), 7.92 (d, J=8.0 Hz, $1H_{ar}$), 7.77 (d, J=7.6, $1H_{ar}$), 7.64 (d, J= 8.4 Hz, $2H_{ar}$), 7.44-7.37 (m, $2H_{ar}$), 7.18 (d, J= 8.4 Hz, $2H_{ar}$), 2.55-2.49 (m, 2H), 1.60-1.50 (m, 2H), 1.27-1.23 (m, 18H), 0.82 (t, J= 6.8 Hz, 3H) ppm; ^{13}C NMR (176.10 MHz, DMSO) δ = 14.41 (CH_3), 22.55 (CH_2), 29.03 (CH_2), 29.14 (CH_2), 29.32 (CH_2), 29.44 (CH_2), 29.47 ($2xCH_2$), 29.49 (CH_2), 31.47 (CH_2), 31.74 (CH_2), 35.07 (CH_2), 121.08 ($2xCH_{ar}$), 126.87 (CH_{ar}), 128.92 ($2xCH_{ar}$), 129.00 (CH_{ar}), 130.67 (CH_{ar}), 132.00 (C_{ar}), 132.44 (CH_{ar}), 134.40 (C_{ar}), 136.79 (C_{ar}), 138.69 (C_{ar}), 166.64 (C=O) ppm; ^{77}Se (133.60 MHz, DMSO), δ = 443.56.79 ppm; IR: 3321, 2956, 2920, 2849, 1638, 1594, 1562, 1515, 1463, 1430, 1408, 1319, 1256, 1093, 1047 1024 cm^{-1}; Elemental Anal. Calcd for $C_{50}H_{68}N_2O_2Se_2$ (888.36): C, 67.70; H, 7.73, Found: C, 67.85; H, 7.61.

Diethyl 6,6'-((2,2'-diselanediylbis(benzoyl))bis(azanediyl))dihexanoate 22b

Yield: 88%, mp 72-75°C;

^{1}H NMR (400 MHz, DMSO) δ = 8.70 (t, J=5.6 Hz, $1H_{ar}$), 7.75 (dd, J=7.6, 0.8 Hz, $1H_{ar}$), 7.66 (dd, J= 8.0, 1.2 Hz, $1H_{ar}$), 7.36 (td, J= 7.6, 0.8 Hz, $1H_{ar}$), 7.30 (td, J= 7.2, 1.2 Hz, $1H_{ar}$), 4.03-3.98 (m, 2H), 3.29-3.26 (m, 2H), 2.28 (t, J=8.0 Hz, 2H), 1.59-1.50 (m, 4H), 1.37-1.29 (m, 2H), 1.15 (t, J= 7.2 Hz, 3H) ppm; ^{13}C NMR (100.62 MHz, DMSO) δ = 14.59 (CH_3), 24.67 (CH_2), 26.35 (CH_2), 29.11 (CH_2), 33.94 (CH_2), 39.57 (CH_2), 60.12 (CH_2), 126.59 (CH_{ar}), 128.28 (CH_{ar}), 130.32 (CH_{ar}), 131.96 (C_{ar}), 132.33 (C_{ar}), 133.74 (CH_{ar}), 167.73 (C=O), 173.32 (C=O) ppm; ^{77}Se (76.35 MHz, $CDCl_3$), δ = 441.64 ppm; IR: 3375, 3283, 3083, 3053, 2930, 2866, 1728, 1707, 1626, 1609, 1584, 1533, 1456, 1430, 1373, 1351, 1315, 1281, 1259, 1226, 1179, 1157, 1097, 1026 cm^{-1}; Elemental Anal. Calcd for $C_{30}H_{40}N_2O_6Se_2$ (684.12): C, 52.79; H, 5.91, Found: C, 52.62; H, 5.99.

Diethyl 8,8'-((2,2'-diselanediylbis(benzoyl))bis(azanediyl))dioctanoate 23b

Yield: 85%, mp 70-72°C;

^{1}H NMR (400 MHz, DMSO) δ = 8.70 (t, J=5.6 Hz, $1H_{ar}$), 7.74 (dd, J=7.6, 1.2 Hz, $1H_{ar}$), 7.65 (dd, J= 8.0, 1.2 Hz, $1H_{ar}$), 7.34 (td, J= 8.0, 1.6 Hz, $1H_{ar}$), 7.29 (td, J= 7.2, 1.2 Hz, $1H_{ar}$), 4.04-3.99 (m, 2H), 3.29-3.24 (m, 2H), 2.24 (t, J=7.6 Hz, 2H), 1.59-1.47 (m, 4H), 1.27-1.20 (m, 8H), 1.14 (t, J= 7.2 Hz, 3H) ppm; ^{13}C NMR (100.62 MHz, DMSO) δ = 14.60 (CH_3), 24.88 (CH_2), 26.75 (CH_2), 28.87 ($2xCH_2$), 29.38 (CH_2), 33.97 (CH_2), 60.09 ($2xCH_2$), 126.59 (CH_{ar}), 128.27 (CH_{ar}), 130.32 (CH_{ar}), 131.93 (CH_{ar}), 132.32 (C_{ar}), 133.80 (C_{ar}), 167.73 (C=O), 173.36 (C=O) ppm; ^{77}Se (133.62 MHz, DMSO), δ = 441.54 ppm; IR: 3396, 3284, 3080, 2929, 2853, 1735, 1704, 1626, 1609, 1585, 1529, 1456, 1368, 1319, 1283, 1247, 1157, 1099, 1025 cm^{-1}; Elemental Anal. Calcd for $C_{34}H_{48}N_2O_6Se_2$ (740.18): C, 55.28; H, 6.55, Found: C, 55.37; H, 6.62.

Diethyl 12,12'-((2,2'-diselanediylbis(benzoyl))bis(azanediyl))didodecanoate 24b

Yield: 78%, mp 80-82°C;

^{1}H NMR (400 MHz, DMSO) δ = 8.68 (t, *J*=5.2 Hz, $1H_{ar}$), 7.74 (d, *J*=6.8 Hz, $1H_{ar}$), 7.66 (d, *J*= 7.6, $1H_{ar}$), 7.37-7.28 (m, $2H_{ar}$), 4.04-3.99 (m, 2H), 2.23 (t, *J*=7.6 Hz, 2H), 1.55-1.47 (m, 4H), 1.29-1.20 (m, 16H), 1.15 (t, *J*= 7.2 Hz, 3H) ppm; ^{13}C NMR (100.62 MHz, DMSO) δ = 14.58 (CH_3), 24.91 (2xCH_2), 26.89 (CH_2), 28.89 (CH_2), 29.13 (CH_2), 29.21 (CH_2), 29.37 (CH_2), 29.44 (2xCH_2), 33.96 (2xCH_2), 60.06 (CH_2), 126.56 (CH_{ar}), 128.23 (CH_{ar}), 130.31 (CH_{ar}), 131.88 (CH_{ar}), 132.33 (C_{ar}), 133.82 (C_{ar}), 167.71 (C=O), 173.33 (C=O) ppm; ^{77}Se (133.62 MHz, DMSO), δ = 442.53 ppm; IR: 3294, 3048, 2980, 2917, 2848, 1720, 1612, 1585, 1561, 1530, 1476, 1464, 1433, 1419, 1377, 1344, 1320, 1280, 1262, 1234, 1204, 1180, 1168, 1118, 1080, 1168, 1118, 1083, 1069, 1053, 1027 cm^{-1}; Elemental Anal. Calcd for $C_{42}H_{64}N_2O_6Se_2$ (852.31): C, 59.29; H, 7.58, Found: C, 59.34; H, 6.64.

V. NMR spectra

***N*-Octyl-1,2-benzisoselenazol-3(2*H*)-one 17a**

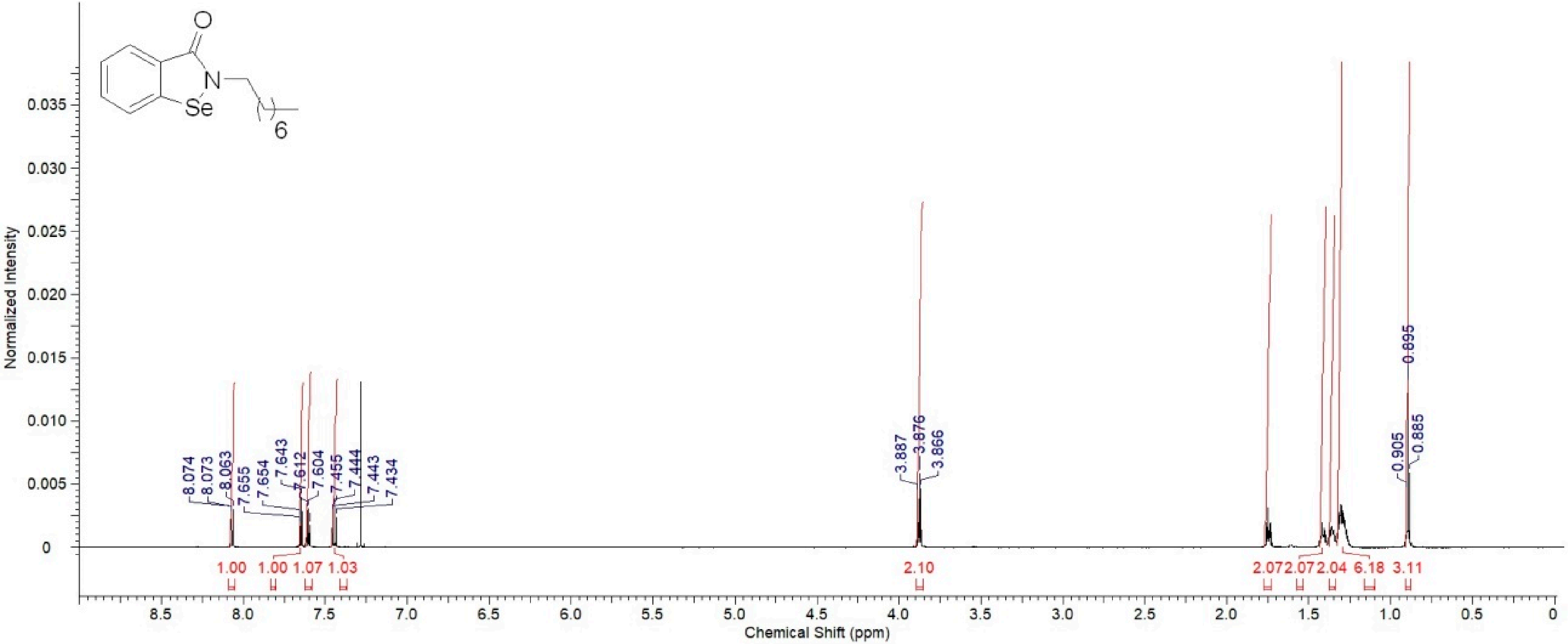

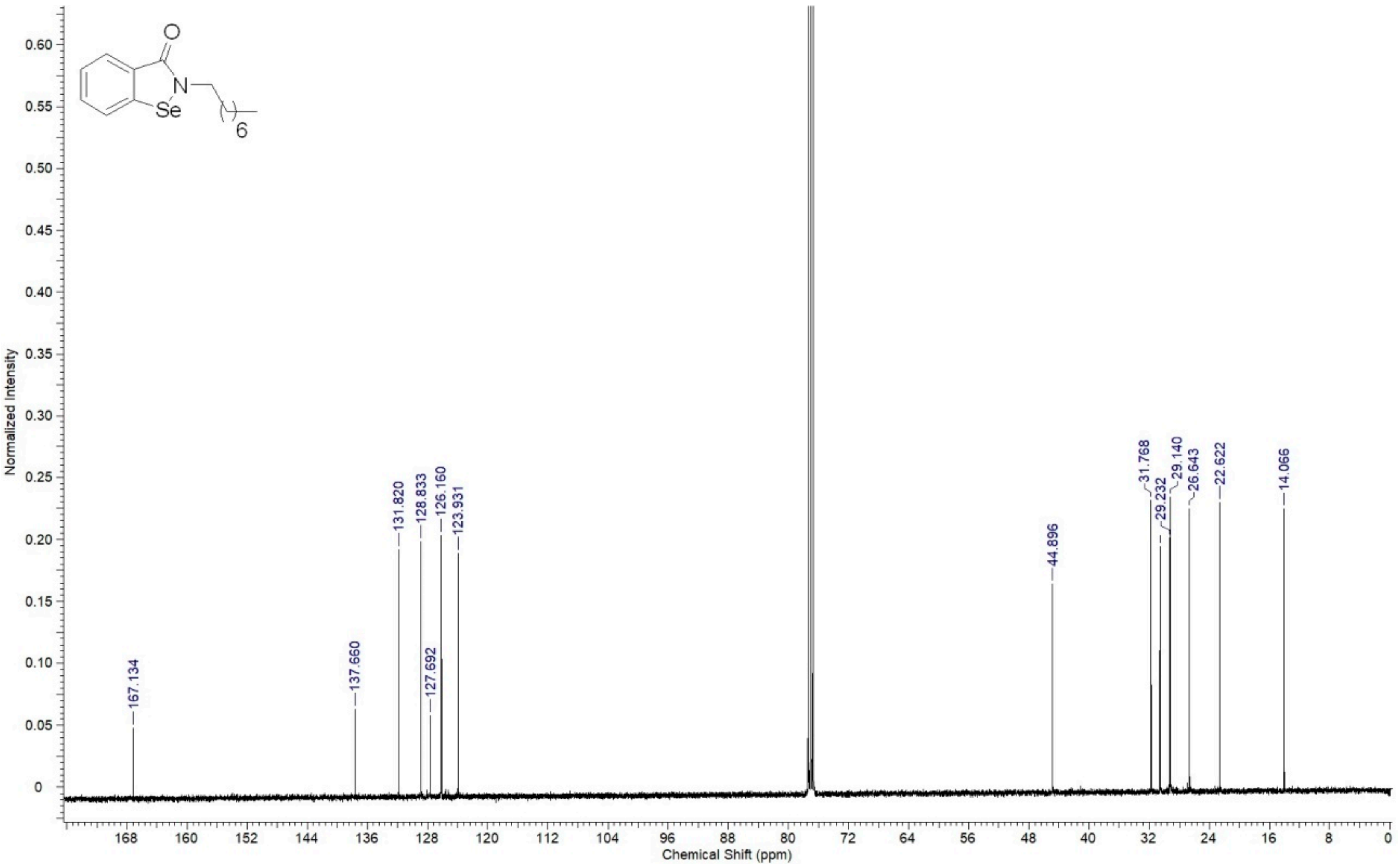

O
N
Se
6
Normalized Intensity
0.60
0.55
0.50
0.45
0.40
0.35
0.30
0.25
0.20
0.15
0.10
0.05
0
167.134
137.660
131.820
128.833
127.692
126.160
123.931
44.896
31.768
29.232
29.140
26.643
22.622
14.066
168
160
152
144
136
128
120
112
104
96
88
80
72
64
56
48
40
32
24
16
8
0
Chemical Shift (ppm)

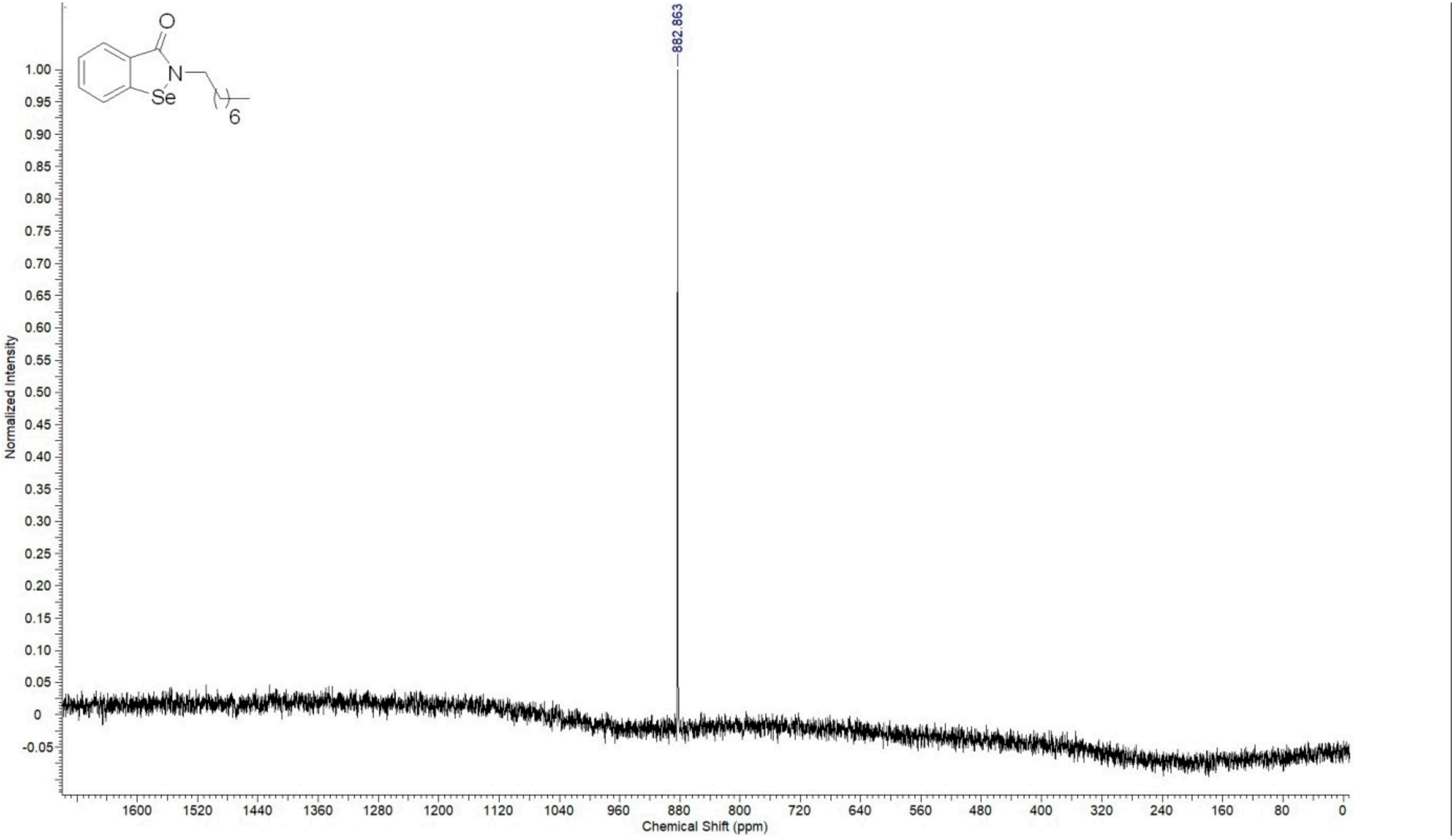
882.863
Normalized Intensity
Chemical Shift (ppm)
O
N
Se
6

***N*-Decyl-1,2-benzisoselenazol-3(2*H*)-one 18a**

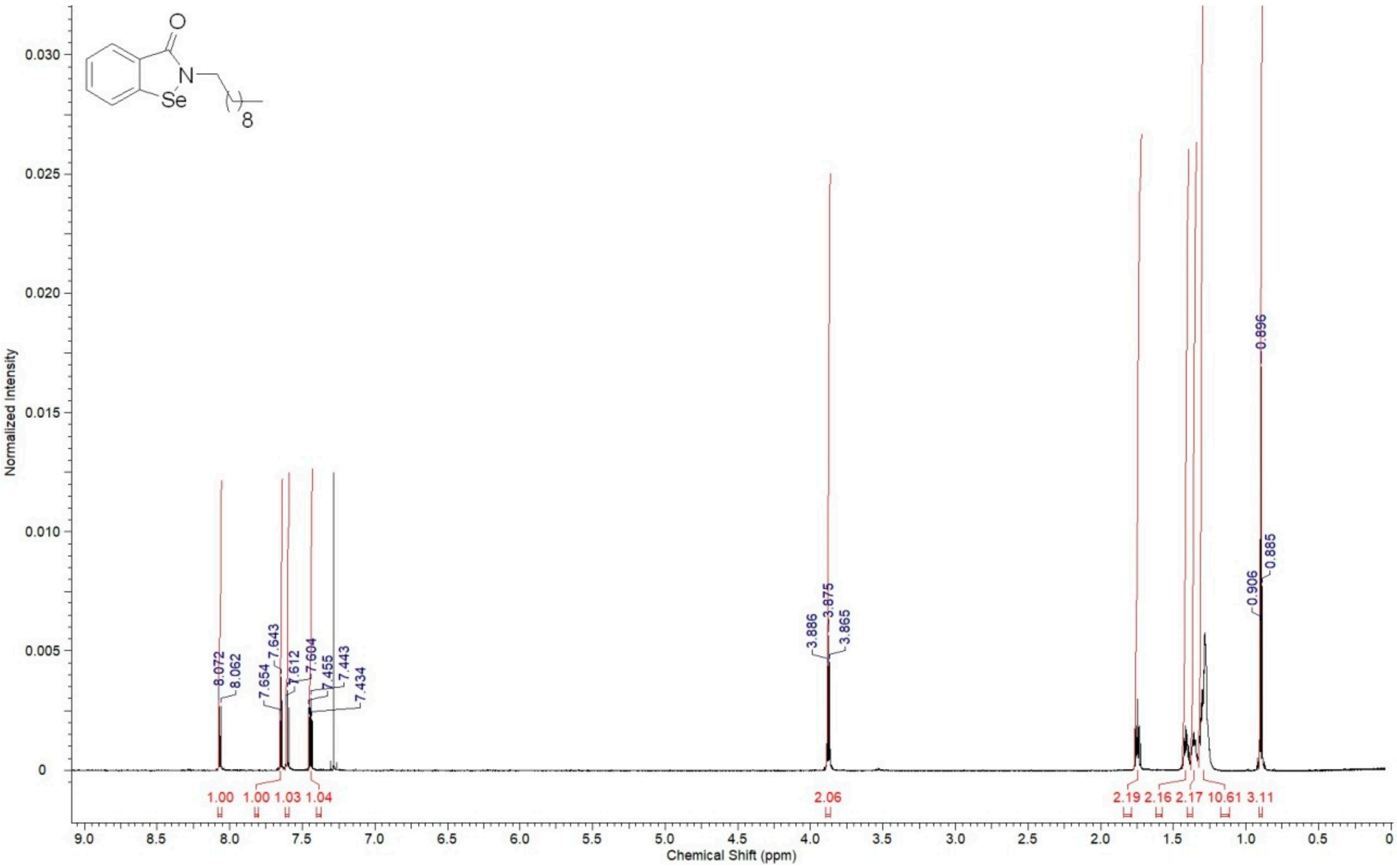

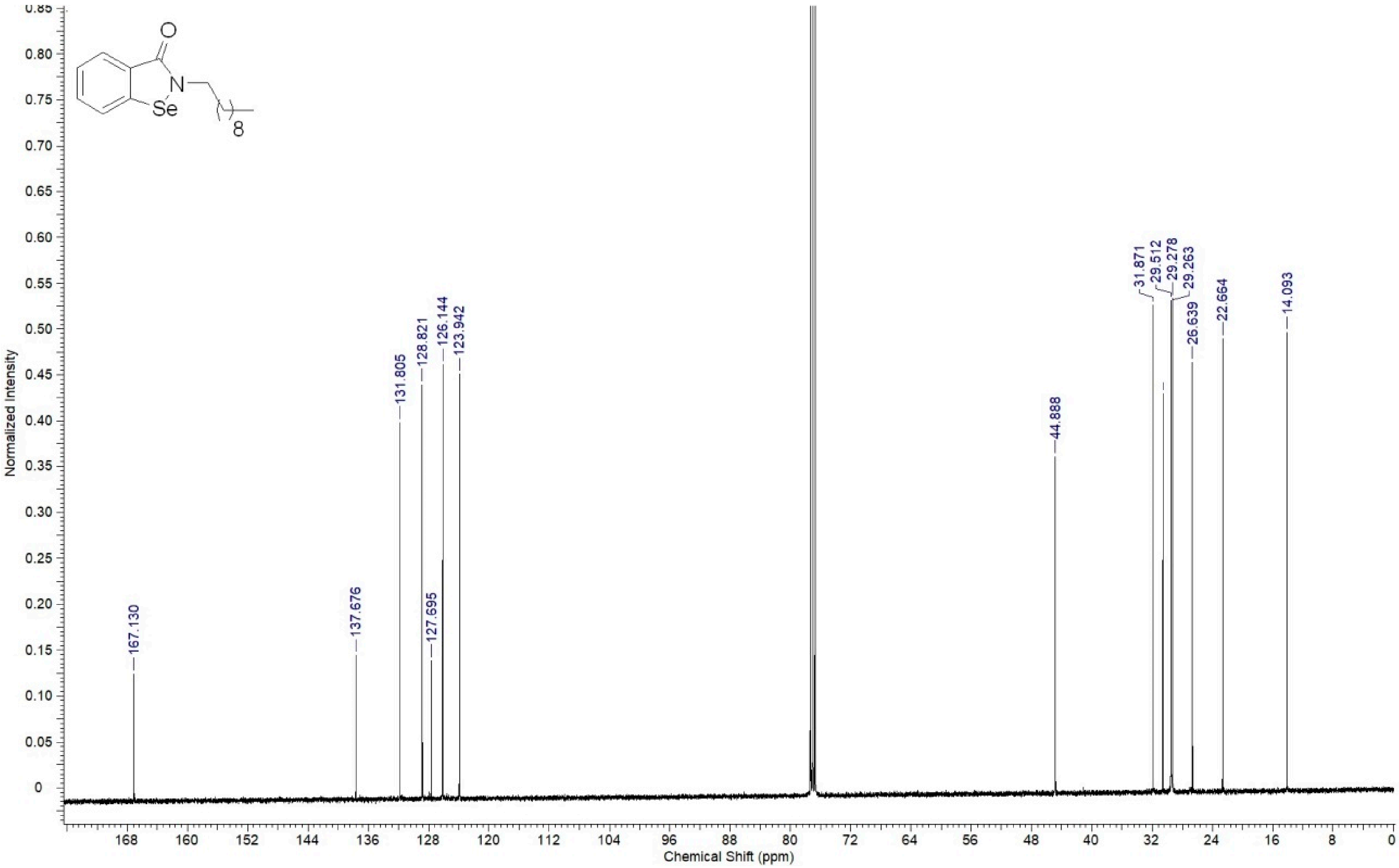
O
N
Se
8
167.130
137.676
131.805
128.821
127.695
126.144
123.942
44.888
31.871
29.512
29.278
29.263
26.639
22.664
14.093
Normalized Intensity
Chemical Shift (ppm)

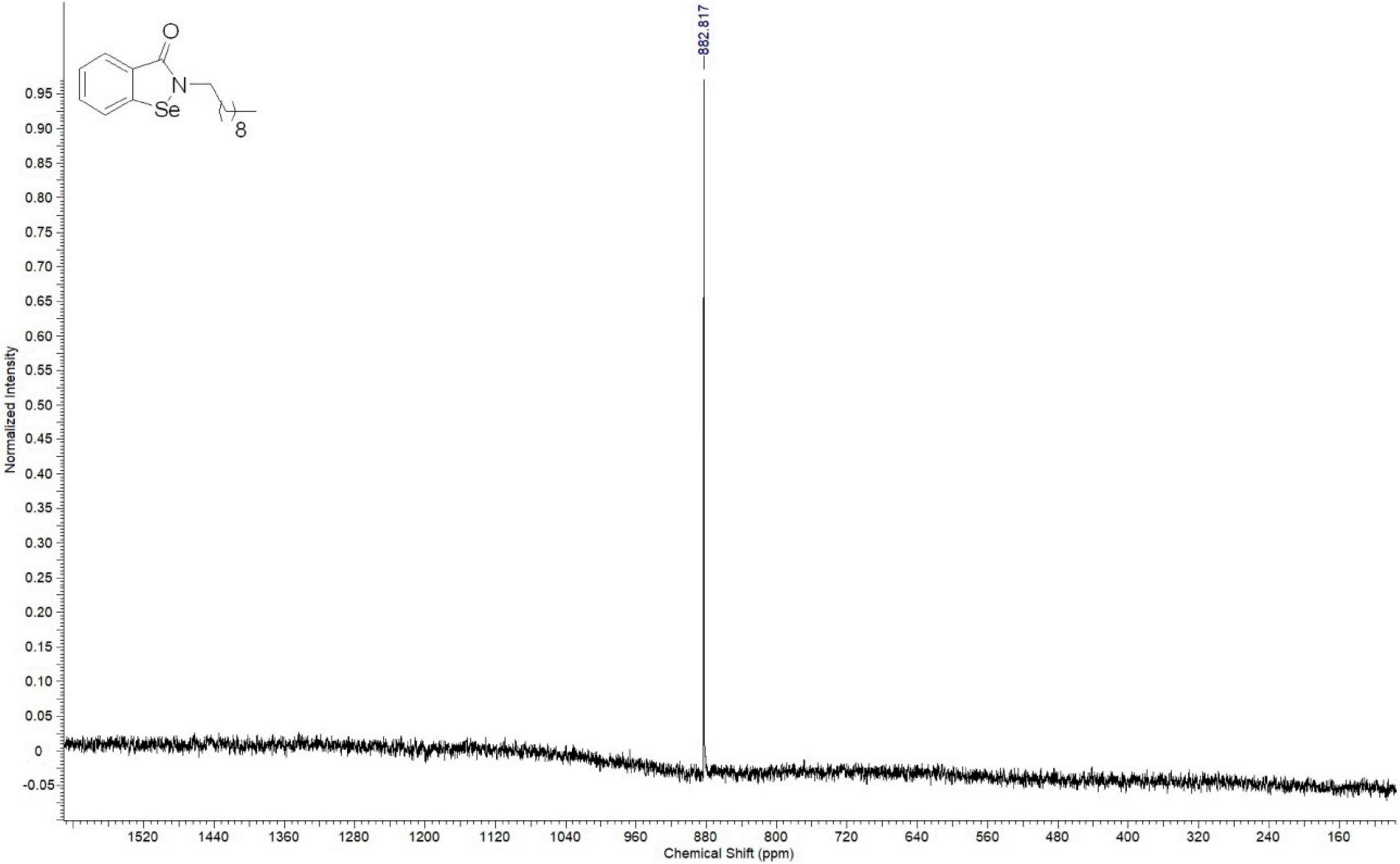
O
N
Se
8
882.817
Normalized Intensity
0.95
0.90
0.85
0.80
0.75
0.70
0.65
0.60
0.55
0.50
0.45
0.40
0.35
0.30
0.25
0.20
0.15
0.10
0.05
0
-0.05
1520
1440
1360
1280
1200
1120
1040
960
880
800
720
640
560
480
400
320
240
160
Chemical Shift (ppm)

N-Dodecyl-1,2-benzisoselenazol-3(2H)-one 19a

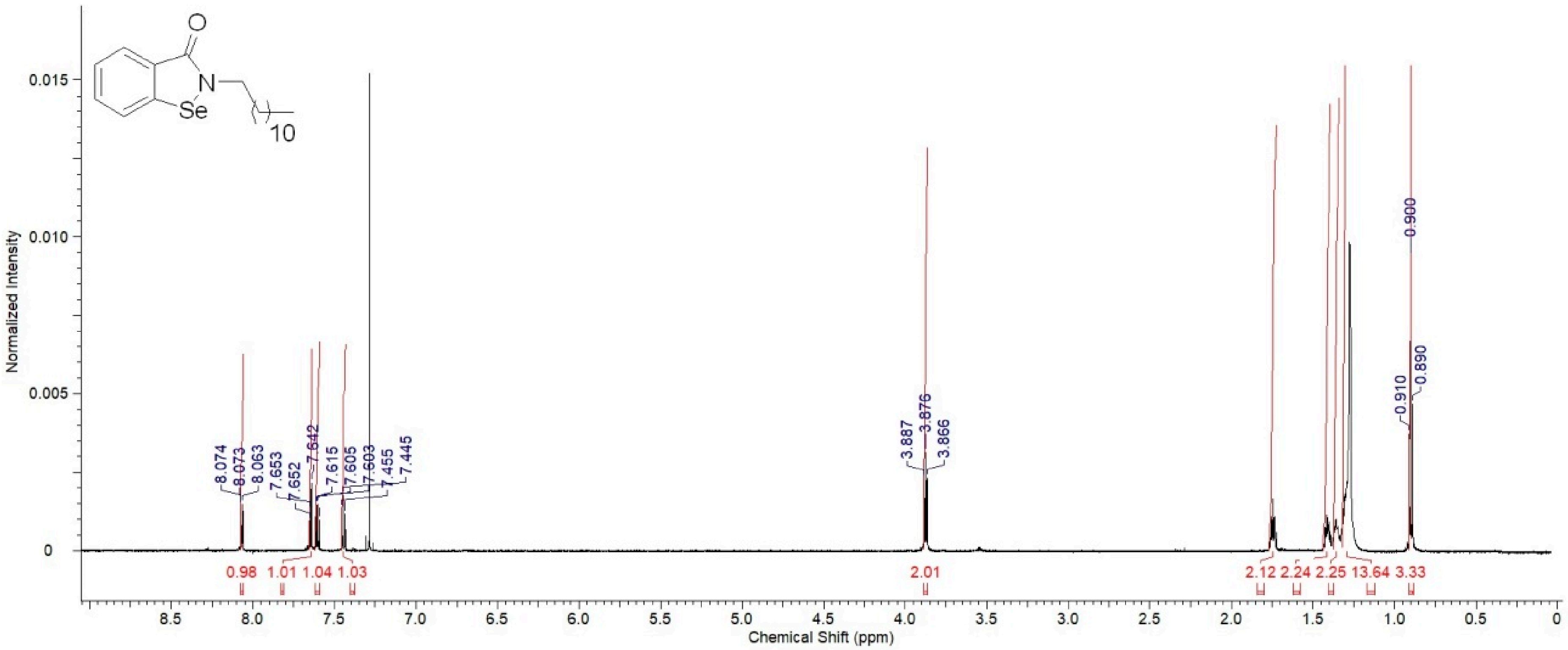

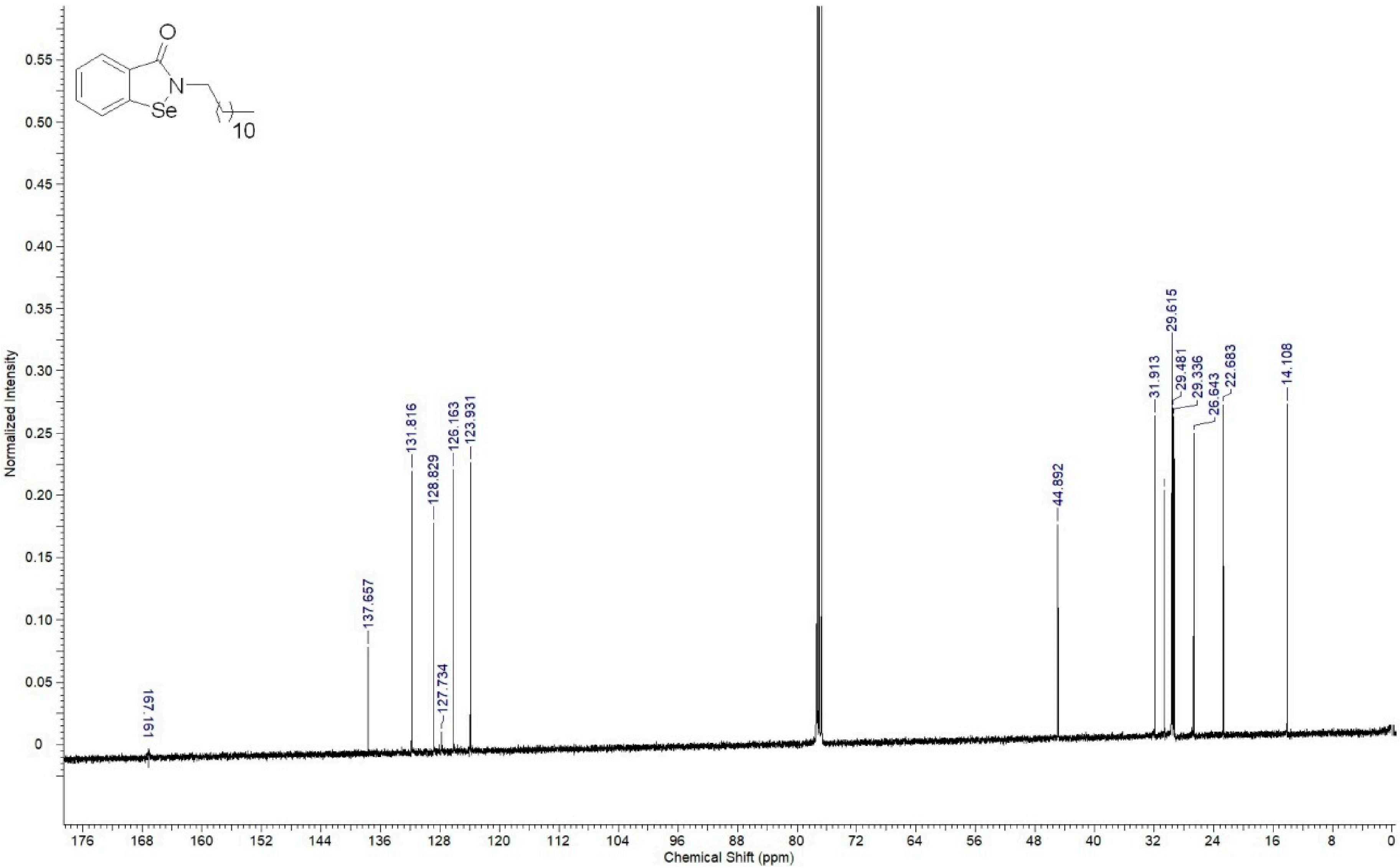
O
N
Se
10
Normalized Intensity
0.55
0.50
0.45
0.40
0.35
0.30
0.25
0.20
0.15
0.10
0.05
0
167.161
137.657
131.816
128.829
127.734
126.163
123.931
44.892
31.913
29.615
29.481
29.336
26.643
22.683
14.108
176
168
160
152
144
136
128
120
112
104
96
88
80
72
64
56
48
40
32
24
16
8
0
Chemical Shift (ppm)

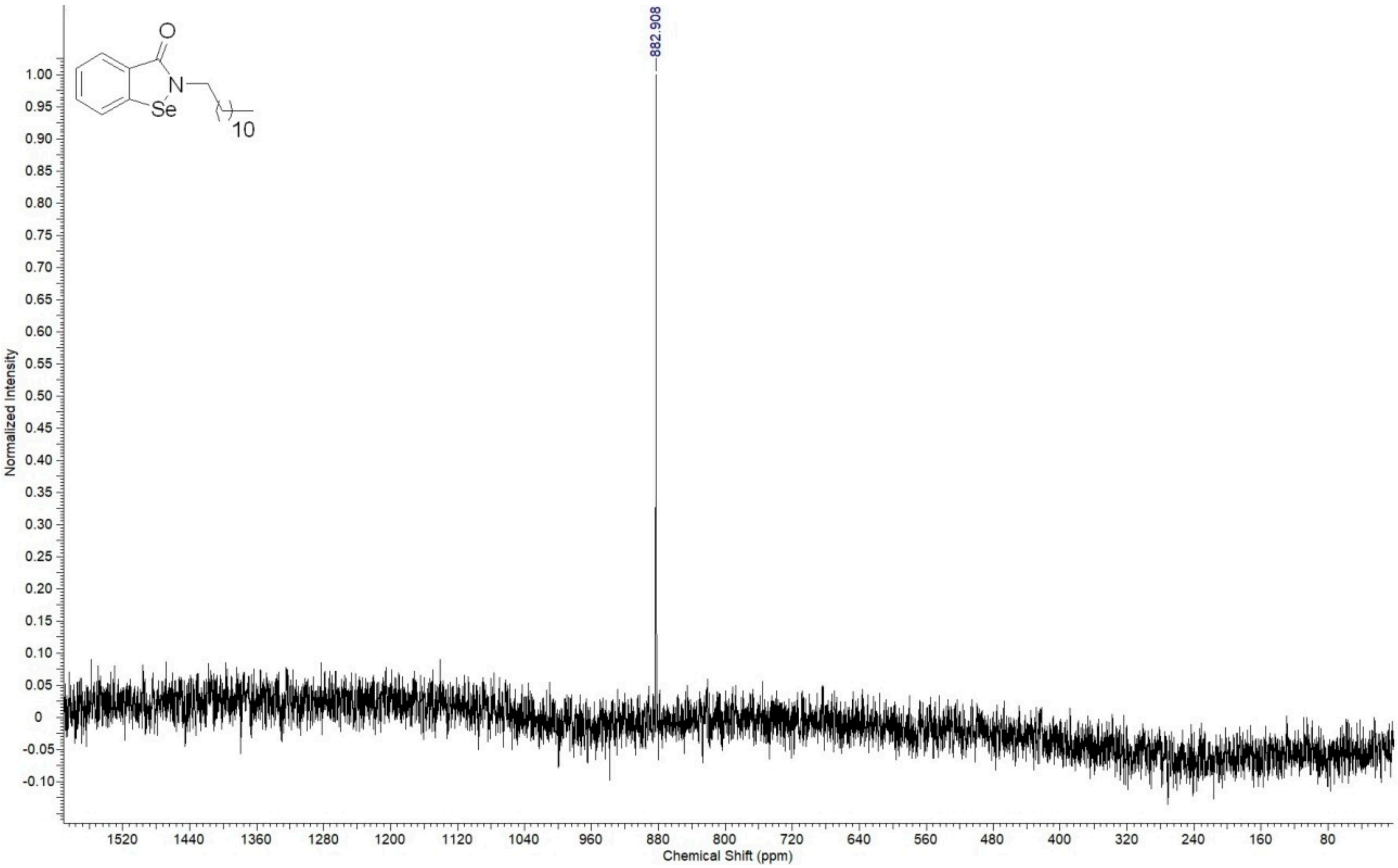

882.908
O
N
Se
10
Normalized Intensity
Chemical Shift (ppm)

***N*-4-(Decyl)phenyl-1,2-benzisoselenazol-3(2*H*)-one 20a**

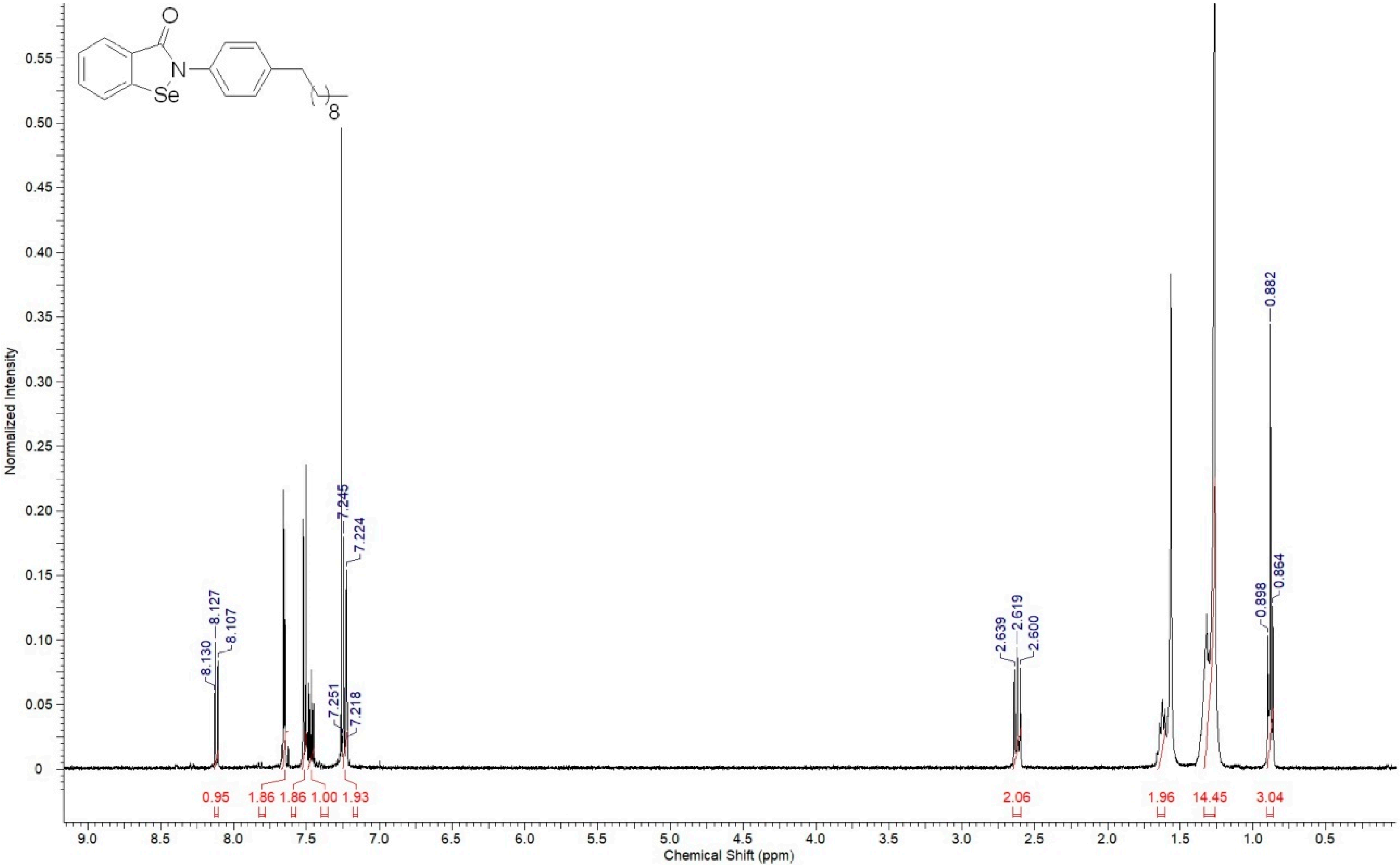

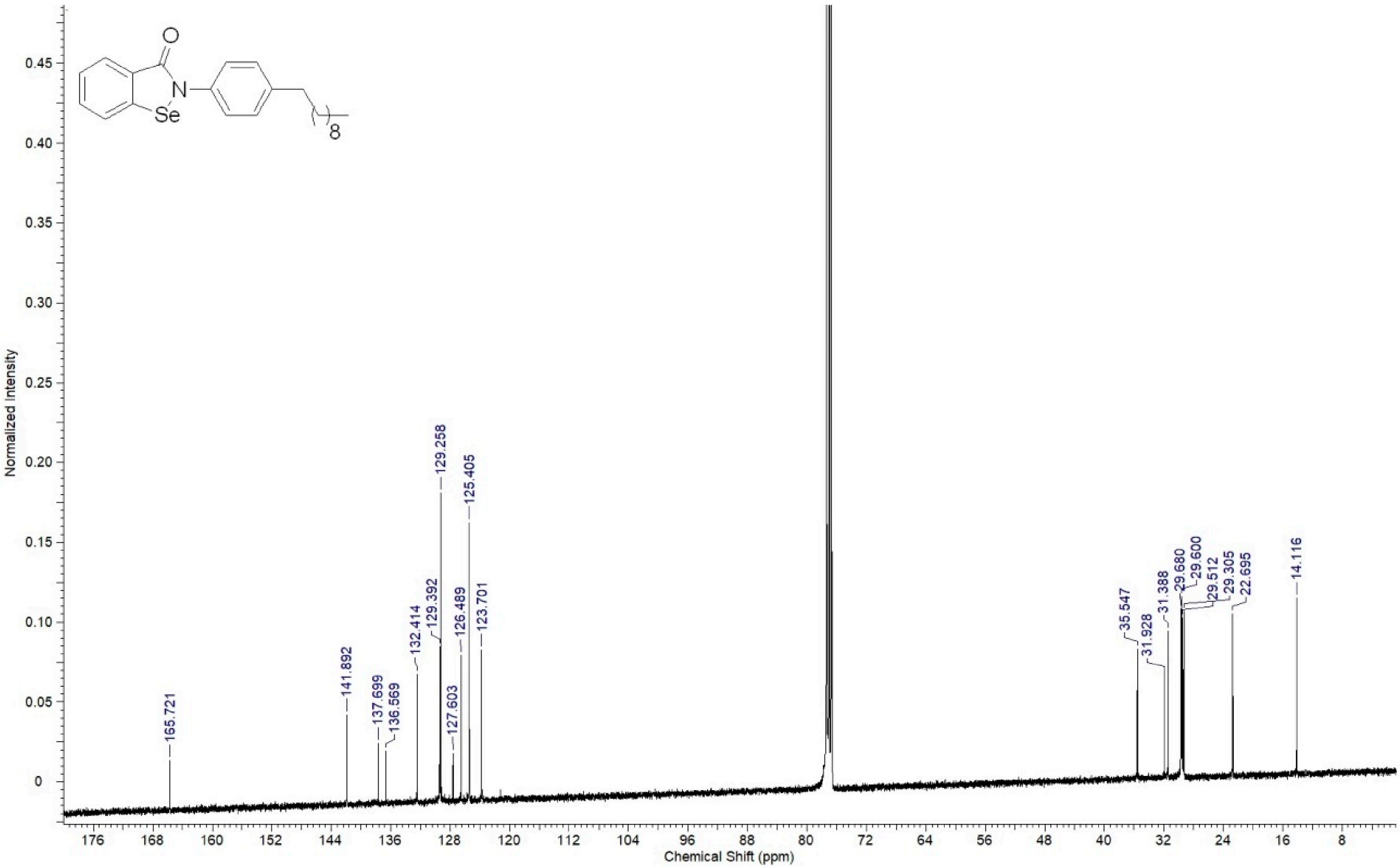
O
N
Se
8
Normalized Intensity
Chemical Shift (ppm)
165.721
141.892
137.699
136.569
132.414
129.392
129.258
127.603
126.489
125.405
123.701
35.547
31.928
31.388
29.680
29.600
29.512
29.305
22.695
14.116

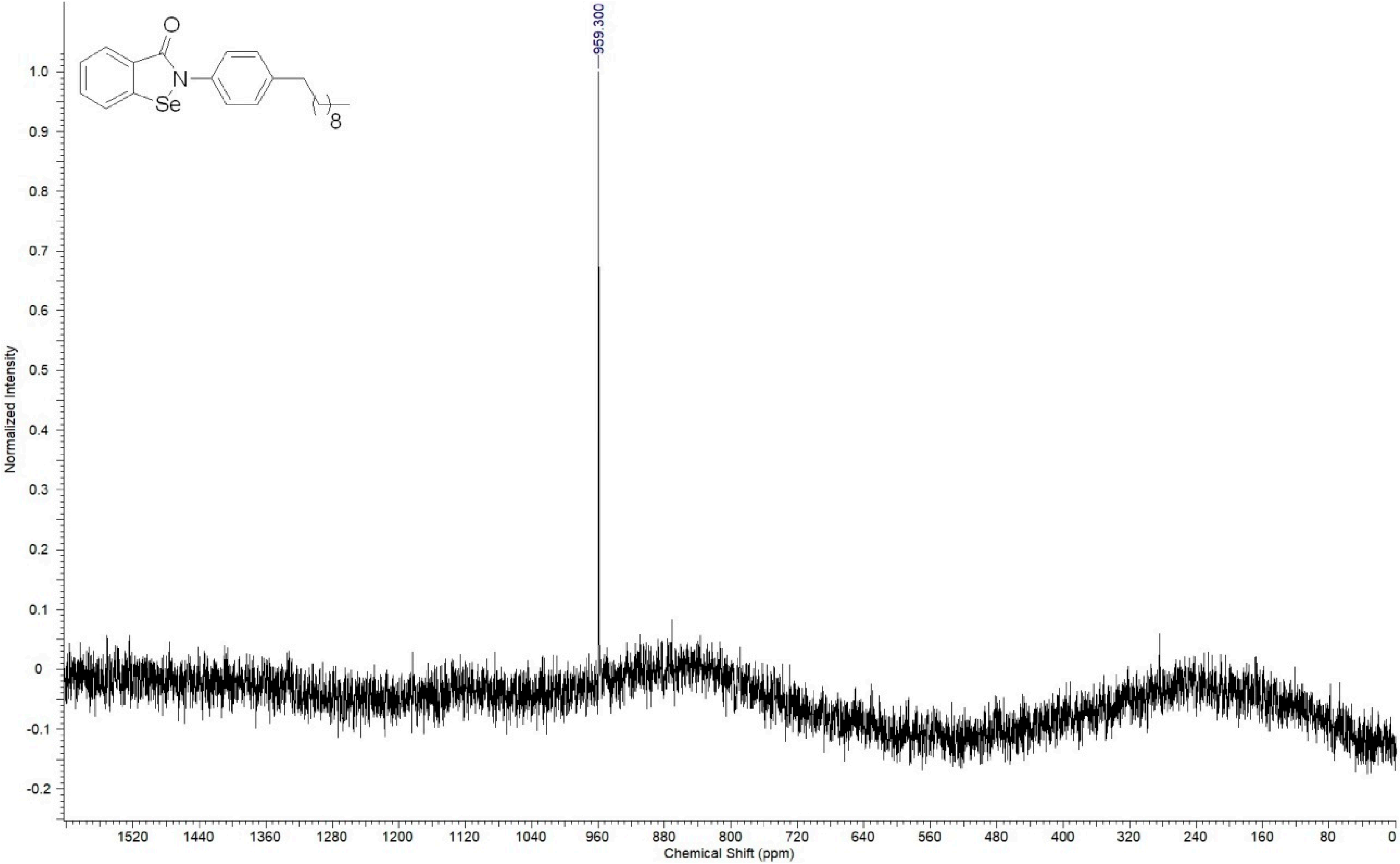
959.300
O
N
Se
8
Normalized Intensity
Chemical Shift (ppm)

N-4-(Dodecyl)phenyl-1,2-benzisoselenazol-3(2H)-one 21a

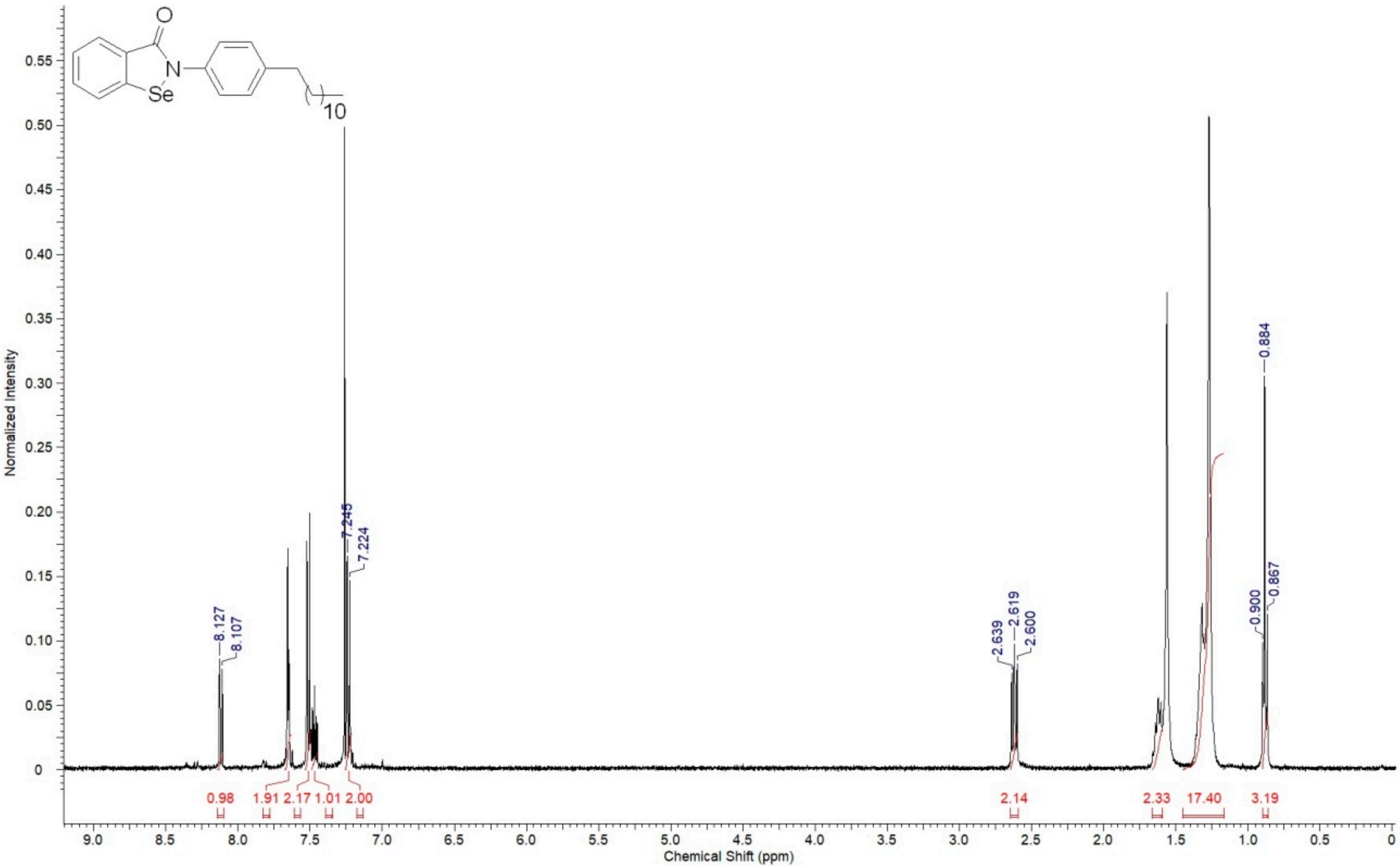

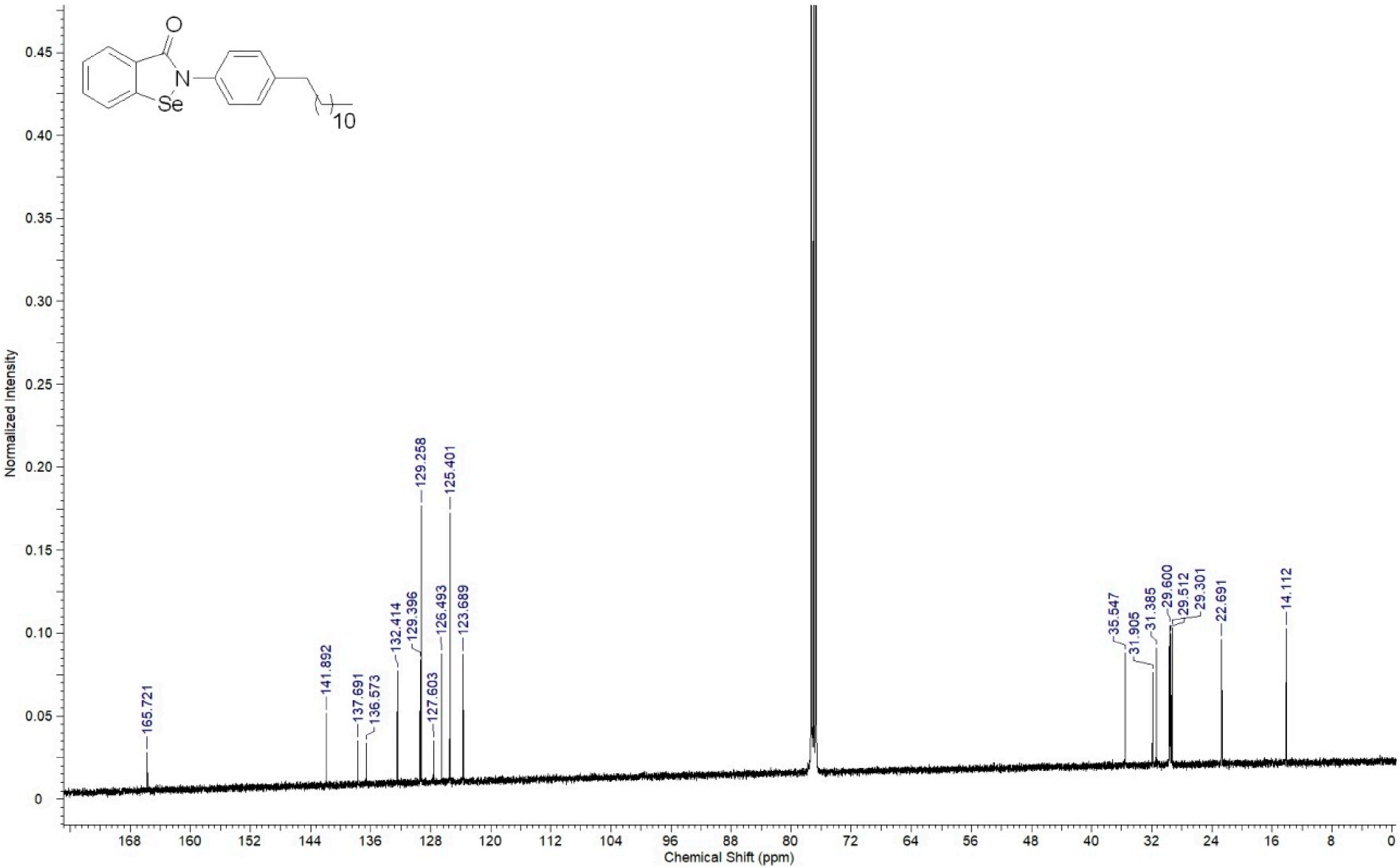
O
N
Se
10
Normalized Intensity
0.45
0.40
0.35
0.30
0.25
0.20
0.15
0.10
0.05
0
165.721
141.892
137.691
136.573
132.414
129.396
129.258
127.603
126.493
125.401
123.689
35.547
31.905
31.385
29.600
29.512
29.301
22.691
14.112
168
160
152
144
136
128
120
112
104
96
88
80
72
64
56
48
40
32
24
16
8
0
Chemical Shift (ppm)

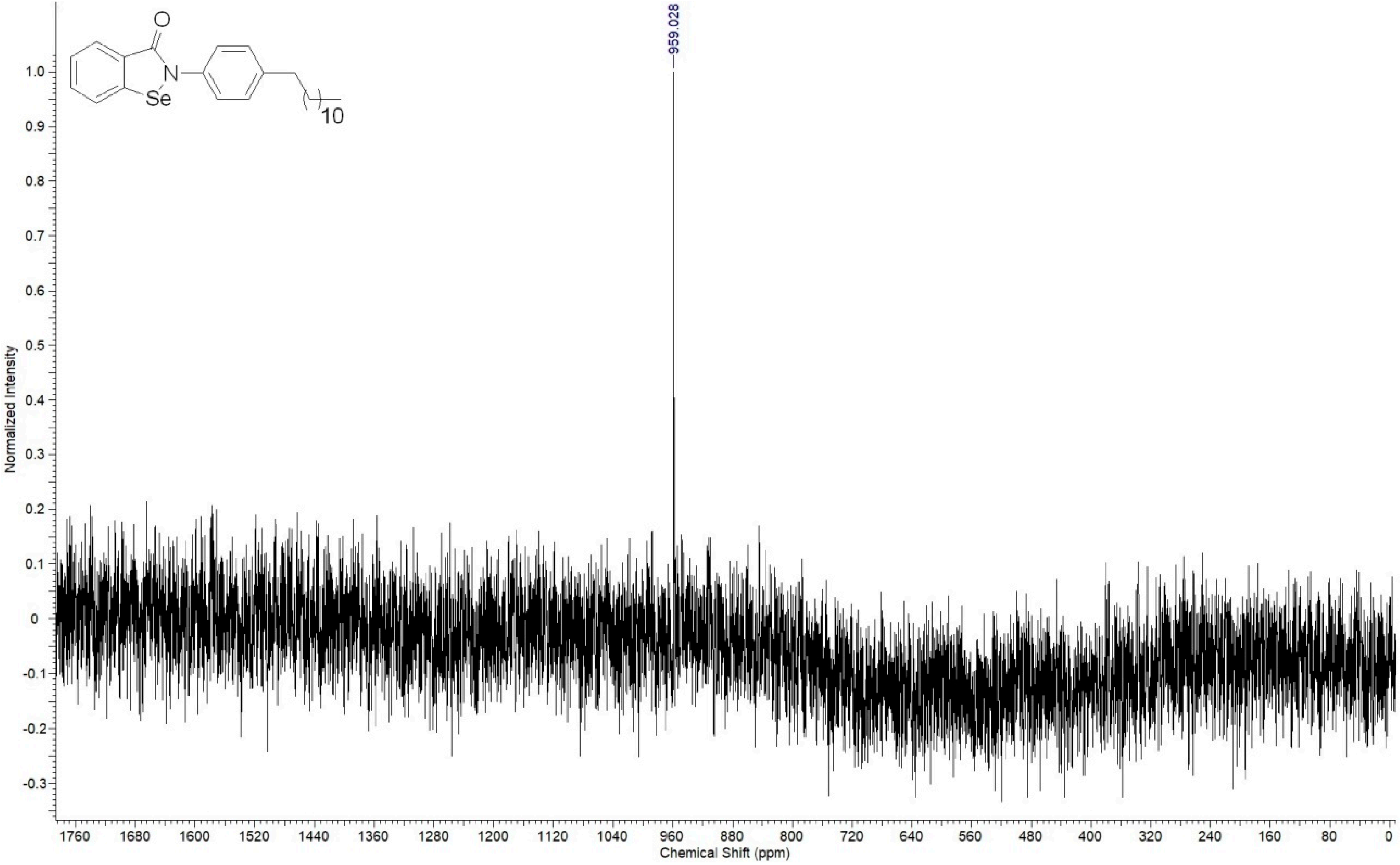
959.028
O
N
Se
10
Normalized Intensity
Chemical Shift (ppm)

Ethyl 6-(3-oxobenzo[d][1,2]selenazol-2(3H)-yl)hexanoate 22a

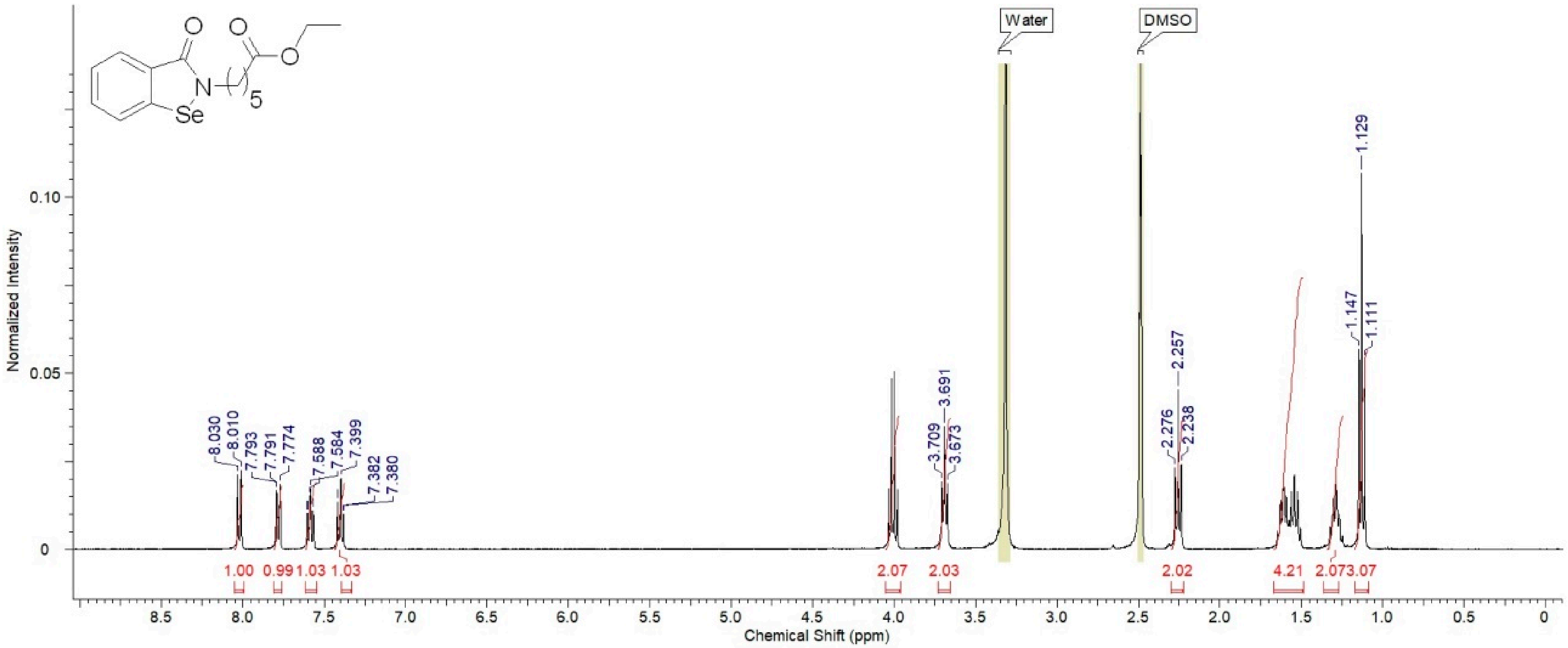

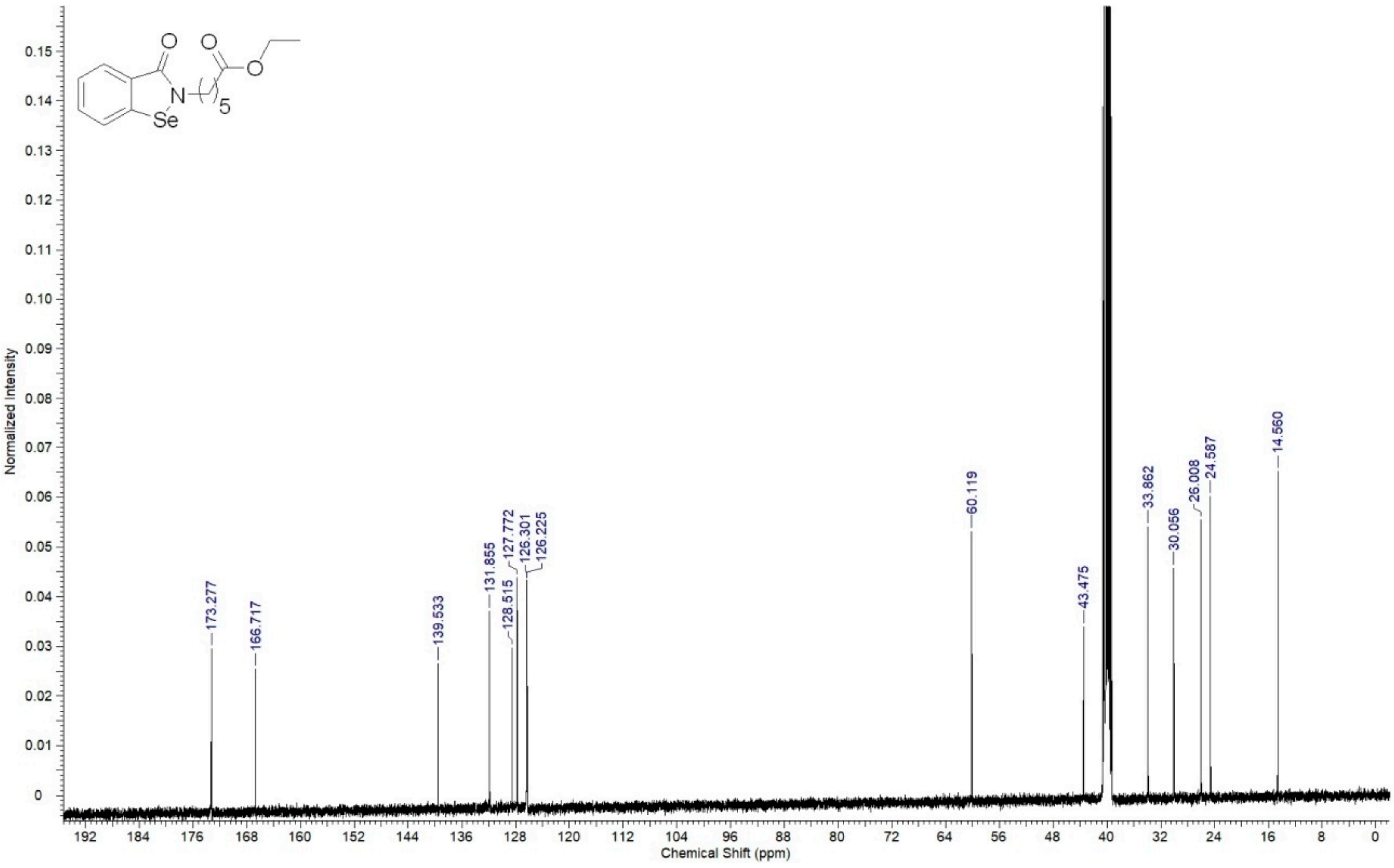

Se
N
5
O
O
O
0.15
0.14
0.13
0.12
0.11
0.10
0.09
0.08
0.07
0.06
0.05
0.04
0.03
0.02
0.01
0
Normalized Intensity
173.277
166.717
139.533
131.855
128.515
127.772
126.301
126.225
60.119
43.475
33.862
30.056
26.008
24.587
14.560
192
184
176
168
160
152
144
136
128
120
112
104
96
88
80
72
64
56
48
40
32
24
16
8
0
Chemical Shift (ppm)

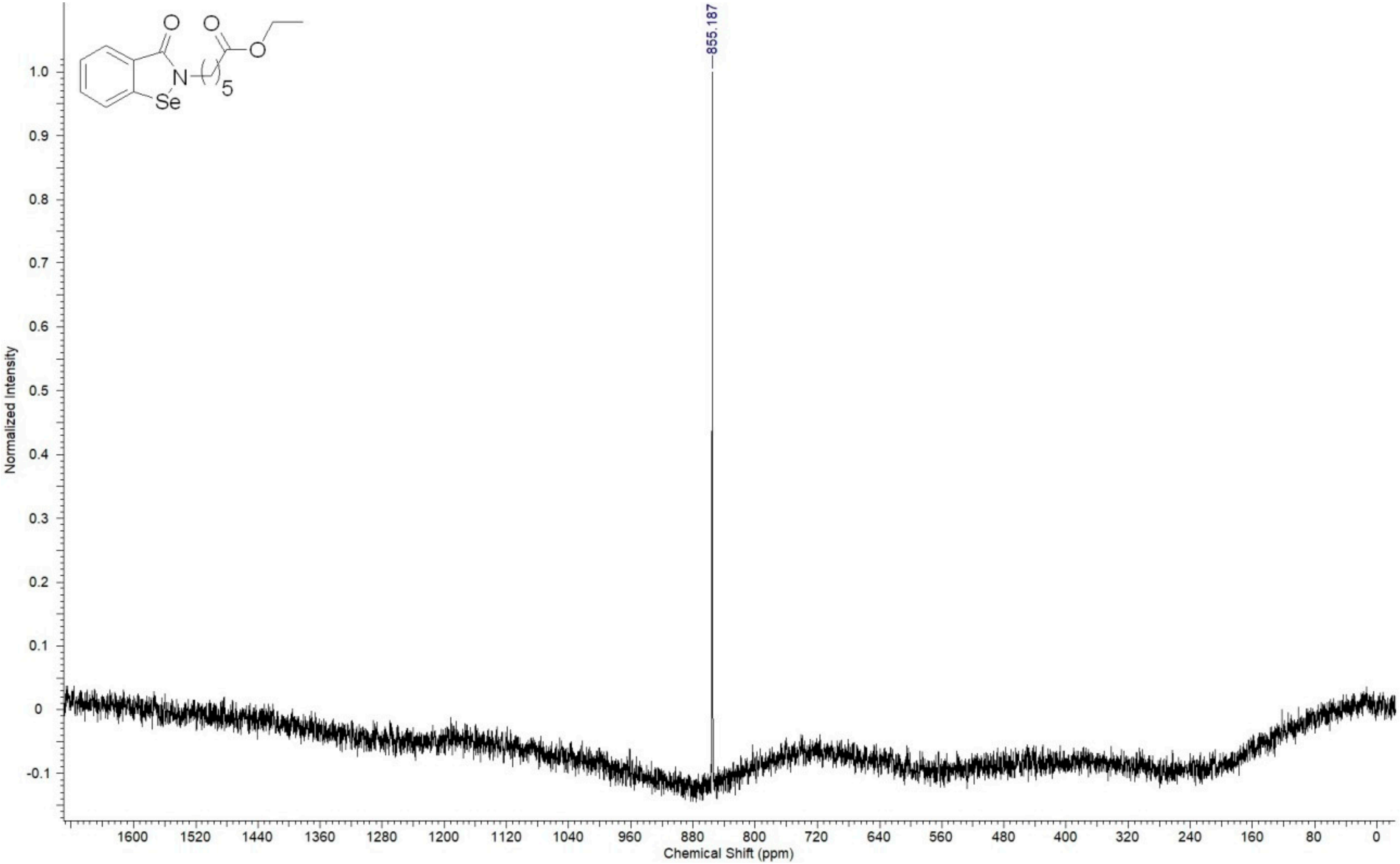

O
O
N
Se
5
O
855.187
Normalized Intensity
1.0
0.9
0.8
0.7
0.6
0.5
0.4
0.3
0.2
0.1
0
-0.1
1600
1520
1440
1360
1280
1200
1120
1040
960
880
800
720
640
560
480
400
320
240
160
80
0
Chemical Shift (ppm)

Ethyl 8-(3-oxobenzo[d][1,2]selenazol-2(3H)-yl)octanoate 23a

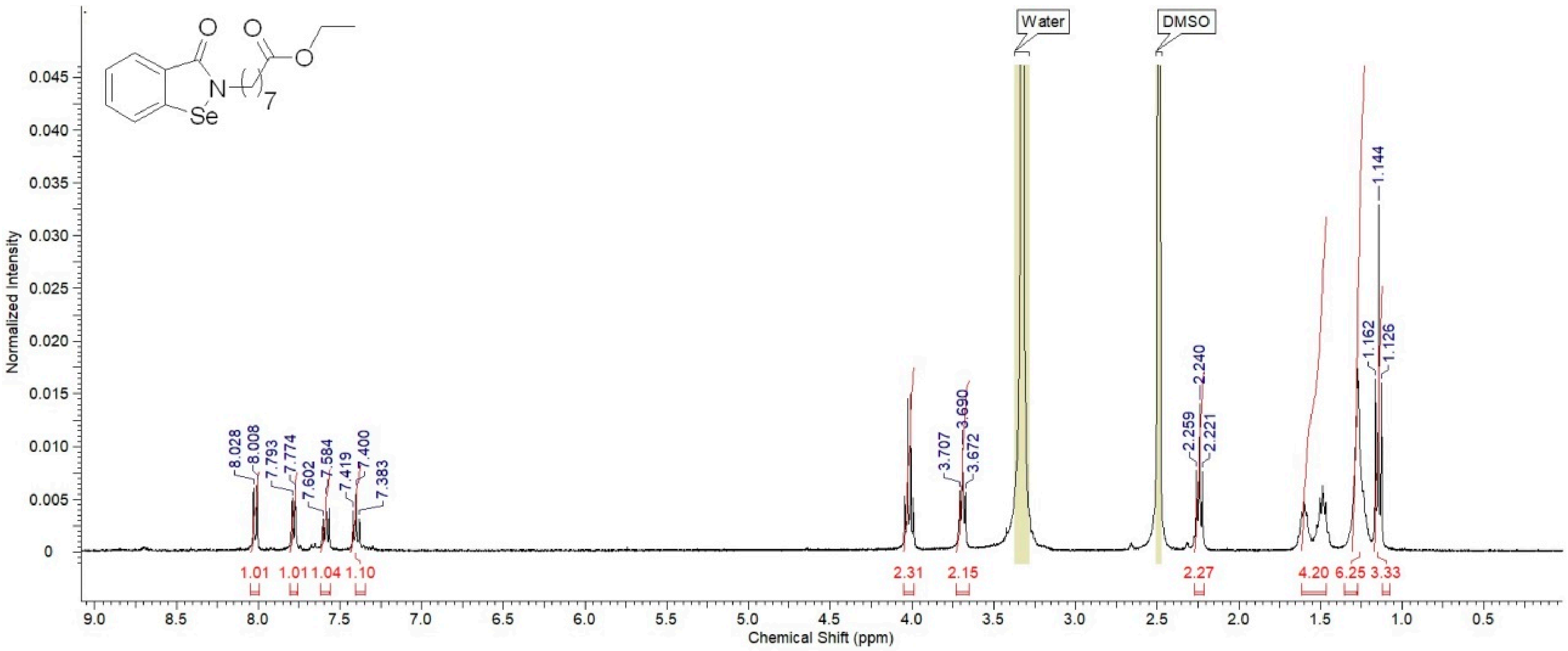

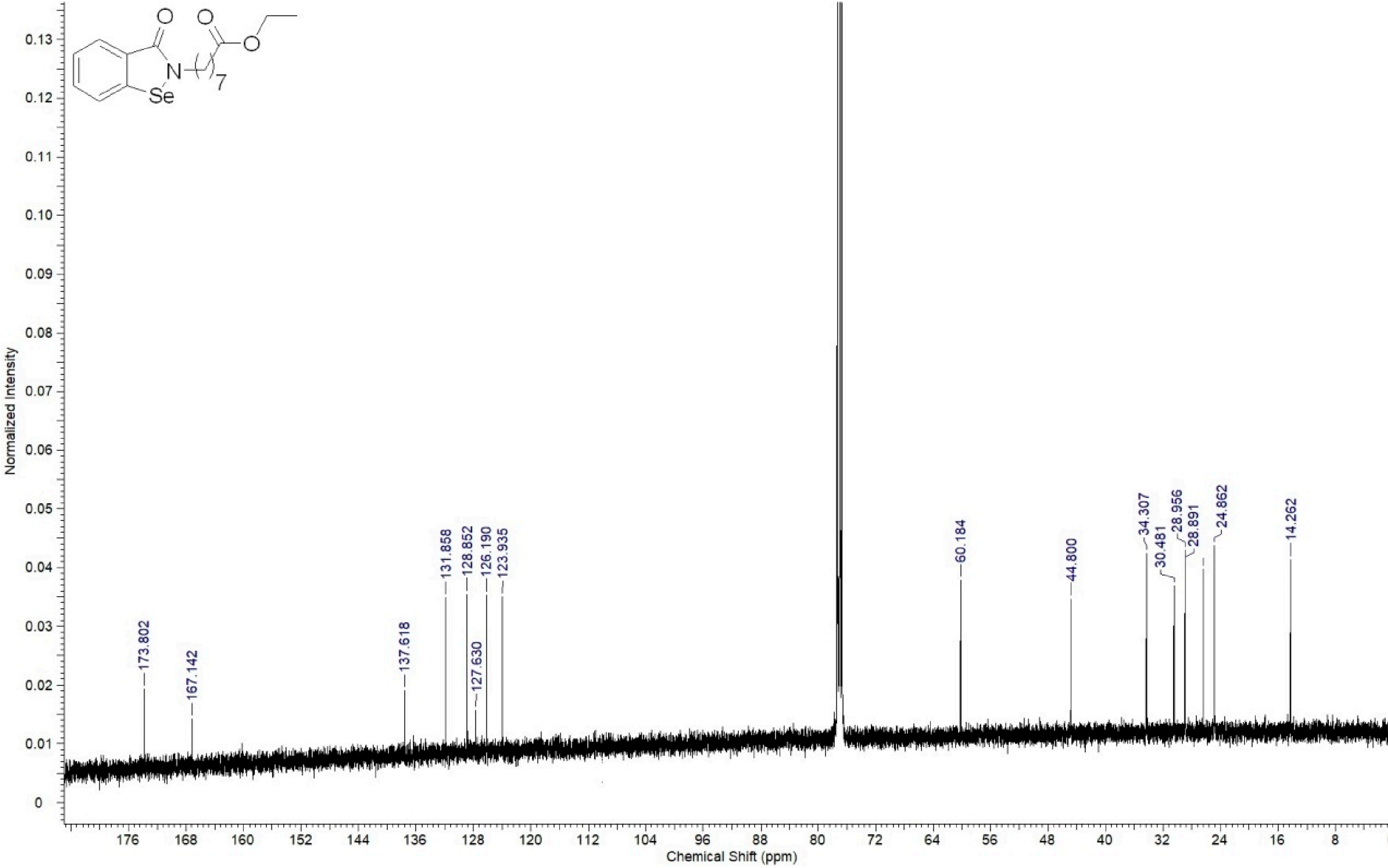

Normalized Intensity
Chemical Shift (ppm)
173.802
167.142
137.618
131.858
128.852
127.630
126.190
123.935
60.184
44.800
34.307
30.481
28.956
28.891
24.862
14.262

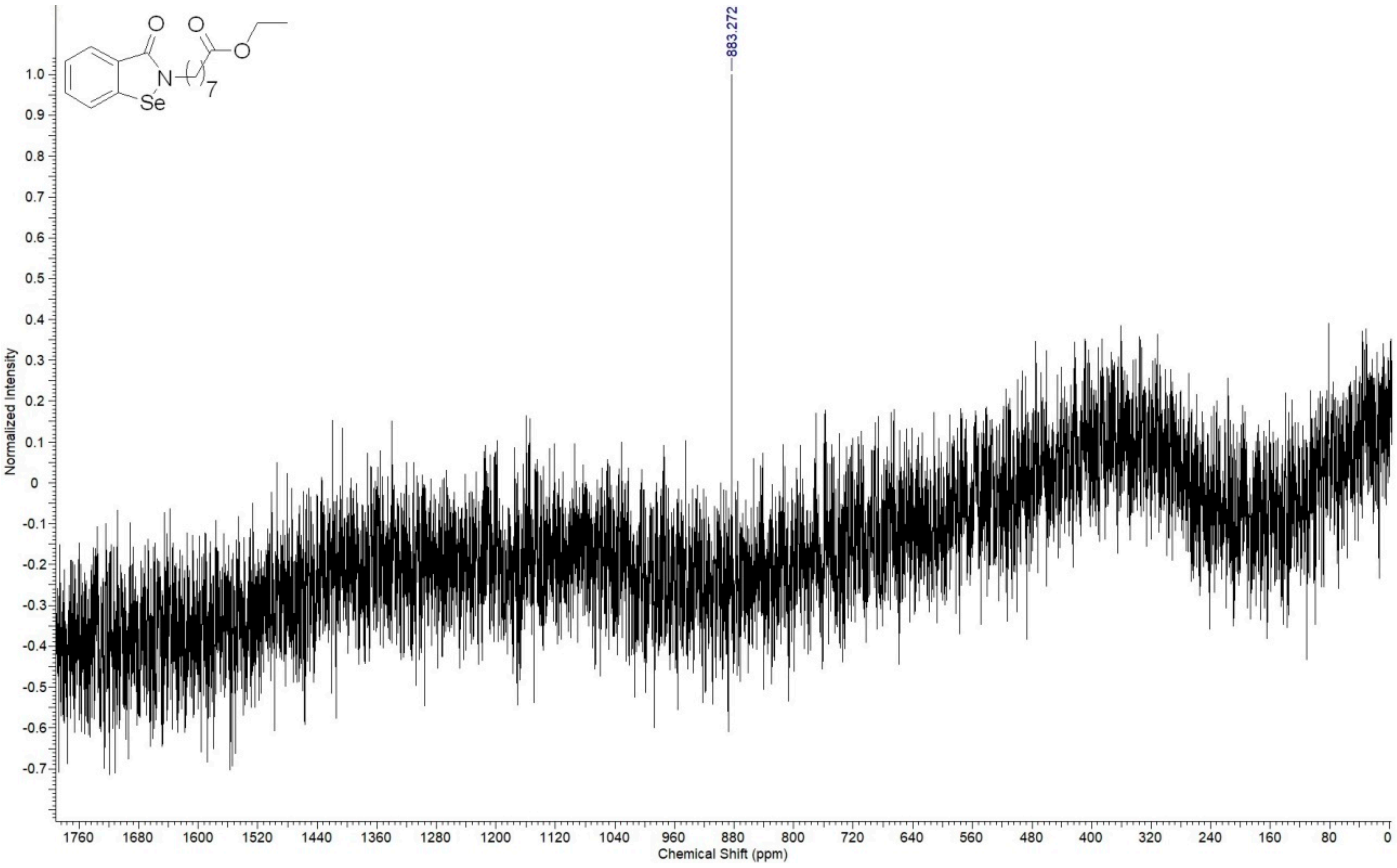

O
O
N
Se
O
7
883.272
Normalized Intensity
1.0
0.9
0.8
0.7
0.6
0.5
0.4
0.3
0.2
0.1
0
-0.1
-0.2
-0.3
-0.4
-0.5
-0.6
-0.7
1760
1680
1600
1520
1440
1360
1280
1200
1120
1040
960
880
800
720
640
560
480
400
320
240
160
80
0
Chemical Shift (ppm)

Ethyl 12-(3-oxobenzo[d][1,2]selenazol-2(3H)-yl)dodecanoate 24a

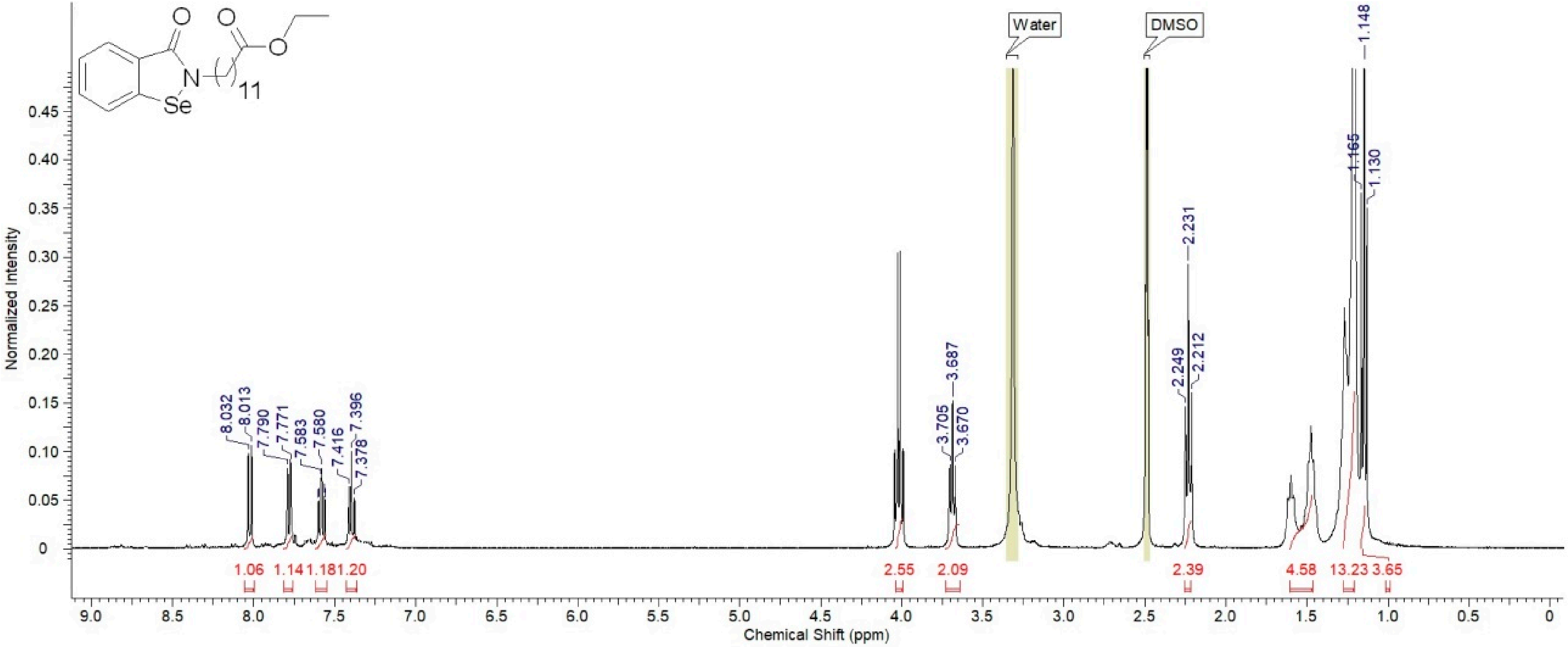

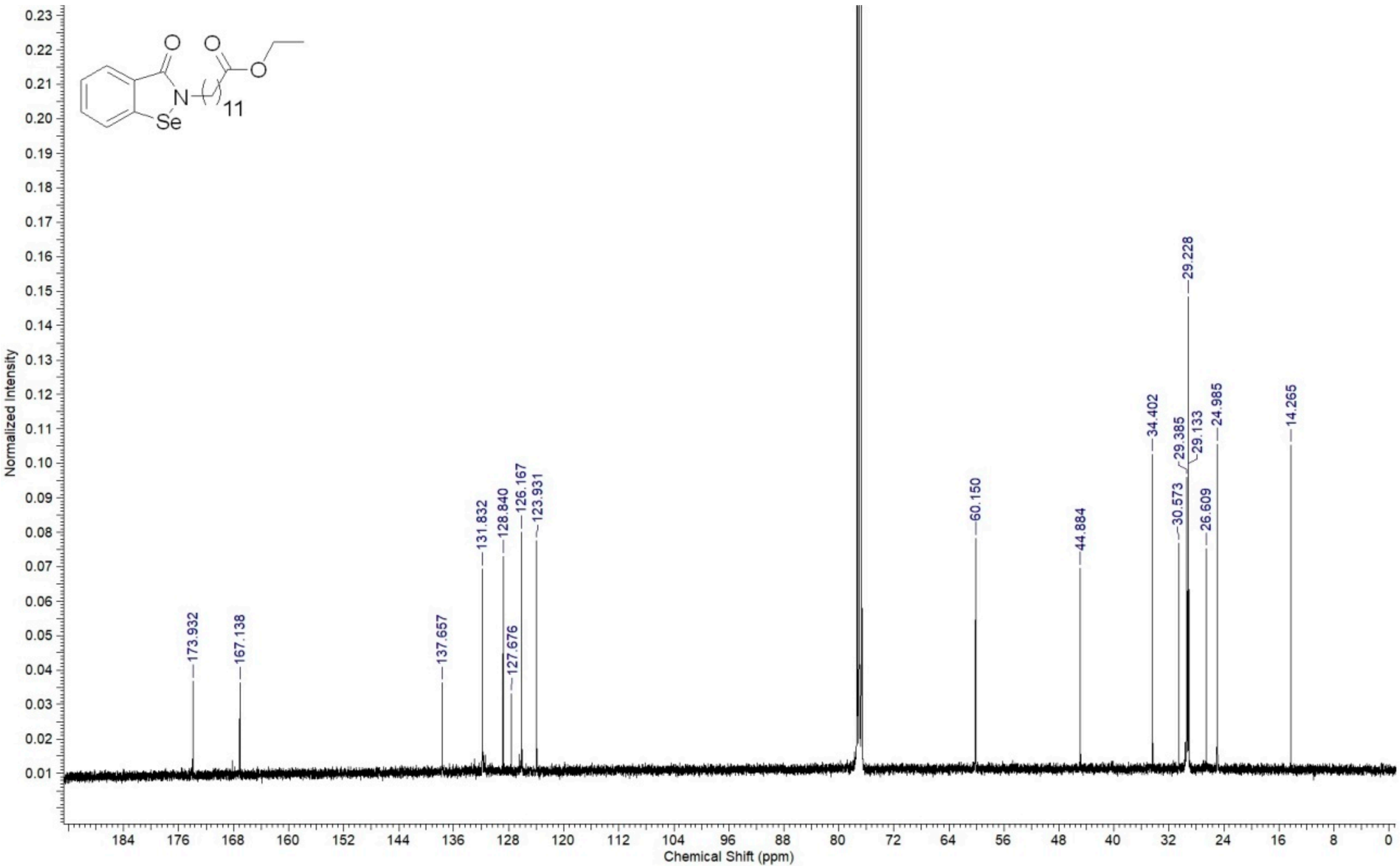

O
O
N
Se
11
O
173.932
167.138
137.657
131.832
128.840
127.676
126.167
123.931
60.150
44.884
34.402
30.573
29.385
29.228
29.133
26.609
24.985
14.265
Normalized Intensity
Chemical Shift (ppm)

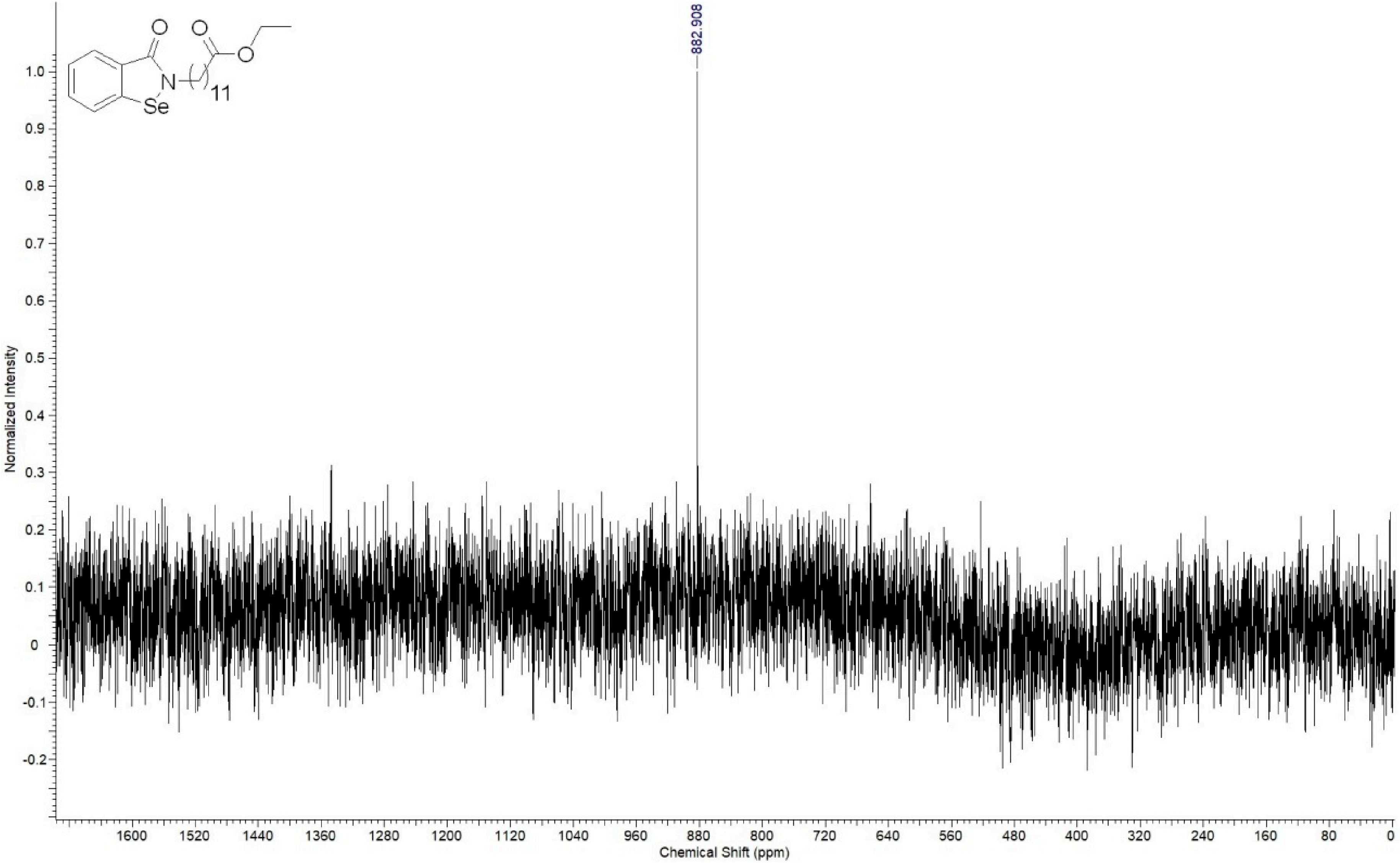
882.908
Normalized Intensity
Chemical Shift (ppm)
O
O
O
N
Se
11

2,2'-Diselenobis((*N*-octyl)benzamide) 17b

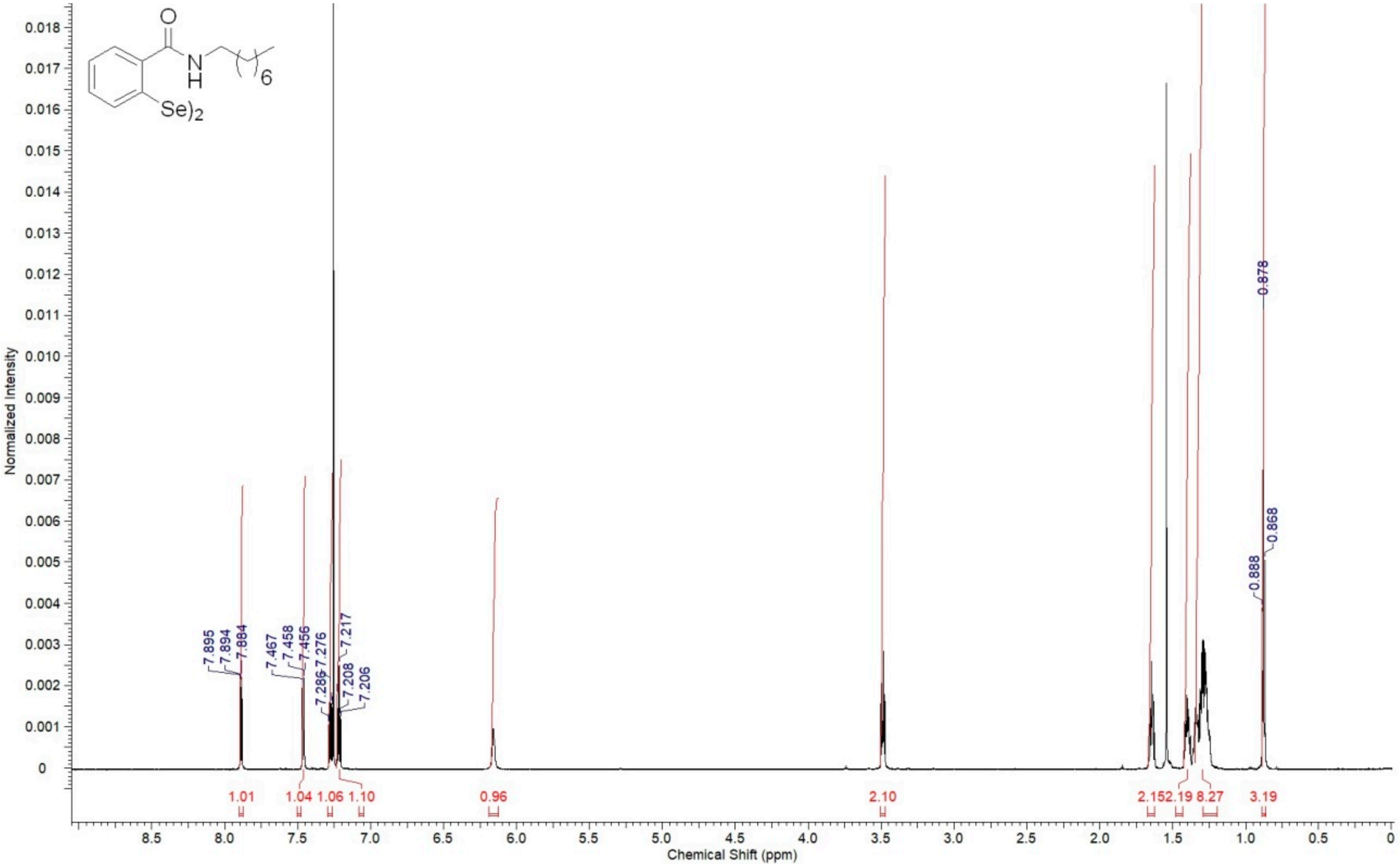

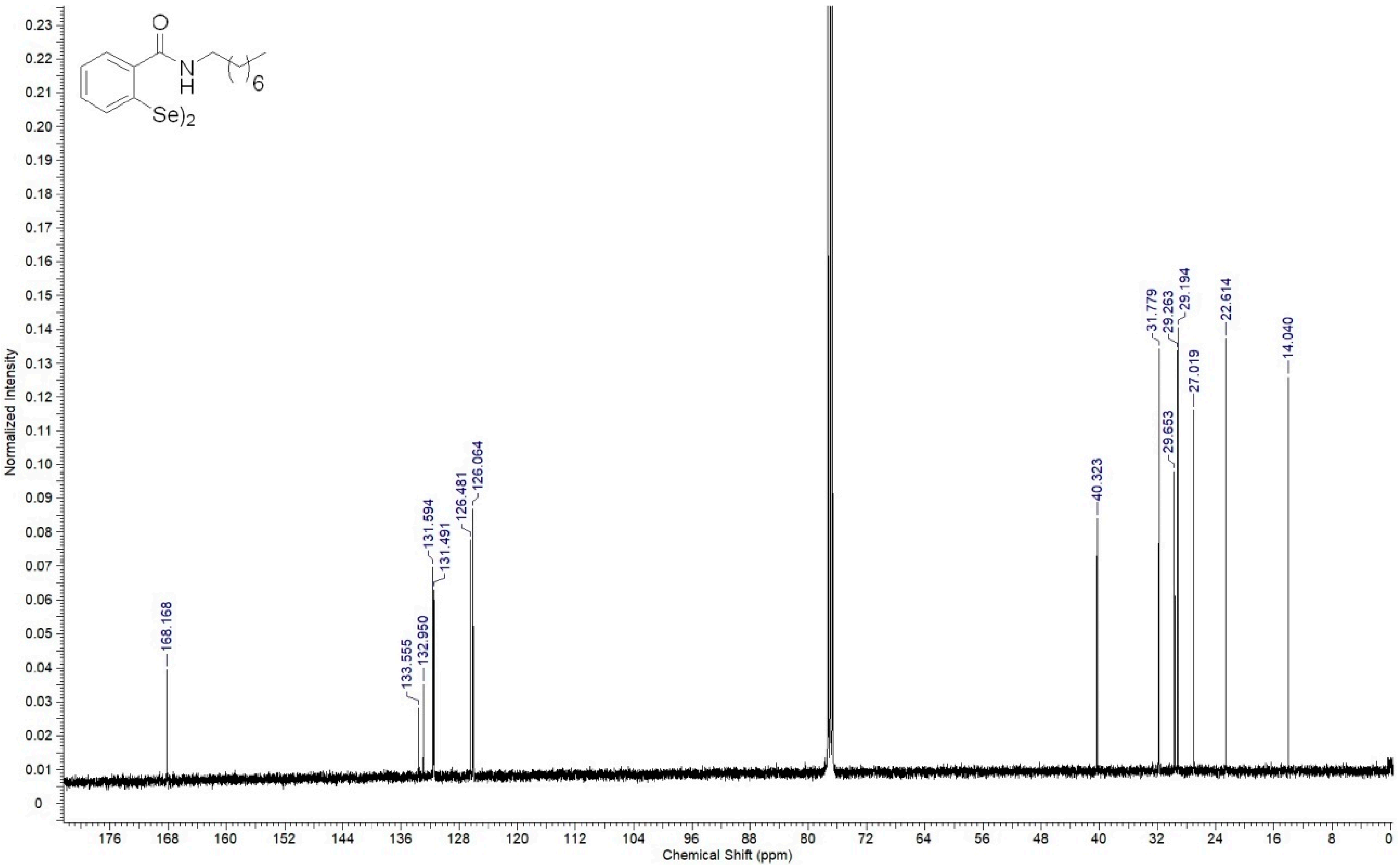

O
N
H
6
Se)2
Normalized Intensity
0.23
0.22
0.21
0.20
0.19
0.18
0.17
0.16
0.15
0.14
0.13
0.12
0.11
0.10
0.09
0.08
0.07
0.06
0.05
0.04
0.03
0.02
0.01
0
168.168
133.555
132.950
131.594
131.491
126.481
126.064
40.323
31.779
29.653
29.263
29.194
27.019
22.614
14.040
176
168
160
152
144
136
128
120
112
104
96
88
80
72
64
56
48
40
32
24
16
8
0
Chemical Shift (ppm)

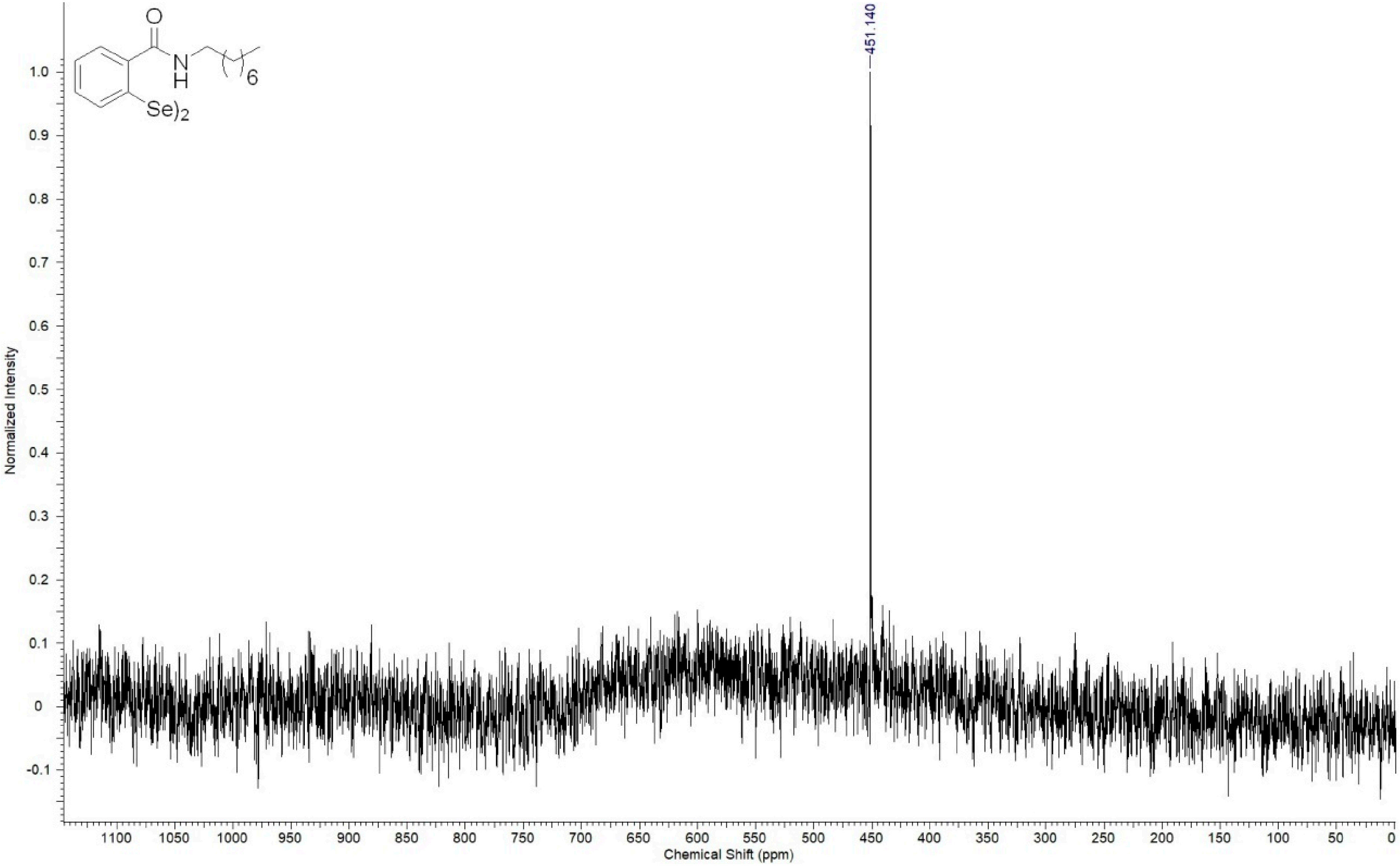

O
N
H
6
Se)2
451.140
Normalized Intensity
Chemical Shift (ppm)

2,2'-Diselenobis((*N*-decyl)benzamide) 18b

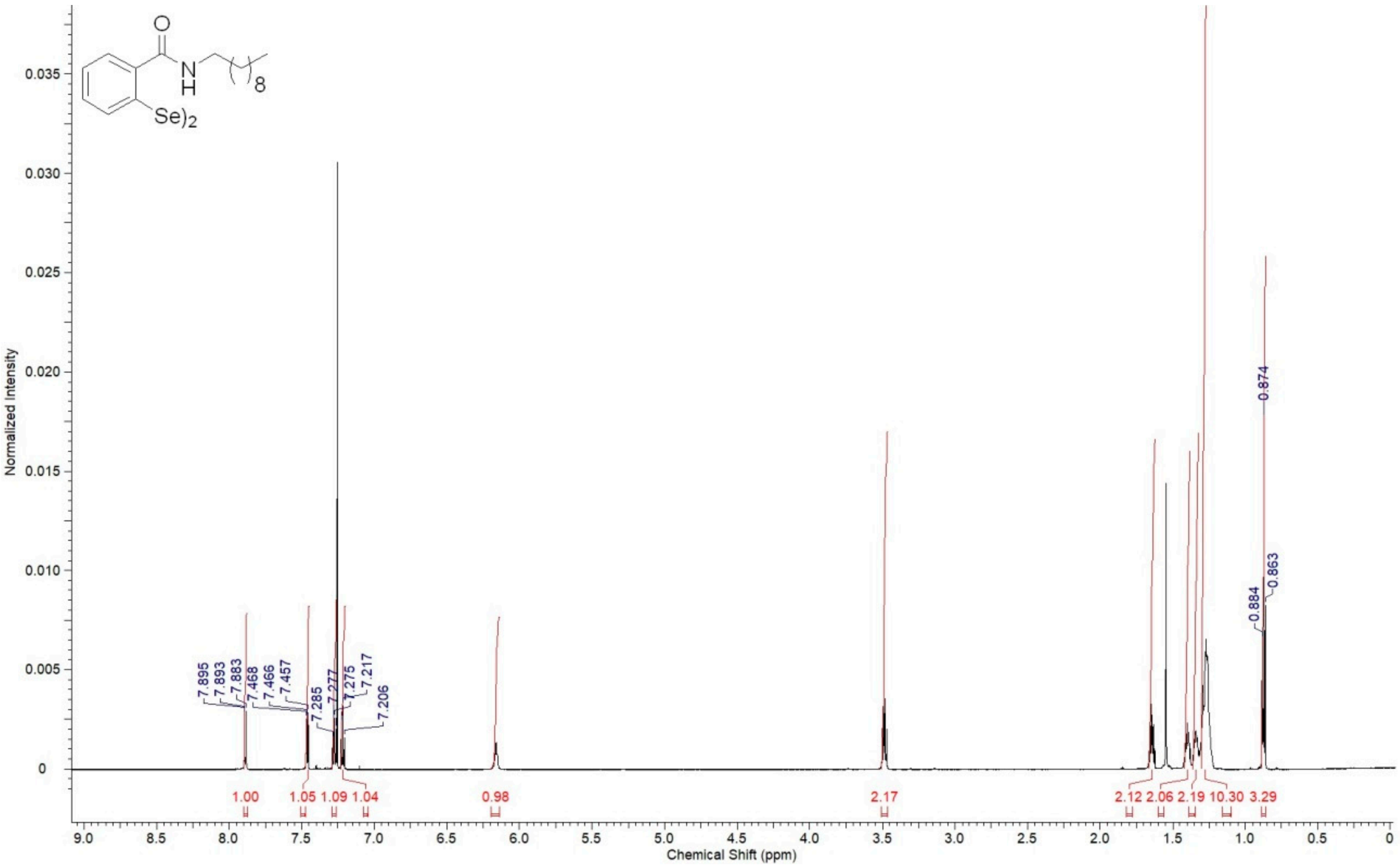

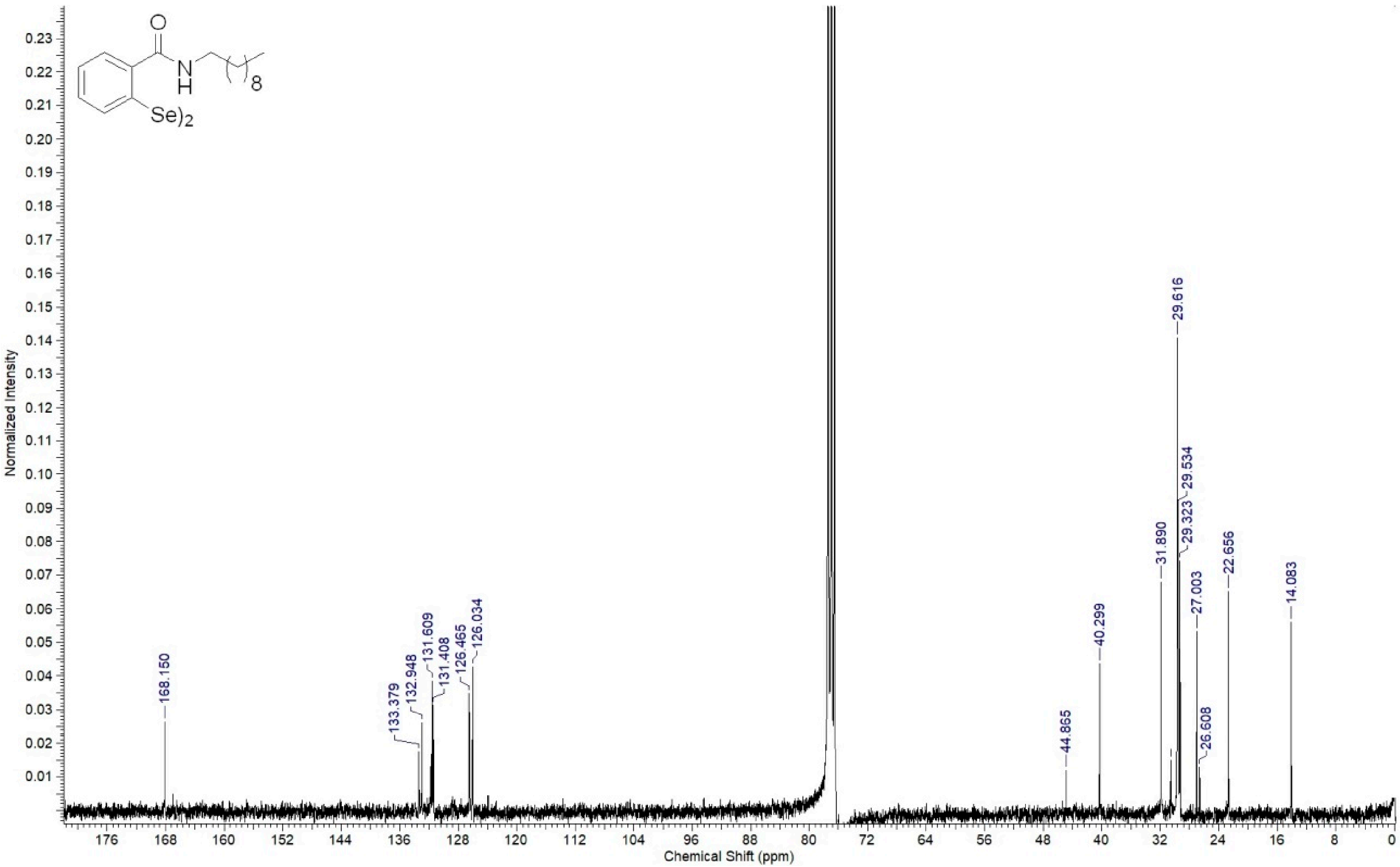
O
N
H
8
Se)2
Normalized Intensity
Chemical Shift (ppm)
168.150
133.379
132.948
131.609
131.408
126.465
126.034
44.865
40.299
31.890
29.616
29.534
29.323
27.003
26.608
22.656
14.083

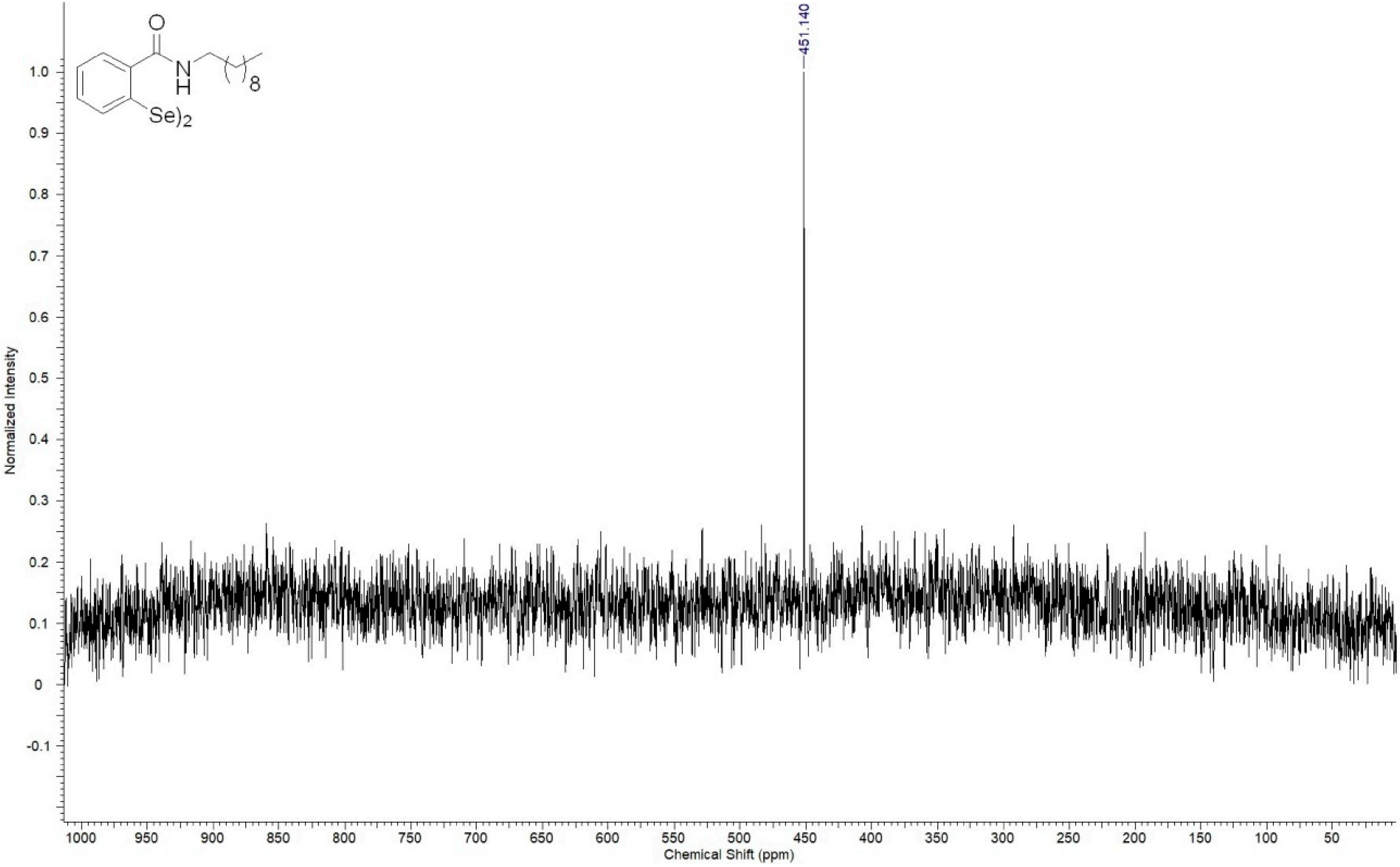
451.140
Normalized Intensity
Chemical Shift (ppm)

2,2'-Diselenobis((*N*-dodecyl)benzamide) 19b

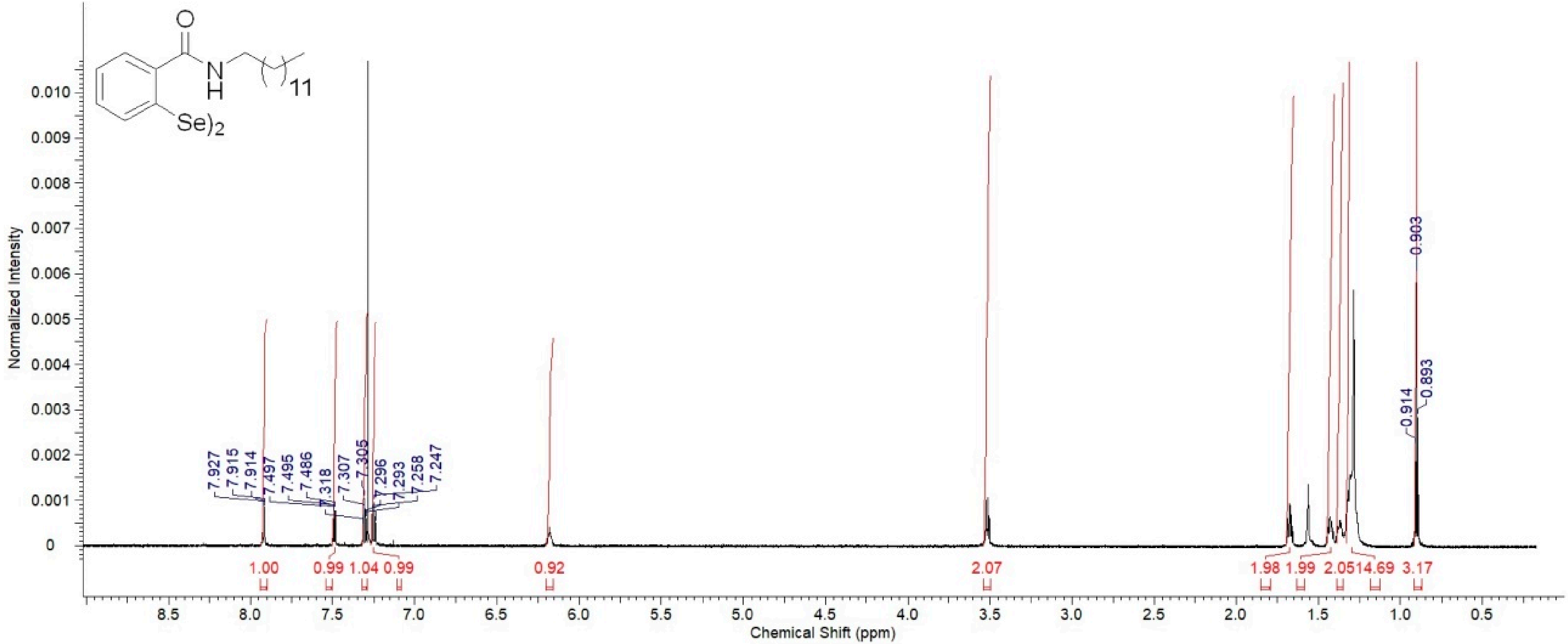

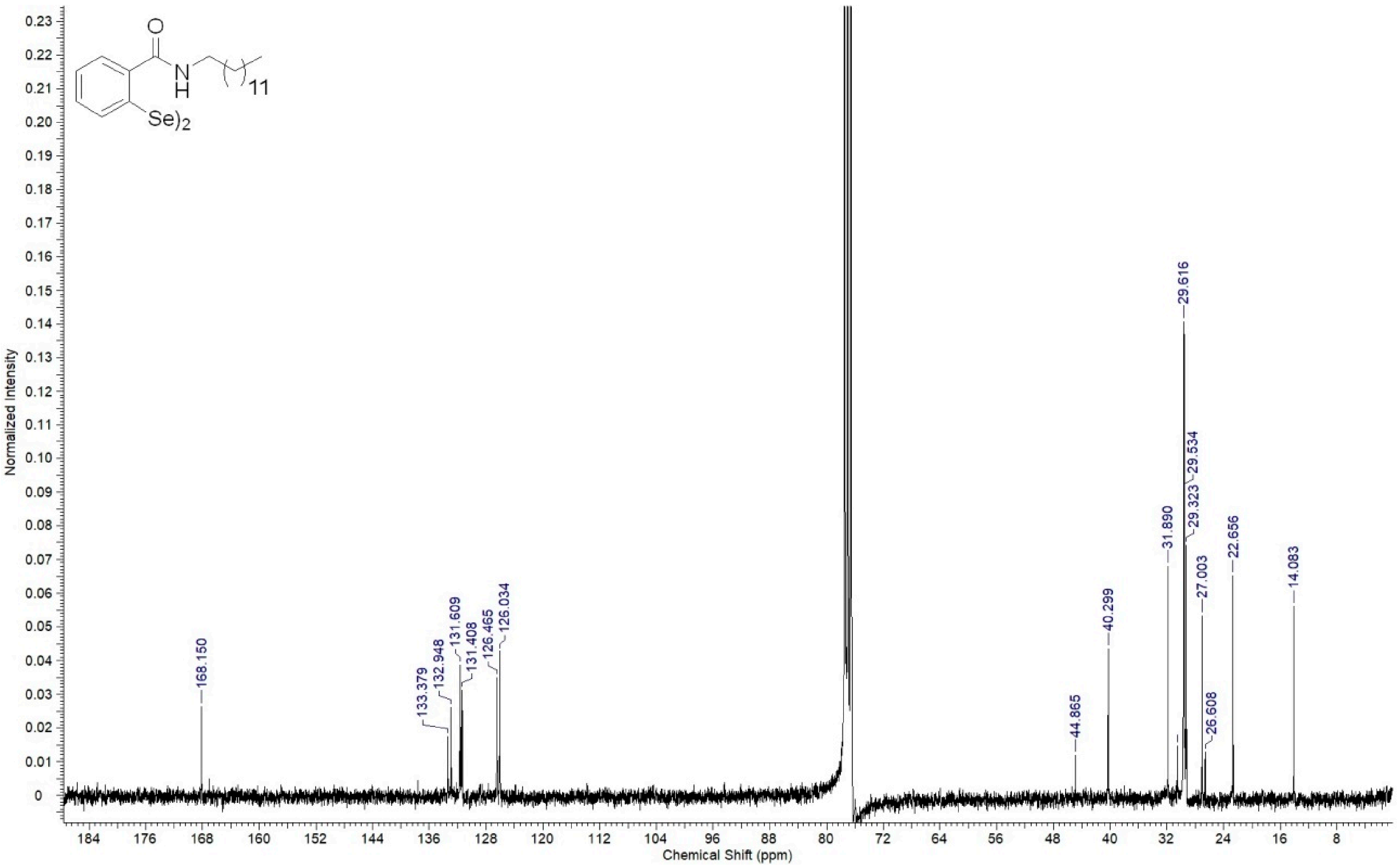

O
N
H
11
Se)2
168.150
133.379
132.948
131.609
131.408
126.465
126.034
44.865
40.299
31.890
29.616
29.534
29.323
27.003
26.608
22.656
14.083
Normalized Intensity
Chemical Shift (ppm)

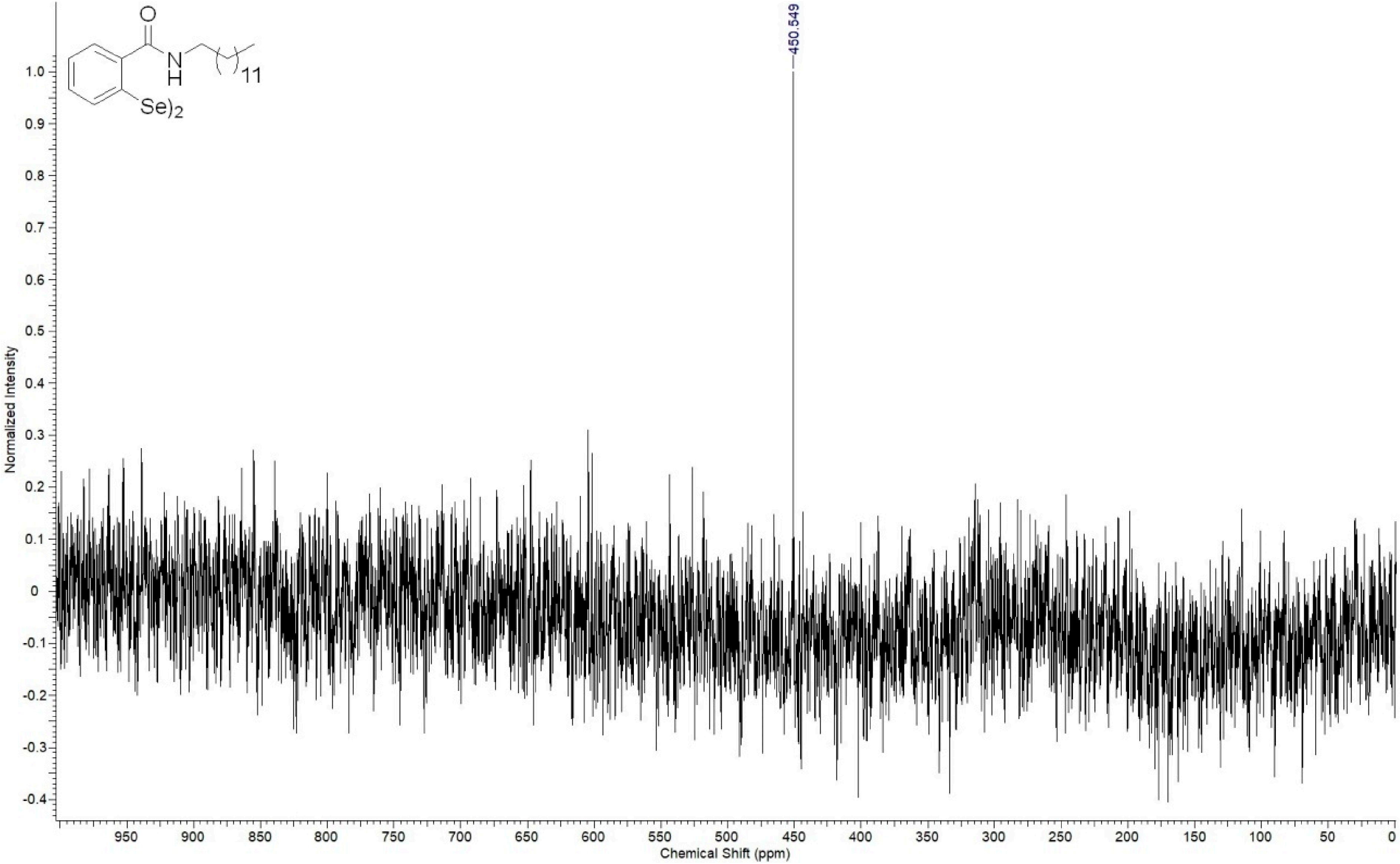

O
N
H
11
Se)2
450.549
Normalized Intensity
Chemical Shift (ppm)

2,2'-Diselenobis((4-(decyl)phenyl)benzamide) 20b

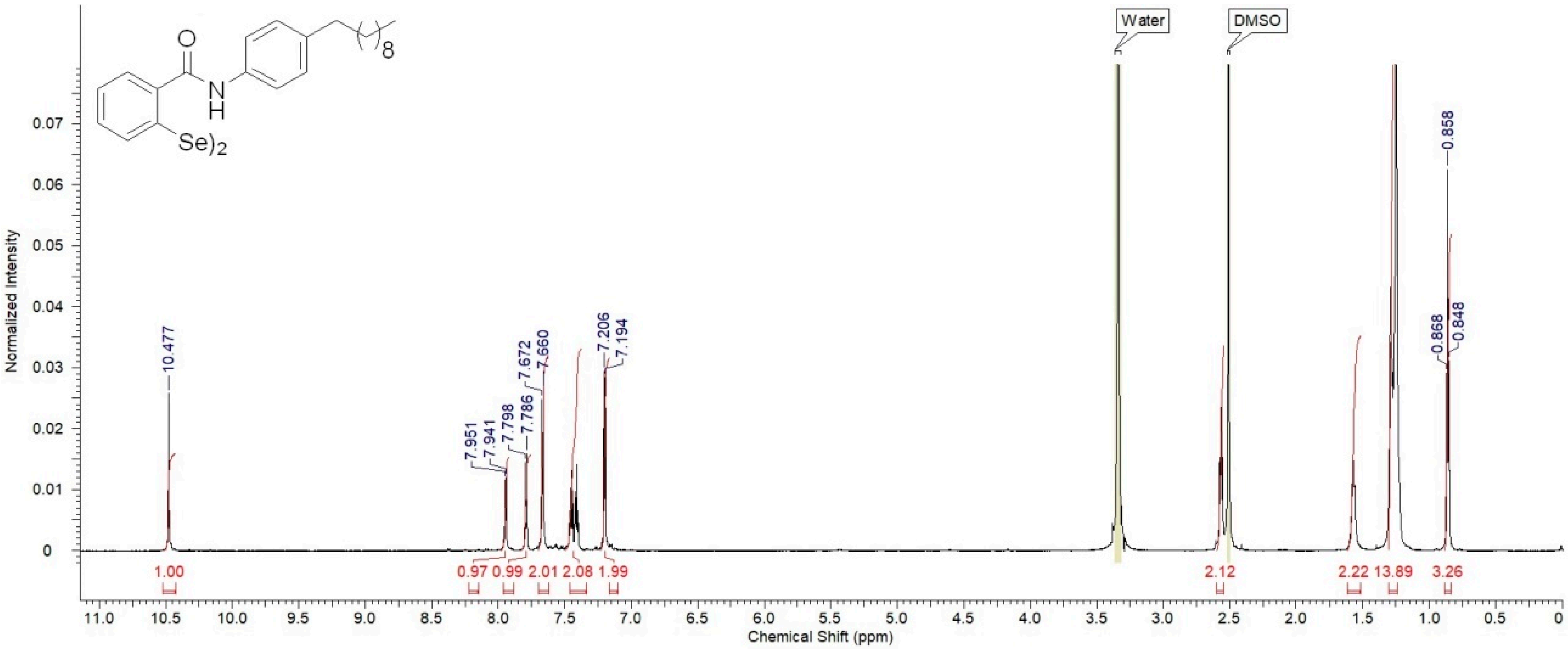

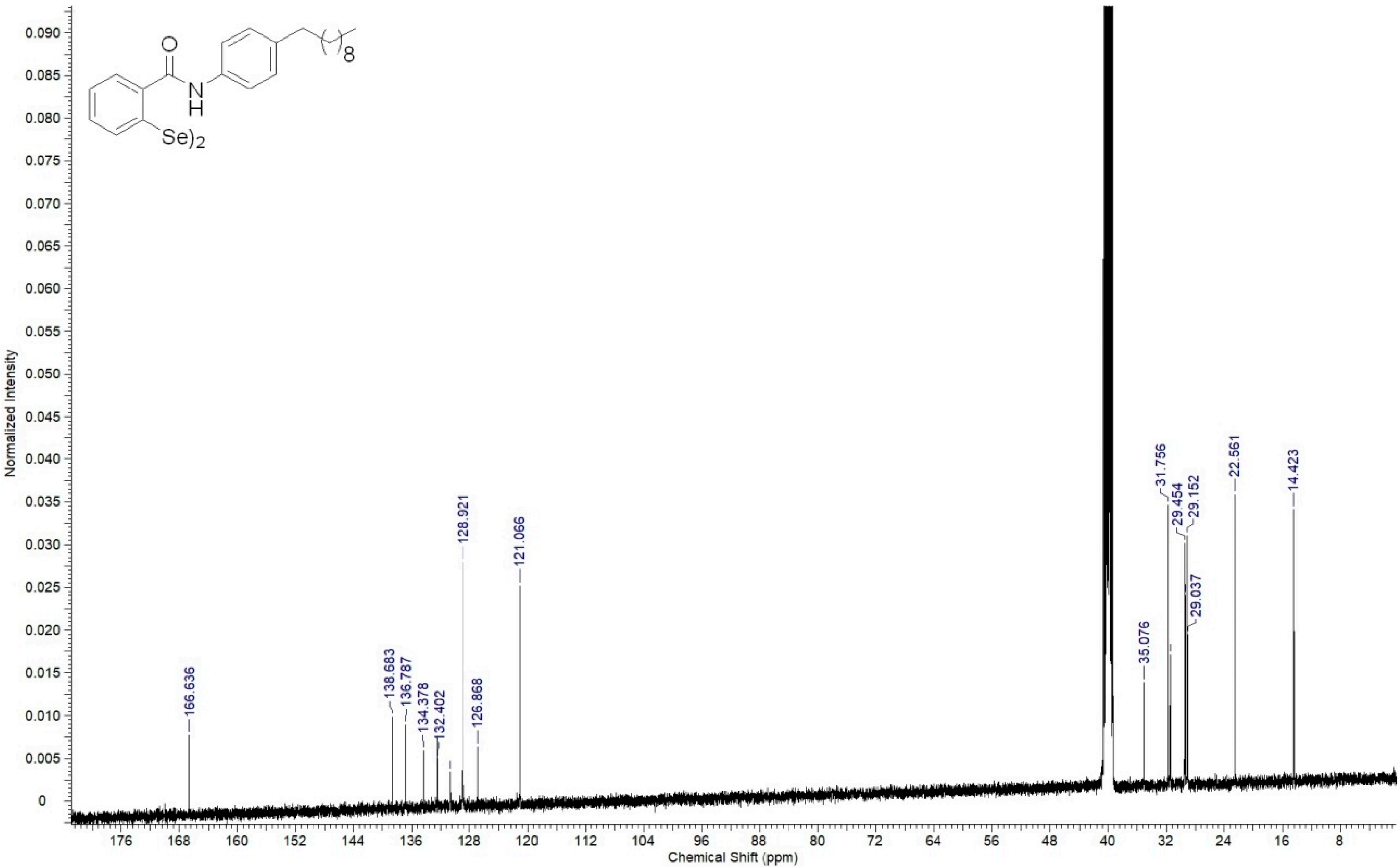
Normalized Intensity
Chemical Shift (ppm)
Se)2
166.636
138.683
136.787
134.378
132.402
128.921
126.868
121.066
35.076
31.756
29.454
29.152
29.037
22.561
14.423

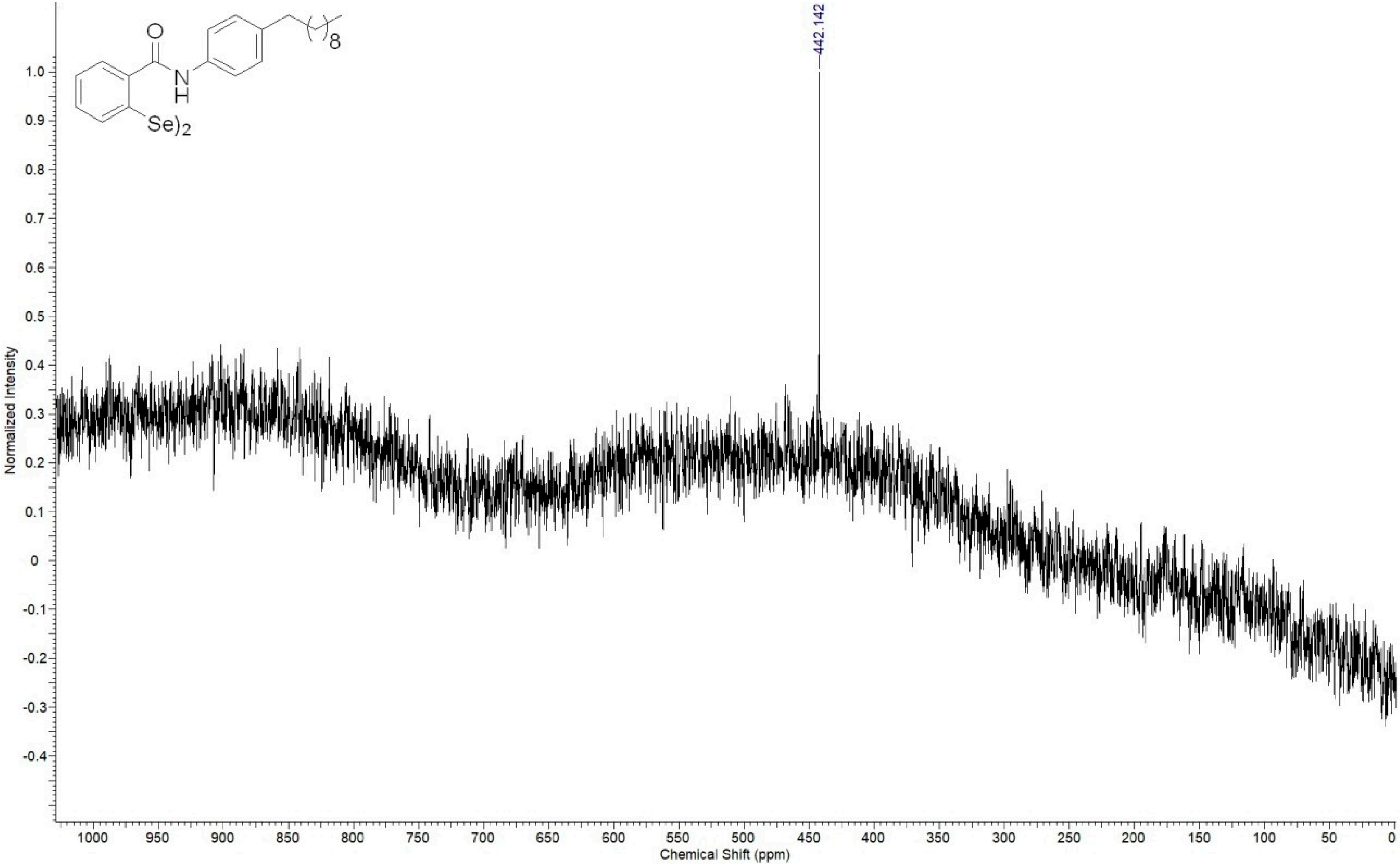
442.142
O
N
H
Se)2
8
Normalized Intensity
1.0
0.9
0.8
0.7
0.6
0.5
0.4
0.3
0.2
0.1
0
-0.1
-0.2
-0.3
-0.4
1000 950 900 850 800 750 700 650 600 550 500 450 400 350 300 250 200 150 100 50 0
Chemical Shift (ppm)

2,2'-Diselenobis((4-(dodecyl)phenyl)benzamide) 21b

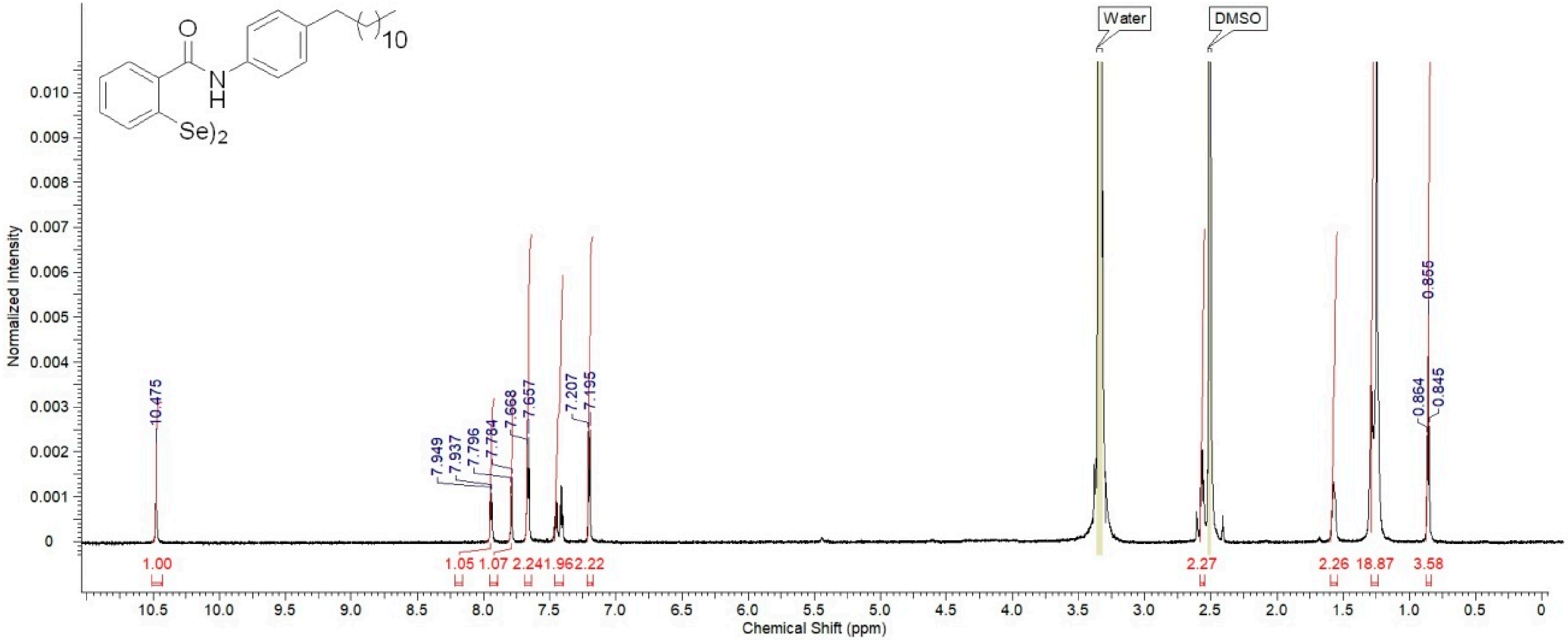

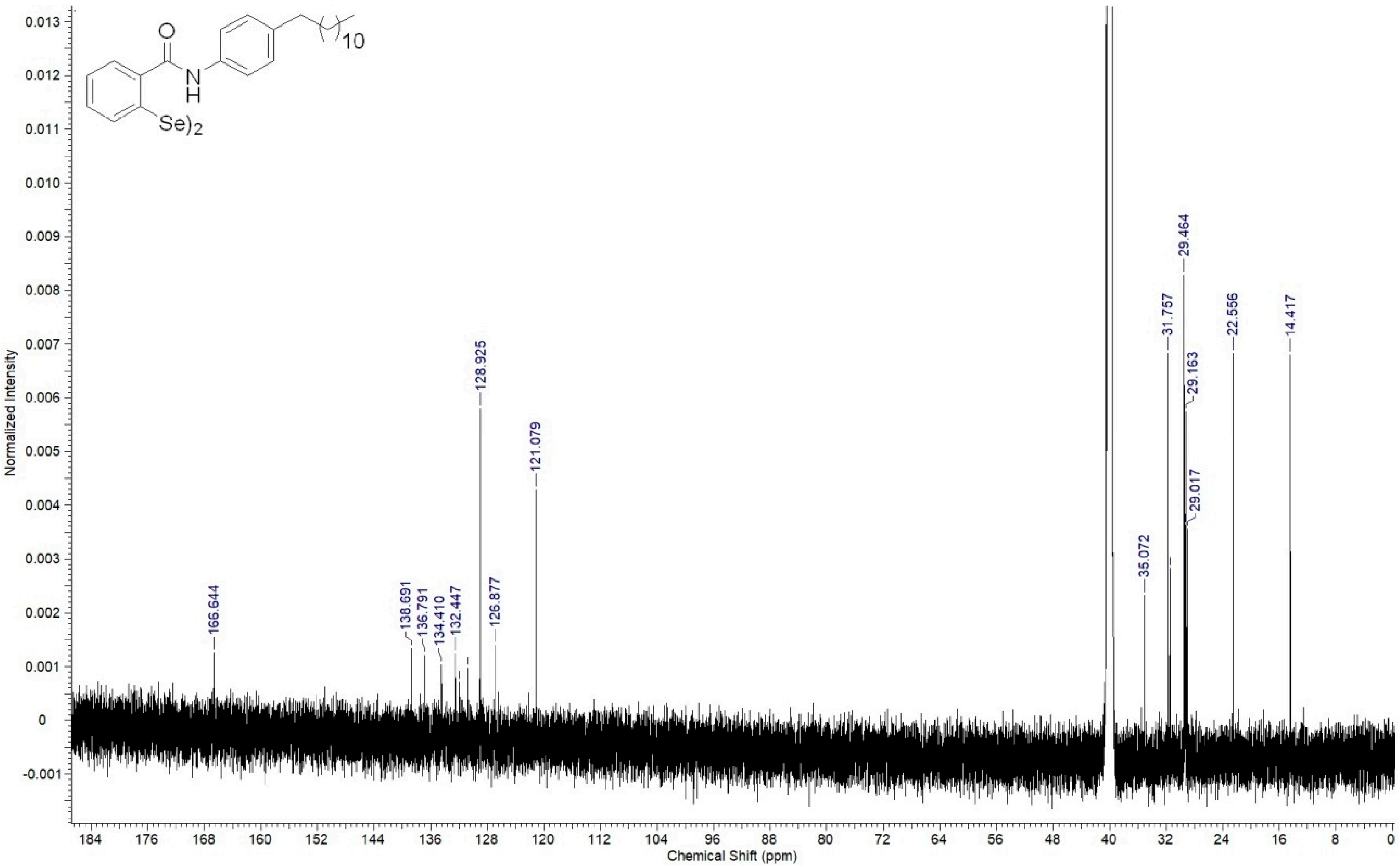

O
N
H
Se)2
10
0.013
0.012
0.011
0.010
0.009
0.008
0.007
0.006
0.005
0.004
0.003
0.002
0.001
0
-0.001
Normalized Intensity
166.644
138.691
136.791
134.410
132.447
128.925
126.877
121.079
35.072
31.757
29.464
29.163
29.017
22.556
14.417
184
176
168
160
152
144
136
128
120
112
104
96
88
80
72
64
56
48
40
32
24
16
8
0
Chemical Shift (ppm)

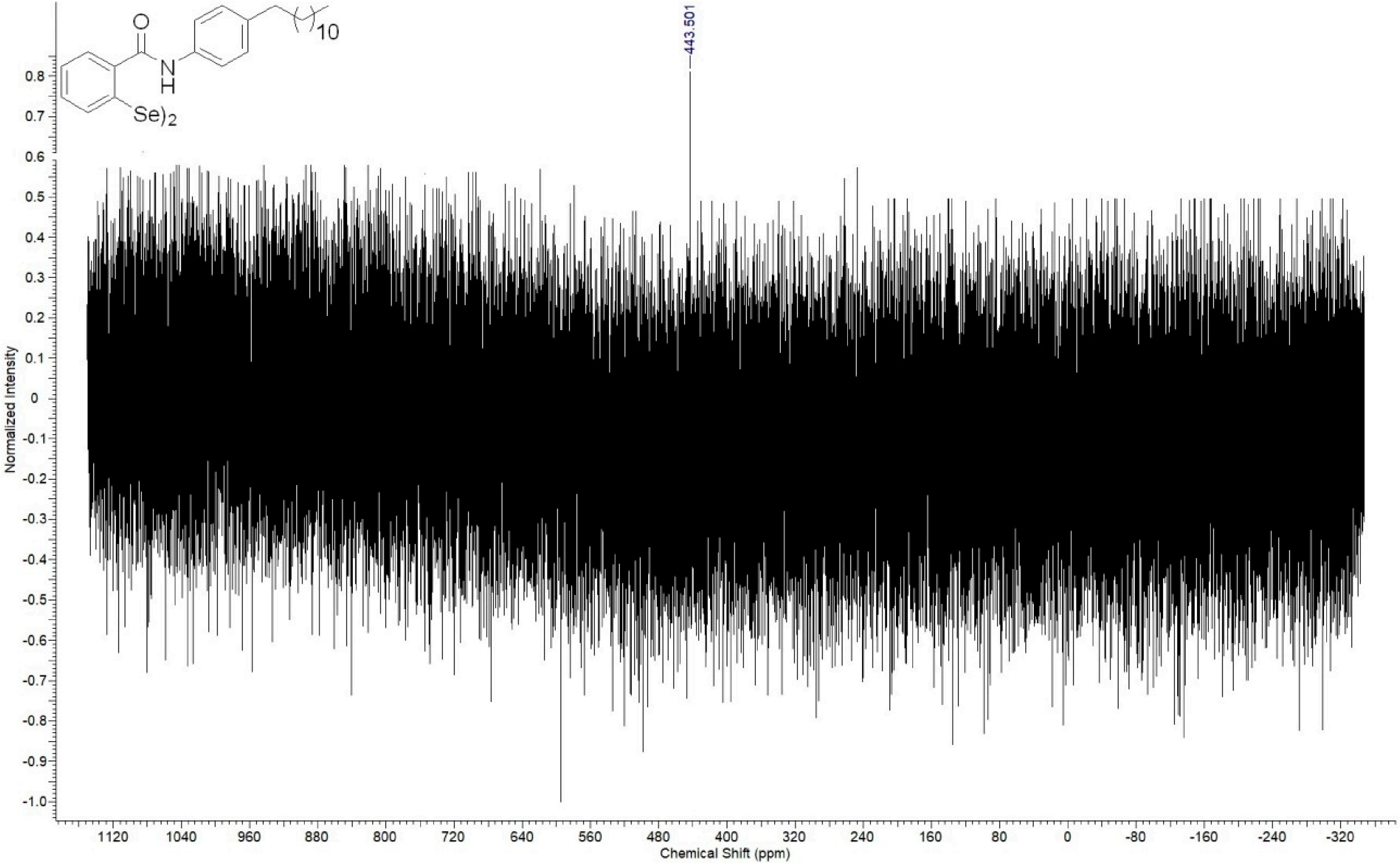

Se)2
10
443.501
Normalized Intensity
Chemical Shift (ppm)

Diethyl 6,6'-((2,2'-diselanediylbis(benzoyl))bis(azanediyl))dihexanoate 22b

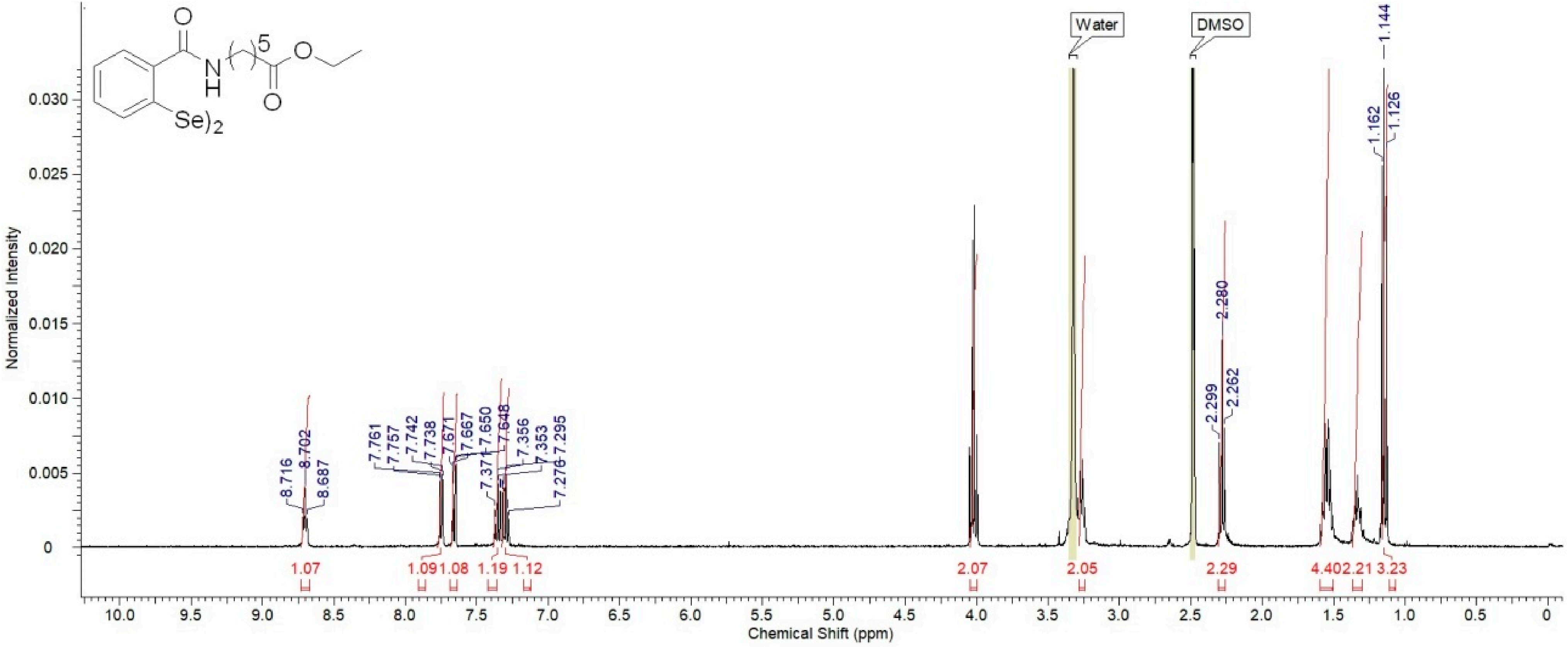

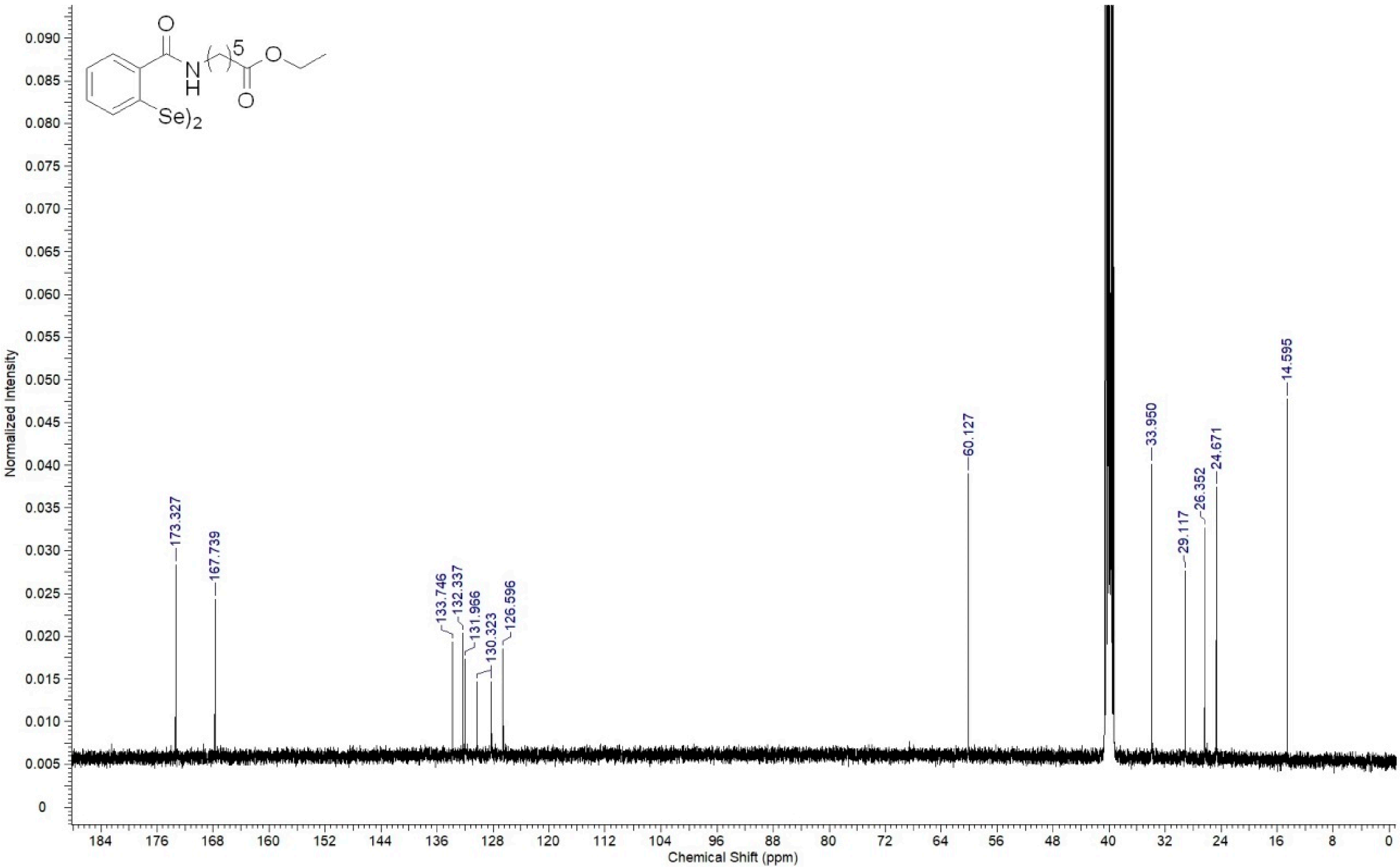

Se)2
173.327
167.739
133.746
132.337
131.966
130.323
126.596
60.127
33.950
29.117
26.352
24.671
14.595
Normalized Intensity
Chemical Shift (ppm)

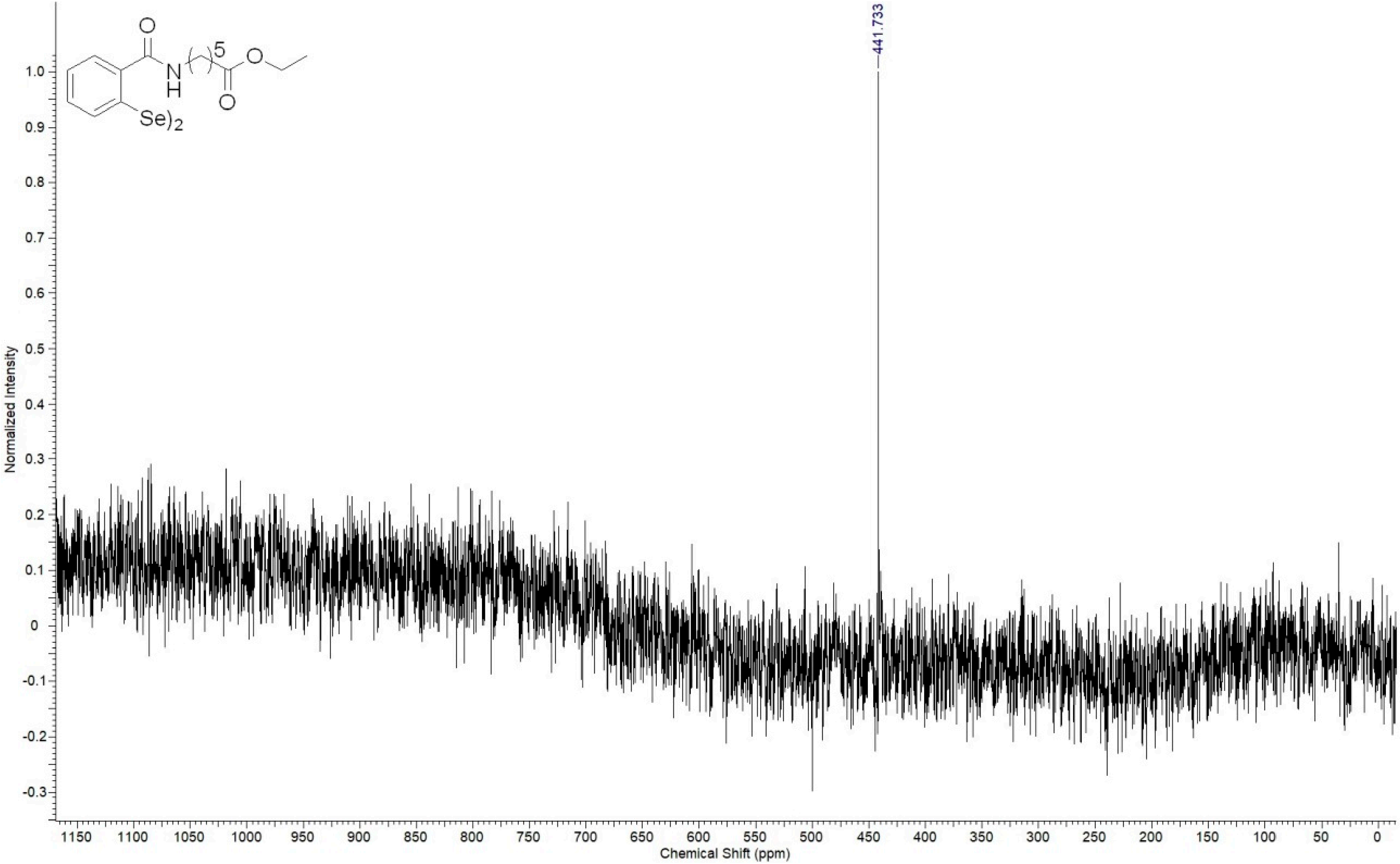

O
N
H
5
O
O
Se)2
441.733
Normalized Intensity
1.0
0.9
0.8
0.7
0.6
0.5
0.4
0.3
0.2
0.1
0
-0.1
-0.2
-0.3
1150 1100 1050 1000 950 900 850 800 750 700 650 600 550 500 450 400 350 300 250 200 150 100 50 0
Chemical Shift (ppm)

Diethyl 8,8'-((2,2'-diselanediylbis(benzoyl))bis(azanediyl))dioctanoate 23b

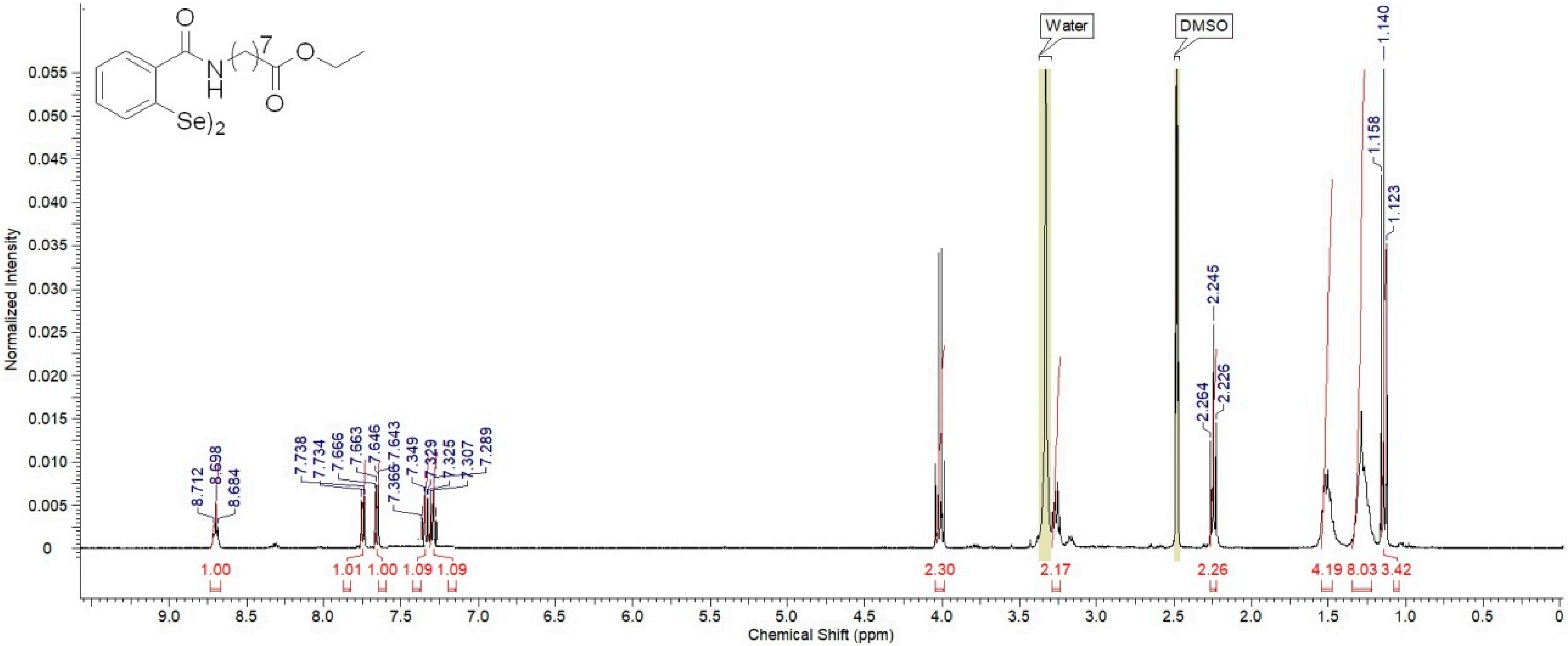

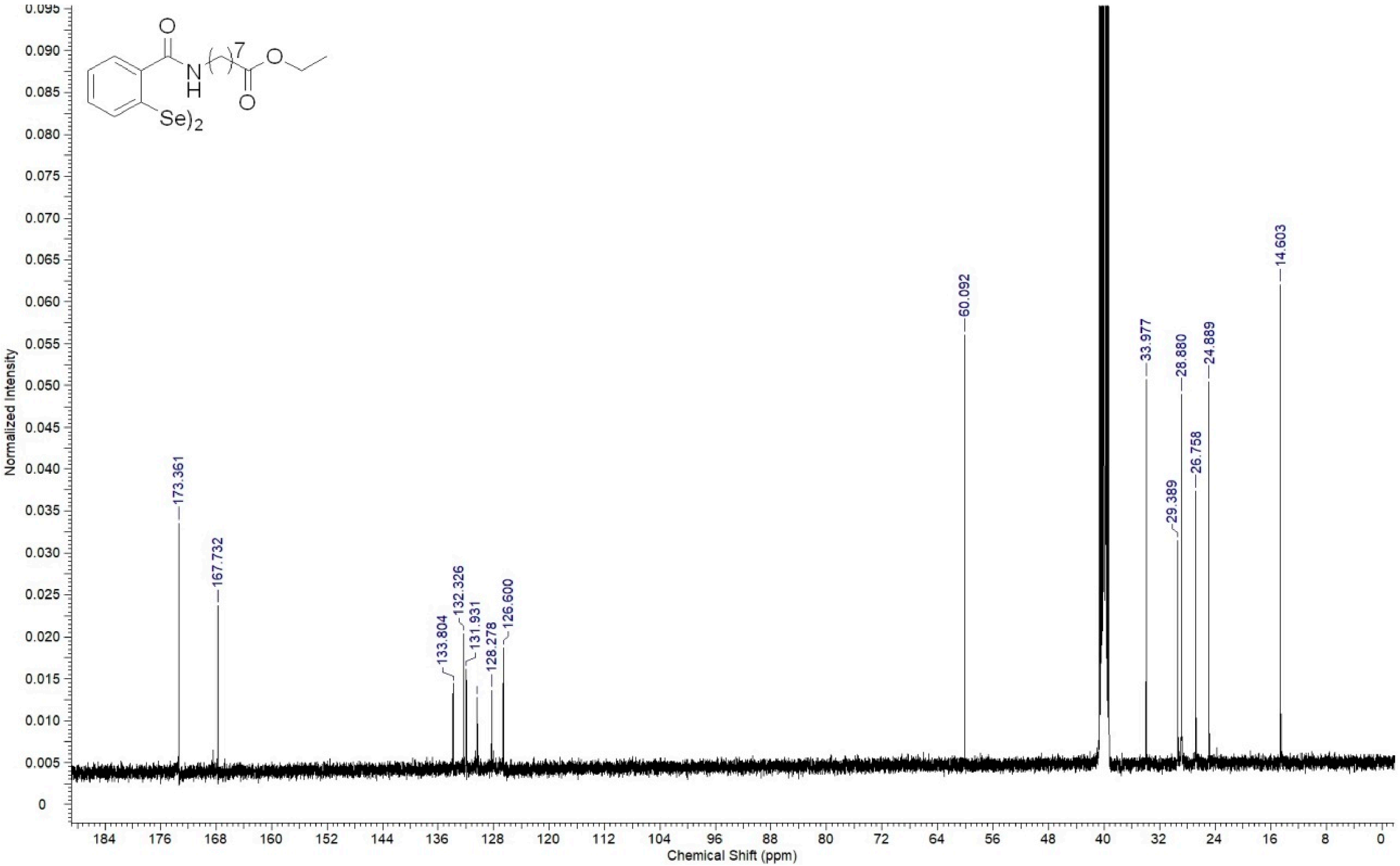

173.361
167.732
133.804
132.326
131.931
128.278
126.600
60.092
33.977
29.389
28.880
26.758
24.889
14.603
Normalized Intensity
Chemical Shift (ppm)
Se)2

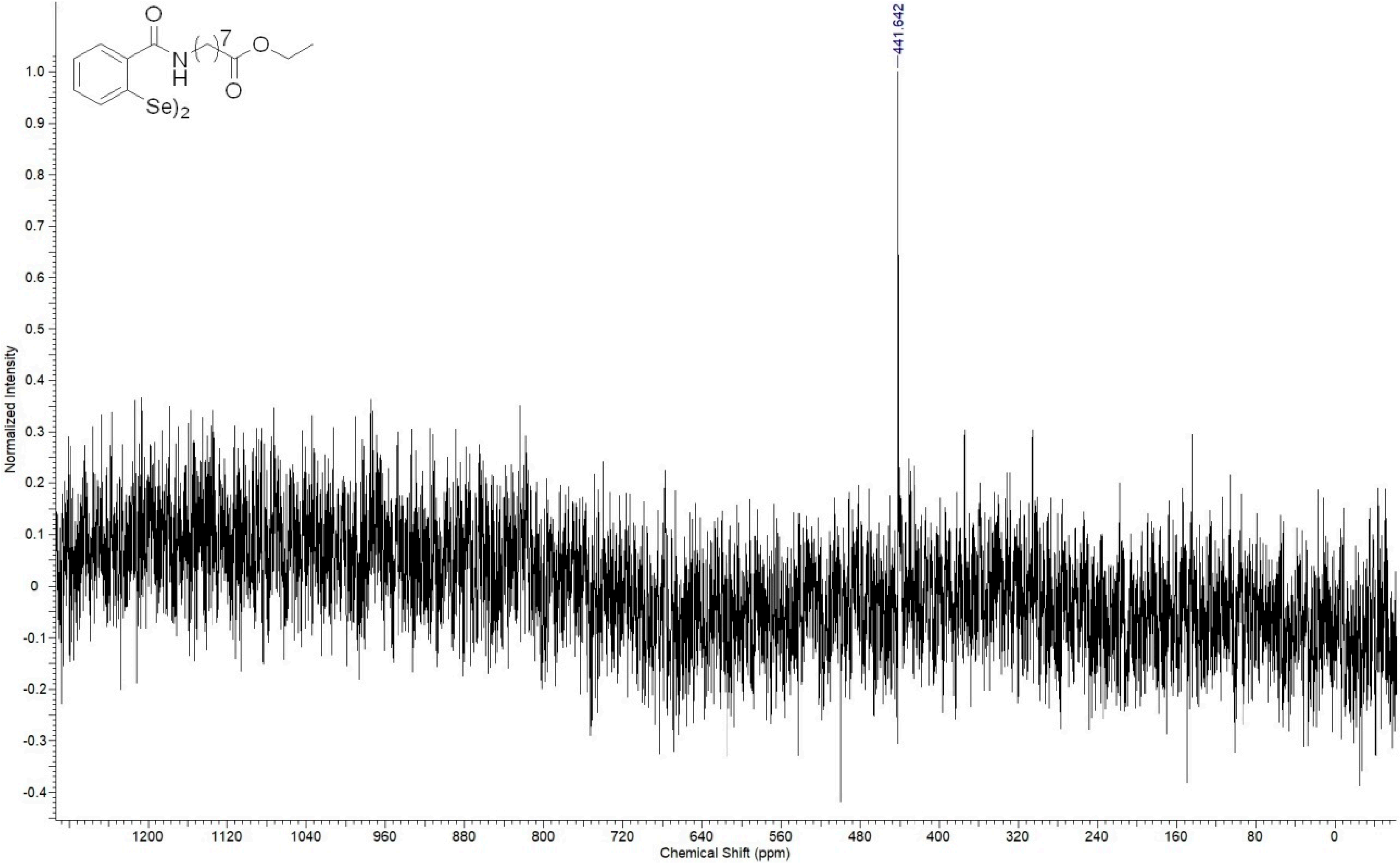

Se)2
N
H
7
O
O
O
441.642
Normalized Intensity
Chemical Shift (ppm)

Diethyl 12,12'-((2,2'-diselanediylbis(benzoyl))bis(azanediyl))didodecanoate 24b

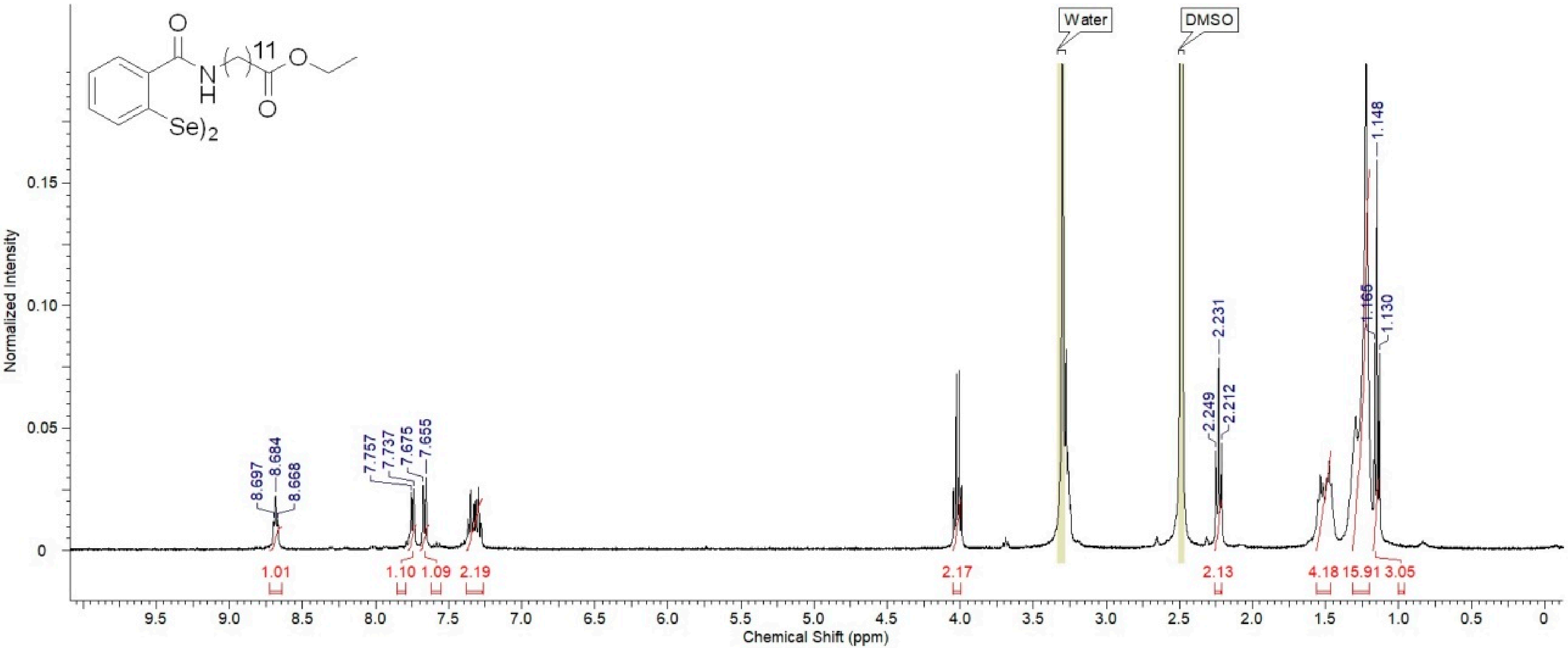

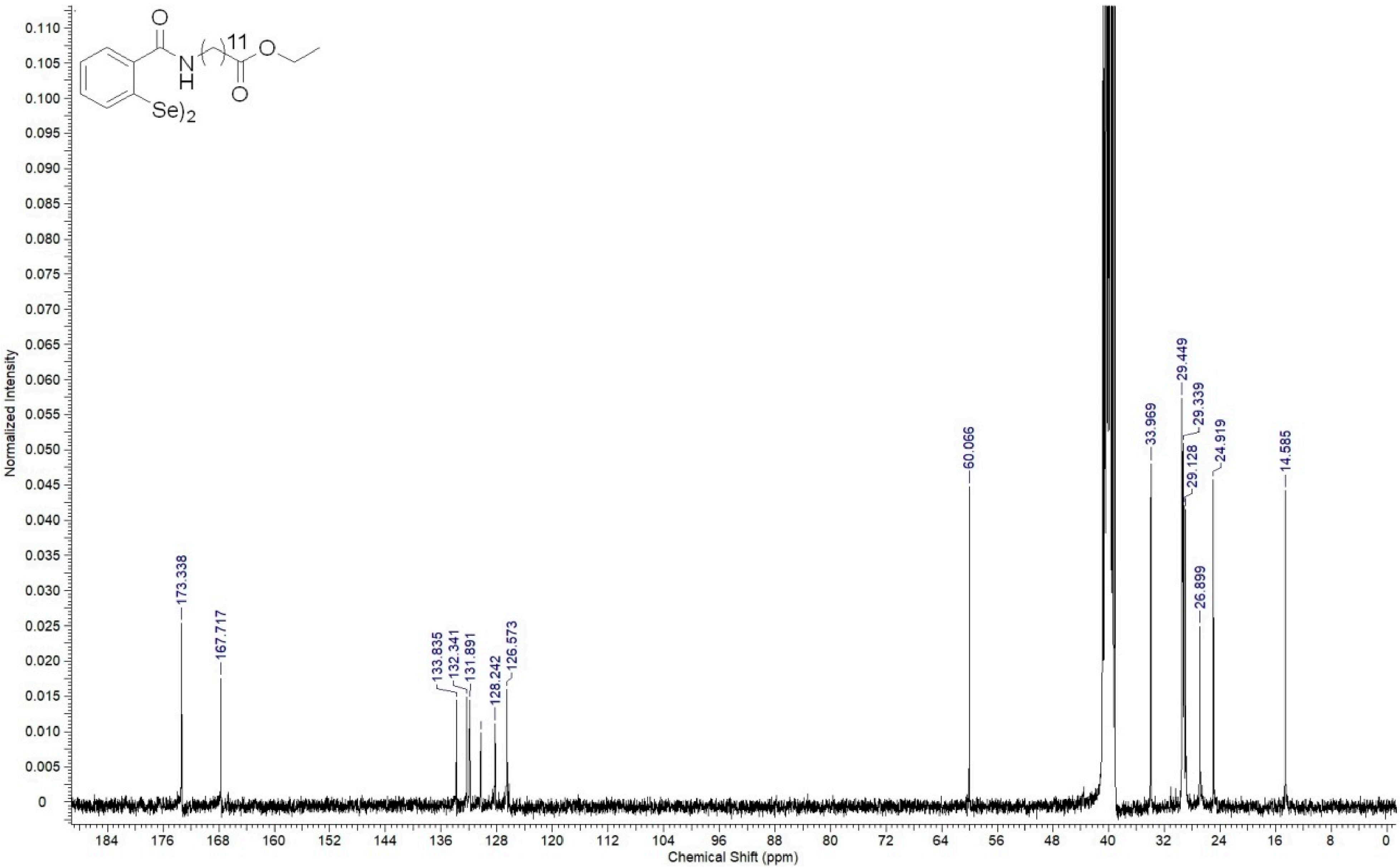

Se)2
11
N
H
O
O
O
Normalized Intensity
Chemical Shift (ppm)
173.338
167.717
133.835
132.341
131.891
128.242
126.573
60.066
33.969
29.449
29.339
29.128
26.899
24.919
14.585

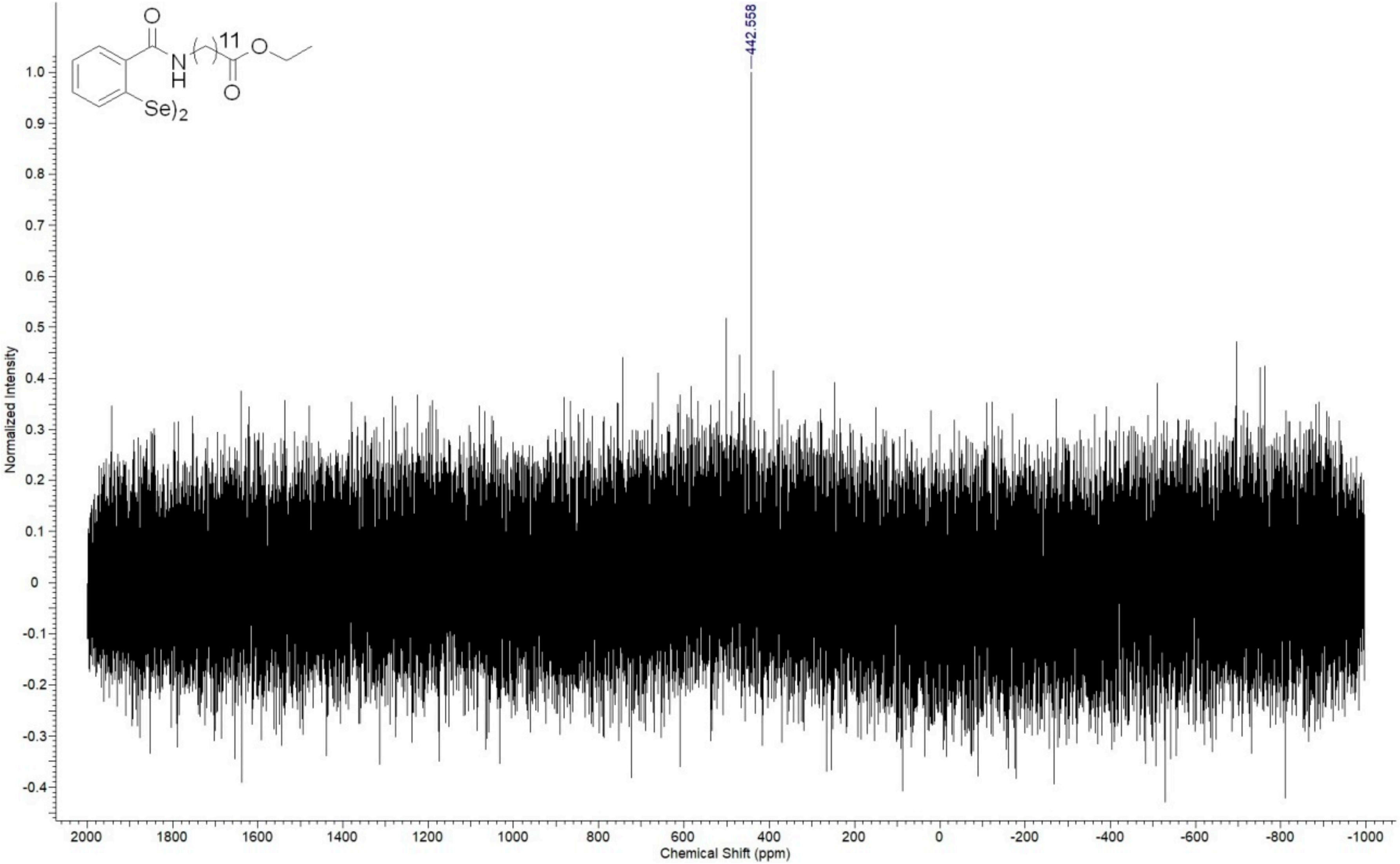

Se)2
11
442.558
Normalized Intensity
Chemical Shift (ppm)

VI. References

1. Welter, A.; Etschenberg, E.; Kuhl, P.; Graf, E.; Dereu, N.; Wendel, A.; Fischer, H.; Christiaens, L. Diselenobis-benzoic acid amides of primary and secondary amines and processes for the treatment of diseases in humans caused by a cell injury, A NATTERMANN & CIE GMBH, US4873350, **1989**, A.
2. Pacuła, A. J.; Ścianowski, J.; Aleksandrzak, K. B. Highly efficient synthesis and antioxidant capacity of *N*-substituted benzisoselenazol-3(2*H*)-ones, *RSC Adv.* **2014**, *4*, 48959-48962.
3. Mlochowski, J.; Kloc, K.; Syper, L.; I., Anna D.; Piasecki, E. Aromatic and Azaaromatic Diselenides, Benzisoselenazolones and Related Compounds as Immunomodulators Active in Humans: Synthesis and Properties, *Liebigs Annalen der Chemie*, **1993**, 12, 1239 – 1244.
